# Discovery of RMC-5552, a Selective Bi-Steric Inhibitor of mTORC1, for the Treatment of mTORC1-Activated Tumors

**DOI:** 10.1021/acs.jmedchem.2c01658

**Published:** 2022-12-19

**Authors:** G. Leslie Burnett, Yu C. Yang, James B. Aggen, Jennifer Pitzen, Micah K. Gliedt, Chris M. Semko, Abby Marquez, James W. Evans, Gang Wang, Walter S. Won, Aidan C. A. Tomlinson, Gert Kiss, Christos Tzitzilonis, Arun P. Thottumkara, James Cregg, Kevin T. Mellem, Jong S. Choi, Julie C. Lee, Yongyuan Zhao, Bianca J. Lee, Justin G. Meyerowitz, John E. Knox, Jingjing Jiang, Zhican Wang, David Wildes, Zhengping Wang, Mallika Singh, Jacqueline A. M. Smith, Adrian L. Gill

**Affiliations:** Revolution Medicines, Incorporated, Redwood City, California 94063, United States

## Abstract

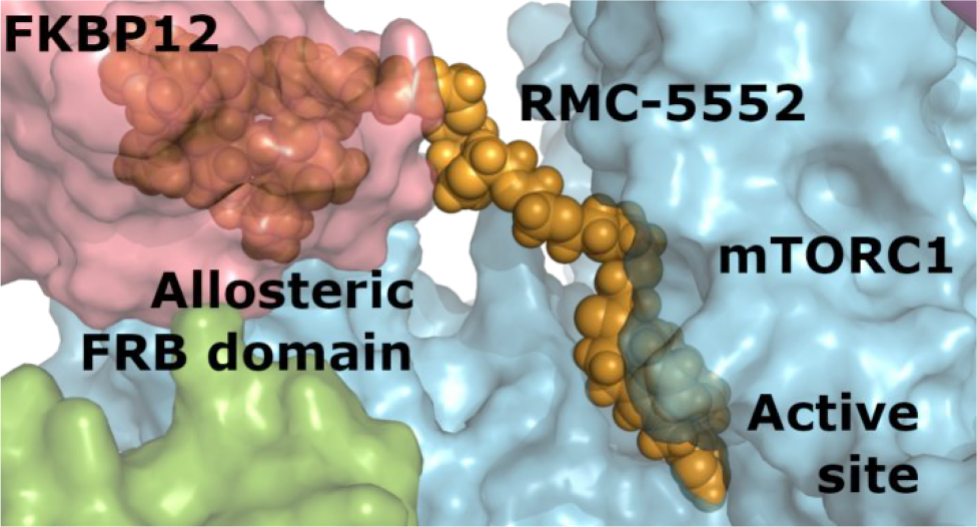

Hyperactivation of mTOR kinase by mutations in the PI3K/mTOR pathway or by crosstalk with other mutant cancer drivers, such as RAS, is a feature of many tumors. Multiple allosteric inhibitors of mTORC1 and orthosteric dual inhibitors of mTORC1 and mTORC2 have been developed as anticancer drugs, but their clinical utility has been limited. To address these limitations, we have developed a novel class of “bi-steric inhibitors” that interact with both the orthosteric and the allosteric binding sites in order to deepen the inhibition of mTORC1 while also preserving selectivity for mTORC1 over mTORC2. In this report, we describe the discovery and preclinical profile of the development candidate RMC-5552 and the in vivo preclinical tool compound RMC-6272. We also present evidence that selective inhibition of mTORC1 in combination with covalent inhibition of KRAS^G12C^ shows increased antitumor activity in a preclinical model of *KRAS*^*G12C*^ mutant NSCLC that exhibits resistance to KRAS^G12C^ inhibitor monotherapy.

## Introduction

**Figure 1:**
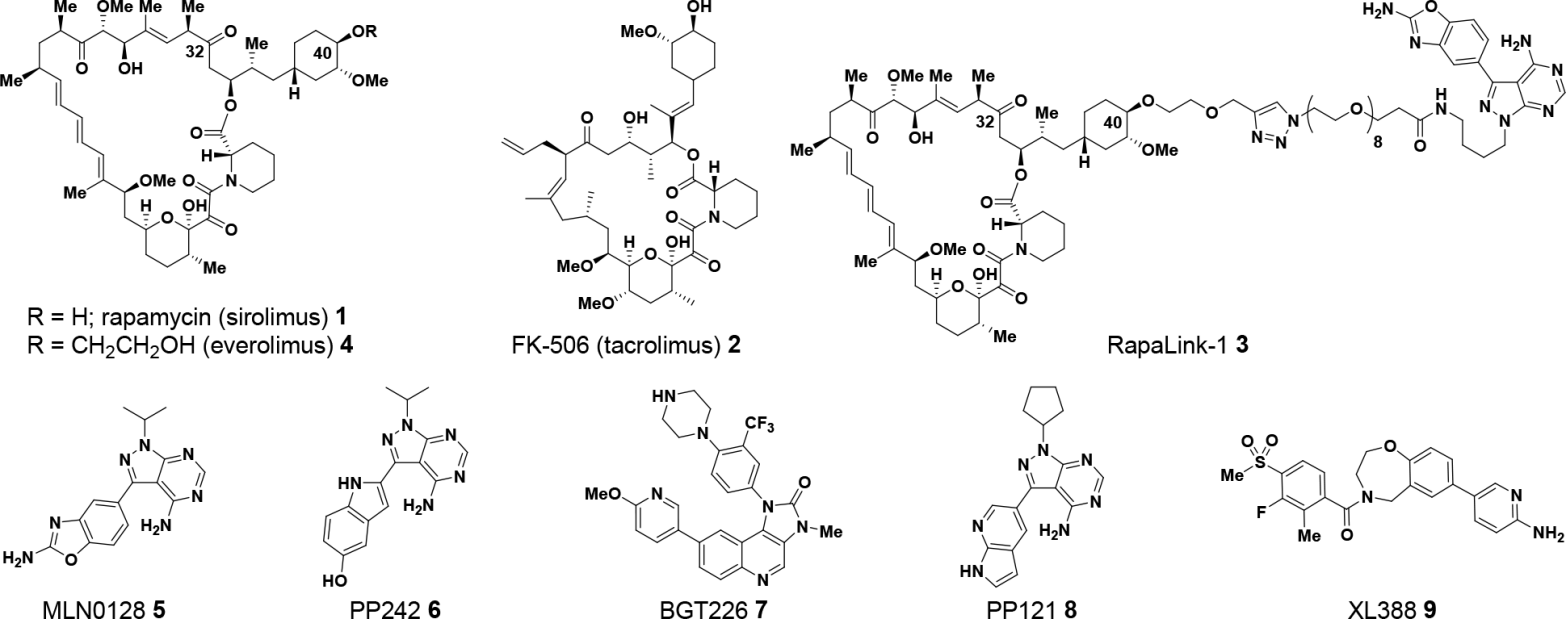
Rapamycin, FK-506, Rapalink-1, and representative mTOR inhibitors.

Rapamycin (AY-22,989; sirolimus) **1**, a naturally occurring macrolide, was isolated in the 1970s from Streptomycete strain AY B-994 (characterized as *Streptomyces hygroscopicus*), cultured from a sample of soil collected from Easter Island (Rapa Nui, Figure 1).^1−6^ At the time of isolation, rapamycin **1** was described as an antifungal agent, with activity against 10 strains of yeast *Candida albicans* (minimum inhibitory concentration of 0.02–0.2 μg/mL), *Microsporum gypseum*, and *Trichophyton granulosum*.^1−7^ Reports of immunosuppressive^8−10^ and anticancer^11−15^ activity with rapamycin followed in the late 1970s and throughout the 1980s. However, it was over a decade before a detailed mechanistic understanding of the biological activity for rapamycin began to be revealed. Rapamycin and a structurally related immuno-suppressive natural product FK-506 (tacrolimus) **2**(16,17) bind to a family of FK binding proteins (FKBP), which catalyze cis–trans isomerization of proline amide bonds found in peptides.^18−20^ The most abundant FKBP in the cytoplasm is a 12 kDa protein termed FKBP12.^21^ FK-506 bound to FKPB12 mediates immunosuppressive activity by binding to calcineurin and preventing translocation of Nuclear factor of activated T-cells (NFAT) into the nucleus.^22^ In contrast, rapamycin **1**, although related in its chemical structure to FK-506 **2**, was recognized as having a differing mechanism of action.^20,23^ Studies in yeast identified a gene encoding for a homologue of FKBP together with two additional genes that participate in rapamycin biology.^24^ The genes were named Target of Rapamycin 1 and 2 (TOR1 and TOR2) with a suggestion that subunits of rapamycin-FKBP12 and TOR interacted as a protein complex.^24^ Thereafter, independent studies identified a mammalian homologue of TOR.^25−27^ In time, this homologue became known as mammalian Target of Rapamycin (mTOR) or, more recently, mechanistic Target of Rapamycin.^28^

mTOR is a 289 kDa serine/threonine protein kinase belonging to the phosphatidylinositol 3-kinase-related kinases (PIKK) family^29^ sitting downstream of receptor tyrosine kinase (RTK) and phosphatidylinositol 3-kinase (PI3K) signaling. mTOR contains kinase^30,31^ and FKBP12-rapamycin-binding (FRB) domains,^32^ along with multiple *N*-terminal Huntington elongation factor 1A-protein phosphatase 2A-A subunit-TOR (HEAT) repeats, FAT (FRAP, ATM, and TRRAP), and C-terminal FAT (FATC) domains, among other important structural elements.^33^ mTOR is recognized to form two distinct complexes, known as (rapamycin-sensitive) mTOR complex 1 (mTORC1) and (rapamycin-insensitive) mTOR complex 2 (mTORC2).^34^ mTORC1 coordinates with auxiliary protein Regulatory-associated protein of mTOR (Raptor)^35^ to phosphorylate multiple substrates, including ribosomal protein S6 kinase (S6K) and eukaryotic initiation factor 4E-binding protein 1 (4EBP1).^36−39^ Phosphorylation of 4EBP1 releases eIF4E, relieving inhibition of cap-dependent translation and driving growth and proliferation of normal and cancer cells.^40^ mTORC2 is defined by the presence of rapamycin-insensitive companion of mTOR (Rictor) and mammalian stress-activated protein kinase interacting protein 1 (Sin1)^41−43^ and phosphorylates and activates AKT in response to cellular stimuli, including activation of PI3K.^44^ mTORC2 activation leads to effects on glucose metabolism, proliferation, cell survival, and growth.^45^

Inhibition of mTOR has been of historical interest to the pharmaceutical industry, with rapamycin **1** being approved as an immunosuppressant in 1999.^46^ More recently, mTOR inhibition as a cancer therapeutic has been of particular interest, especially in the context of effects upon the 4EBP1-eIF4E axis.^40^ There are three broad classes of mTOR inhibitors.^47^ Rapamycin and its analogs (termed rapalogs) are first-generation mTOR inhibitors and inhibit mTORC1-mediated phosphorylation of S6K but only weakly inhibit phosphorylation of 4EBP1 and have negligible effects on mTORC2.^48^ Second-generation mTOR inhibitors, such as sapanisertib (MLN0128, INK128), are ATP-competitive inhibitors of the mTOR kinase and thus inhibit phosphorylation of substrates of mTORC1 and mTORC2, including S6K, 4EBP1, and AKT. The clinical activity of second-generation mTOR inhibitors remains marginal, potentially due to dose-limiting toxicities that prevent optimal inhibition of 4EBP1 phosphorylation in the clinical setting.^49^ In 2016, Shokat and colleagues introduced a third generation of mTOR inhibitor, exemplified by RapaLink-1 **3**,^50,51^ which links an FKBP12-FRB allosteric mTOR inhibitor based on rapamycin together with an active-site (orthosteric) inhibitor, based on sapanisertib.^52,53^ RapaLink-1 **3** inhibits mTOR activity more potently than other mTOR inhibitors, overcomes some mechanisms of resistance, and also shows an approximate 3–4-fold selectivity for inhibition of mTORC1 over mTORC2, as illustrated by inhibition of phosphorylation of 4EBP1 (IC_50_ = 1.7 nM) over inhibition of phosphorylation of AKT (IC_50_ = 6.7 nM) in MDA-MB-468 cells. The therapeutic potential of a bi-steric inhibitor such as RapaLink-1 **3** inspired our own interest in mTOR inhibition.^54−66^ Our aim was to design a compound with further enhanced selectivity for mTORC1 over mTORC2. Such a compound would potently inhibit both S6K and 4EBP1 phosphorylation while limiting unwanted effects on glucose metabolism and relief of AKT-dependent feedback inhibition of receptor tyrosine kinase (RTK) expression that result from inhibition of mTORC2.^67−70^ In this paper, we build upon the findings with RapaLink-1 **3** to describe how each component of the bi-steric molecule (active-site inhibitor, linker, rapamycin core, and chemical handle of attachment) can be modified to obtain a compound with enhanced selectivity for mTORC1 over mTORC2. Such mTORC1-selective bi-steric inhibitors also demonstrate selectivity over related off-target lipid kinases and inhibition of mTORC1-mediated substrate phosphorylation in tumors, which translated to antitumor activity in xenograft models as a single agent and in combination with other targeted inhibitors. Our work culminated in the discovery of a development candidate RMC-5552 **38**, which is currently undergoing evaluation in clinical studies.^71^

## Chemistry

The synthesis of bi-steric compounds containing a modified rapamycin unit as a key constituent presents numerous challenges. For example, rapamycin **1** is a 31-membered lactam–lactone macrolide containing a rich density of carbon–oxygen functional groups, numerous stereodefined olefins, including an acid- and air-sensitive conjugated triene, and a delicate *beta*-keto lactone at the C32 carbonyl that is particularly prone to base-induced elimination of pipecolinate (vide infra).^72,73^ Functionalization of rapamycin to introduce linking groups en route to bi-steric final compounds optimally occurs at precise locations without affecting the stereochemical integrity of the 15-chiral centers nor initiating base- or acid-catalyzed degradation pathways.^72−75^ To complicate matters further, rapamycin and close analogs exist as two interconverting hemiketal structural isomers.^76^

**Scheme 1:**
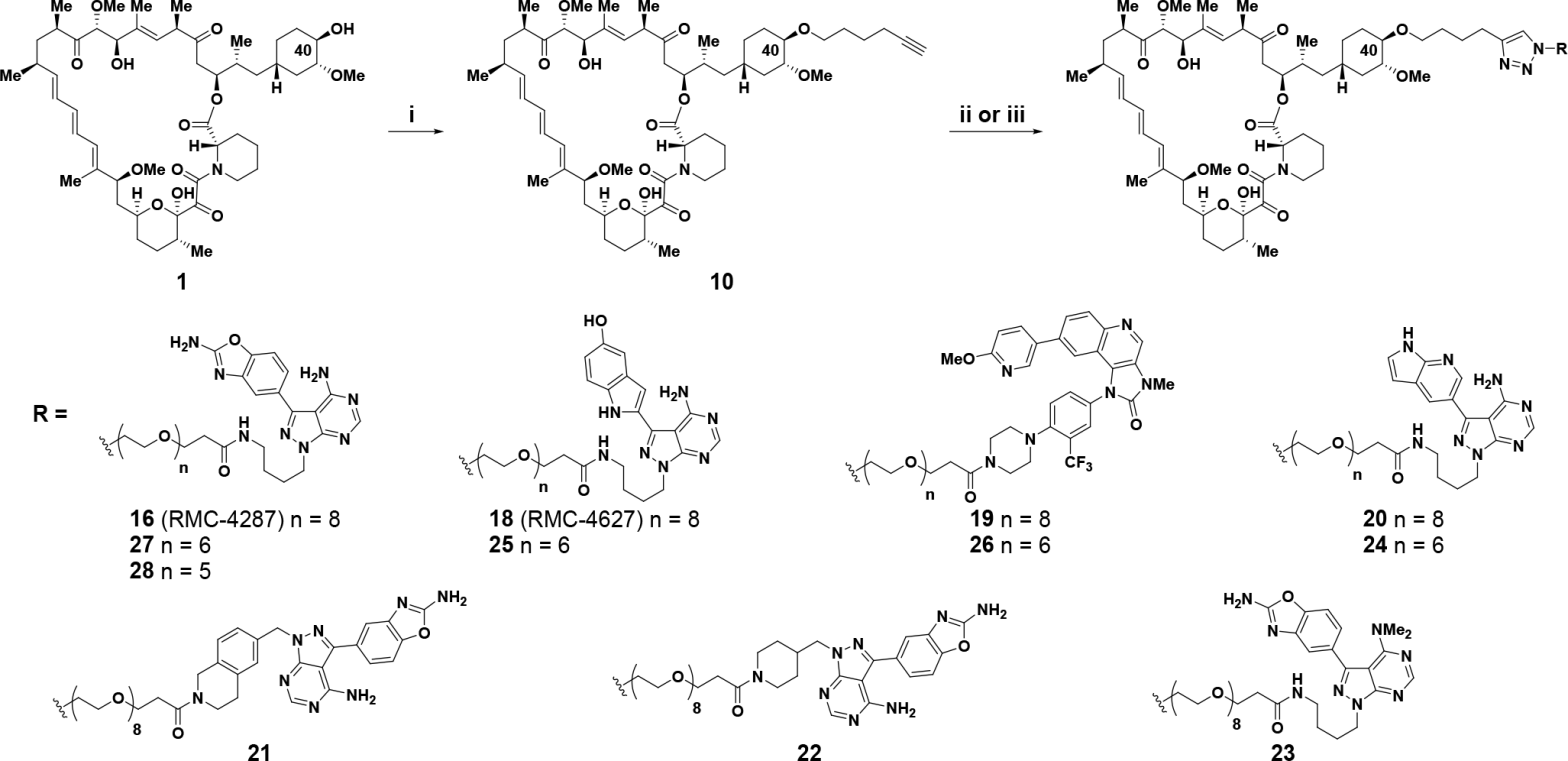
Synthesis of C40-Ether Triazole-Linked Bi-Steric Inhibitors Reagents and conditions: (i) hex-5-yn-1-yl trifluoromethanesulfonate, 2,6-di-*tert*-butyl-4-methylpyridine, DCM, from 0 °C to rt, 30% yield; (ii) RN_3_, Cu(MeCN)_4_PF_6_, TBTA, DMSO, rt, 20–62% yield; (iii) RN_3_, CuSO_4_, sodium ascorbate, MeOH, rt, 18–37% yield.

**Scheme 2:**
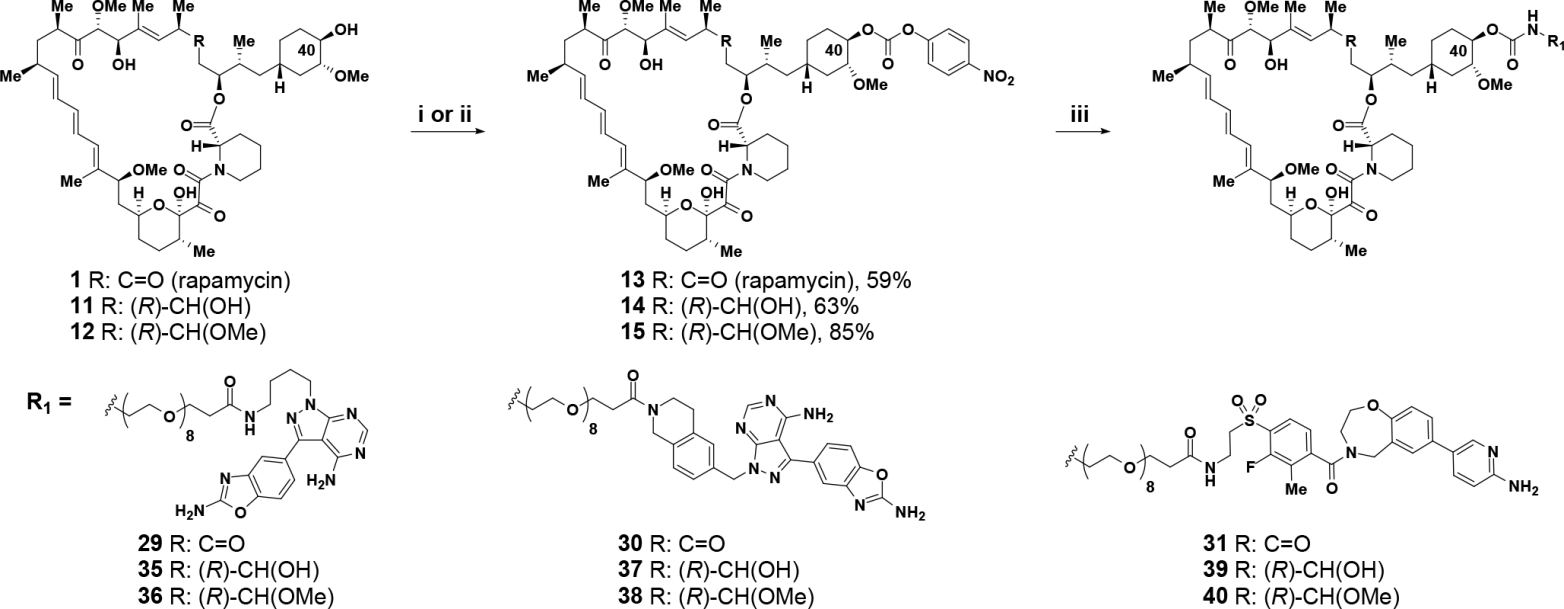
Synthesis of C40-Carbamate-Linked Bi-Steric Inhibitors Reagents and conditions: (i) R = C=O (rapamycin); *p*-nitrophenyl chloroformate, py, DCM, −78 °C, 59% yield. (ii) R = (*R*)-CH(OH); *p*-nitrophenyl chloroformate, py, 4 Å molecular sieves, DCM, from −15 °C to −10 °C, 63% yield or R = (*R*)–CH(OMe), from −10 °C to rt, 85% yield. (iii) R_1_NH_2_, DIPEA, DMA, rt, 36–63% yield.

**Scheme 3:**
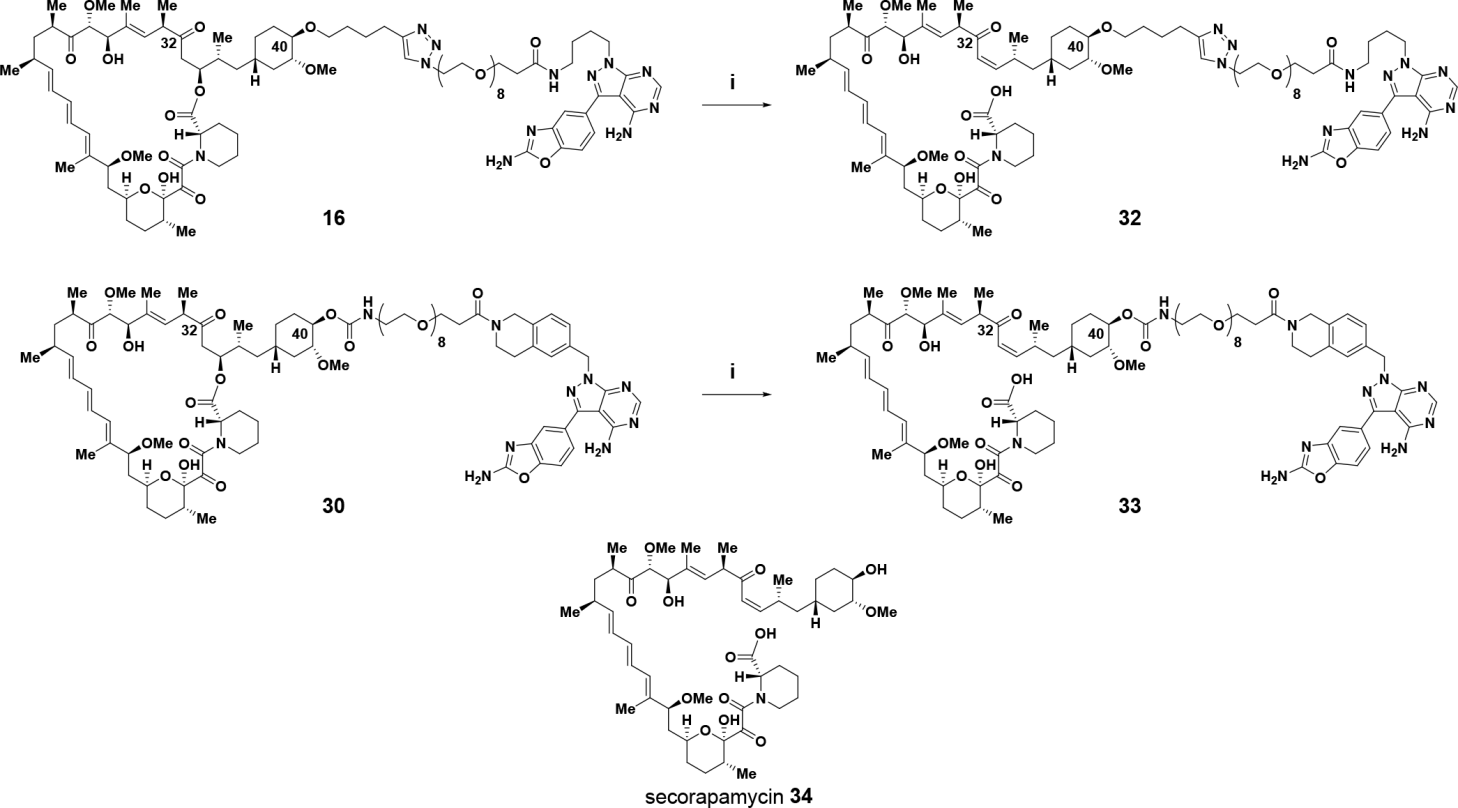
Synthesis of Ring-Opened Seco-Analogs, and Structure of Secorapamycin Reagents and conditions: (i) NH_4_OAc, DMA, 40 °C, 28% yield.

Selective functionalization of the C40 oxygen of rapamycin has been previously described,^77^ and alkylation of this position has been used to prepare the approved compound everolimus **4**.^73,78^ In addition, functionalization of the C40 oxygen in rapamycin was also used to prepare RapaLink-1 **3**.^50,51^ Building upon these results, we prepared numerous bi-steric inhibitors using the chemistry outlined in Schemes 1 and 2. For bi-steric inhibitors containing a C40-ether linkage with an appendant triazole, the C40-hex-5-yn-1-yl rapamycin **10** component was prepared from rapamycin **1** (30% yield) by alkylation with freshly purified hex-5-yn-1-yl trifluoromethanesulfonate and 2,6-di-*tert*-butyl-4-methylpyridine (Scheme 1).^54^ Final triazole-containing bi-steric compounds **16**–**28** were synthesized via a copper-catalyzed “click” Huisgen cycloaddition^79^ from C40-hex-5-yn-1-yl rapamycin **10** with active-site coupling partners containing an azido PEG-linked side chain, the synthesis of which are available as Supporting Information (Scheme 1). A later generation of bi-steric inhibitors required access to a rapamycin analog functionalized at C40 with a *p*-nitrophenyl (PNP) carbonate.^80^ For rapamycin itself, the C40-PNP carbonate **13** was synthesized in 59% yield from rapamycin **1** and *p*-nitrophenyl chloroformate using pyridine as base (Scheme 2).^80^ Similar reaction conditions, with the addition of 4 Å molecular sieves, could provide C40-PNP carbonates of C32-hydroxy rapamycin **14**(81−83) and C32-methoxy rapamycin **15**(54) in 63% and 85% yield, respectively (Scheme 2). The appropriate C40-PNP carbonates **13**–**15** were used to form carbamate-linked bi-steric inhibitors **29**–**31** and **35**–**40** by reaction with active-site counterparts (synthesis available as Supporting Information), which contained a PEG linker terminated with an amine group (Scheme 2). The ring-opened seco-products **32** and **33** were synthesized from bi-steric inhibitors **16** and **30** by reaction with ammonium acetate in dimethylacetamide at 40 °C in a manner analogous to the formation of secorapamycin **34** from rapamycin **1** (Scheme 3).^84^

Similar to rapamycin **1**(76) and everolimus **4**,^85^ final bi-steric compounds were found to exist as interconverting mixtures of ketal structural isomers with the specified drawn ketal structural isomer as the overwhelming component.^86^

## Results and Discussion

### Structure–Activity Relationship (SAR) Studies

RapaLink-1 **3** conjugates rapamycin to an active-site ATP inhibitor through an extended linker system that allows the resulting inhibitor to bind both the FRB domain and the active site of mTORC1 or the FRB domain and the active site of mTORC2.^50^ For RapaLink-1 **3**, sapanisertib^52,53^ was selected as the mTOR active-site inhibitor for its potency and relative selectivity for mTOR kinase.^50^ In addition, the *N*-1 position of the pyrazole nitrogen within sapanisertib is solvent exposed and orientates toward the rapamycin-FRB motif, thus providing a convenient handle for attachment of a nonperturbing linker. Appropriate solvent-exposed positions with desirable orientation can also be found within rapamycin. The C40 hydroxyl group of rapamycin is exposed to solvent and orientates toward the ATP binding site of mTOR. The linker of RapaLink-1, which contains 39 heavy atoms, was designed through molecular modeling studies of prospective inhibitors containing linker lengths ranging from 10 to 40 heavy atoms with linker lengths less than approximately 25 heavy atoms calculated to result in less favorable energetics.^50^

**Figure 2:**
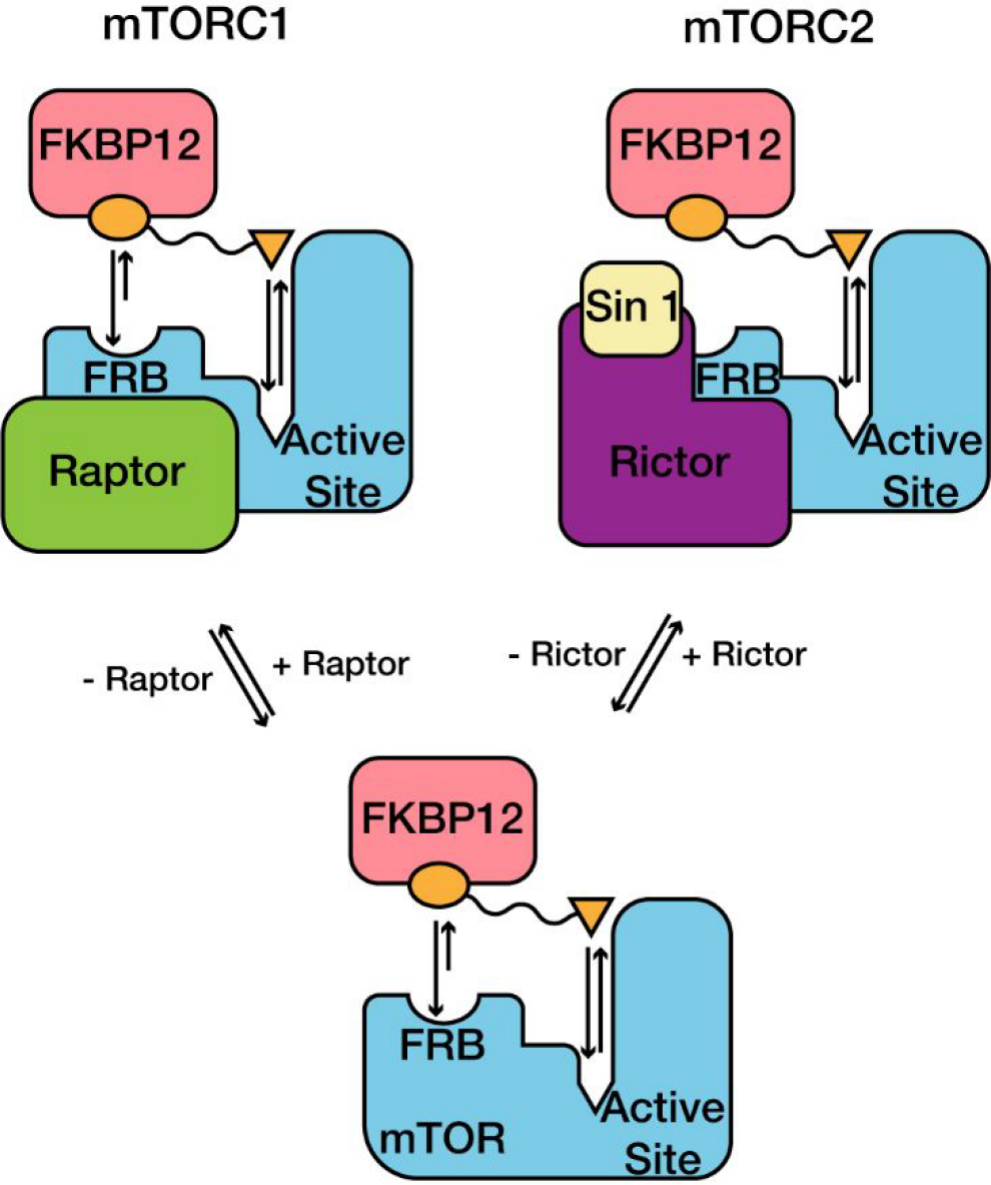
Structural representation of binding sites for FKBP12-rapamycin and active-site inhibitors in mTORC1 and mTORC2. Rapamycin has reduced affinity for mTORC2 due to partial occlusion of the FKBP12-rapamycin binding (FRB) domain, while active-site inhibitors have similar affinity for both complexes. Reprinted by permission from Springer Nature ref (58). Copyright 2021.

As previously outlined, mTORC1 associates with auxiliary protein Raptor (149 kDa) while mTORC2 associates with the auxiliary proteins Rictor (192 kDa) and Sin1. We reasoned that the reduced affinity of rapamycin for mTORC2, in comparison to mTORC1, may be due to a partial occlusion of the FKBP12-rapamycin binding (FRB) motif in mTORC2 by the Rictor-Sin1 complex (Figure 2). Recent structural studies of mTORC1 and mTORC2 with cryogenic electron microscopy (cryo-EM) have supported this selectivity model and revealed the extent of FRB occlusion in mTORC2.^87,88^ For instance, Scaiola et al. showed that the C-terminal (CT) domain of Rictor sits on top of the mTOR FRB domain in mTORC2, blocking the binding of FKBP-rapamycin to mTORC2 and explaining mTORC2 insensitivity to rapamycin.^88^ These studies supported our rationale that the structural differences between mTORC1 and mTORC2 could be exploited further to increase the selectivity of a bi-steric hybrid. Therefore, we applied a rational drug design strategy to improve on the selectivity by systematically tuning the affinities of the rapamycin core and active-site binding moieties in the bi-steric molecule and also examined the effect of linker length and the chemical handle of attachment to rapamycin.

Our SAR studies with **3** resulted in an early observation that an oxygen atom between the C40 position of rapamycin and a triazole group could be replaced with carbon to give compound **16** (RMC-4287, Table 1, entry 4).^58^ This change did not appreciably alter the mTORC1 inhibition or the mTORC1/mTORC2 selectivity when compared with RapaLink-1 **3** (entry 1), as measured by comparing the potencies for inhibition of 4EBP1 T37/T46 phosphorylation (mTORC1) and AKT S473 phosphorylation (mTORC2) in MDA-MB-468 breast cancer cells. Improved mTORC1/mTORC2 selectivity was noted when the active-site inhibitor was changed to a version of PP242, a close relative of sapanisertib, in both oxygen^51^ and carbon C40-triazole spacers (entries 5 and 6). The resulting bi-steric inhibitors **17**(51) and **18** (RMC-4627)^58^ were approximately equipotent with RapaLink-1 **3** for mTORC1 inhibition (p4EBP1 IC_50_ = 2.5 nM for **17**, 1.4 nM for **18**, and 1.7 nM for RapaLink-1 **3**) but showed reduced inhibition of mTORC2 signaling, cf. RapaLink-1 **3** (pAKT IC_50_ = 24 nM for **17**, 18 nM for **18**, and 6.7 nM for RapaLink-1 **3**), thereby improving the mTORC1/mTORC2 ratio from ca. 4-fold for RapaLink-1 (entry 1) up to approximately 13-fold for bi-steric compound **18** (entry 6). This observation necessitated the investigation of a larger set of active-site ATP inhibitors in our bi-steric ensembles with a predominant focus on carbon-linked analogs. To enable an appropriate reference, where possible, biological activity for the active-site inhibitor is also included in Table 1. When conjugated to an active-site inhibitor different from that present in RapaLink-1, most bi-steric compounds showed improved mTORC1/mTORC2 selectivity. For example, a bi-steric compound using a modified version of BGT226 as the active-site inhibitor **19** (entry 8) exhibited high potency for inhibition of mTORC1 signaling (p4EBP1 IC_50_ = 0.06 nM) while also demonstrating good selectivity over inhibition of mTORC2 signaling (pAKT IC_50_ = 0.52 nM; mTORC1/mTORC2 ratio of 8.7). Even greater selectivity for mTORC1/mTORC2 inhibition was realized when a bi-steric compound contained an active-site inhibitor derived from PP121, leading to compound **20** (entry 10) with mTORC1 p4EBP1 IC_50_ = 1.0 nM and mTORC2 pAKT IC_50_ = 17 nM (mTORC1/mTORC2 ratio of 17.0). The mTORC1/mTORC2 ratio could be increased further again if a tetrahydroisoquinoline-linked and rigidified version of MLN (“rigid-MLN”) was used in the bi-steric construct to give compound **21** (entry 12), exhibiting mTORC1 p4EBP1 IC_50_ = 0.44 nM and mTORC2 pAKT IC_50_ = 8.8 nM (mTORC1/mTORC2 ratio of 20). We believe the higher level of mTORC1/mTORC2 selectivity of bi-steric constructs containing an active-site inhibitor based around “rigid-MLN” (Table 1, entry 12; mTORC1/mTORC2 ratio of 20), compared with a bi-steric compound containing an active-site inhibitor based around MLN, as in RapaLink-1 **3** (Table 1, entry 1; mTORC1/mTORC2 ratio of 3.9), can be attributed to a more favorable orientation effect toward the less sterically encumbered mTORC1 complex (vide infra). However, not all modifications to MLN resulted in improved selectivity. For example, bi-steric compound **22** (entry 13), containing a piperidine-linked “modified-MLN” active-site inhibitor, exhibited an mTORC1/mTORC2 ratio of 2.7, which is approximately the same level of selectivity to matched-pair RapaLink-1 **3** (entry 1). Also of note was the effect of the *N*,*N*-dimethylation of MLN-derived active-site inhibitors when applied to a bi-steric compound. Compound **23** was inactive up to a maximal test concentration of 1 μM against p4EBP1 and pAKT inhibition (entry 14), thus demonstrating the importance of the primary amino-pyrimidinyl group in MLN-derived active-site inhibitors.

**Table 1:**
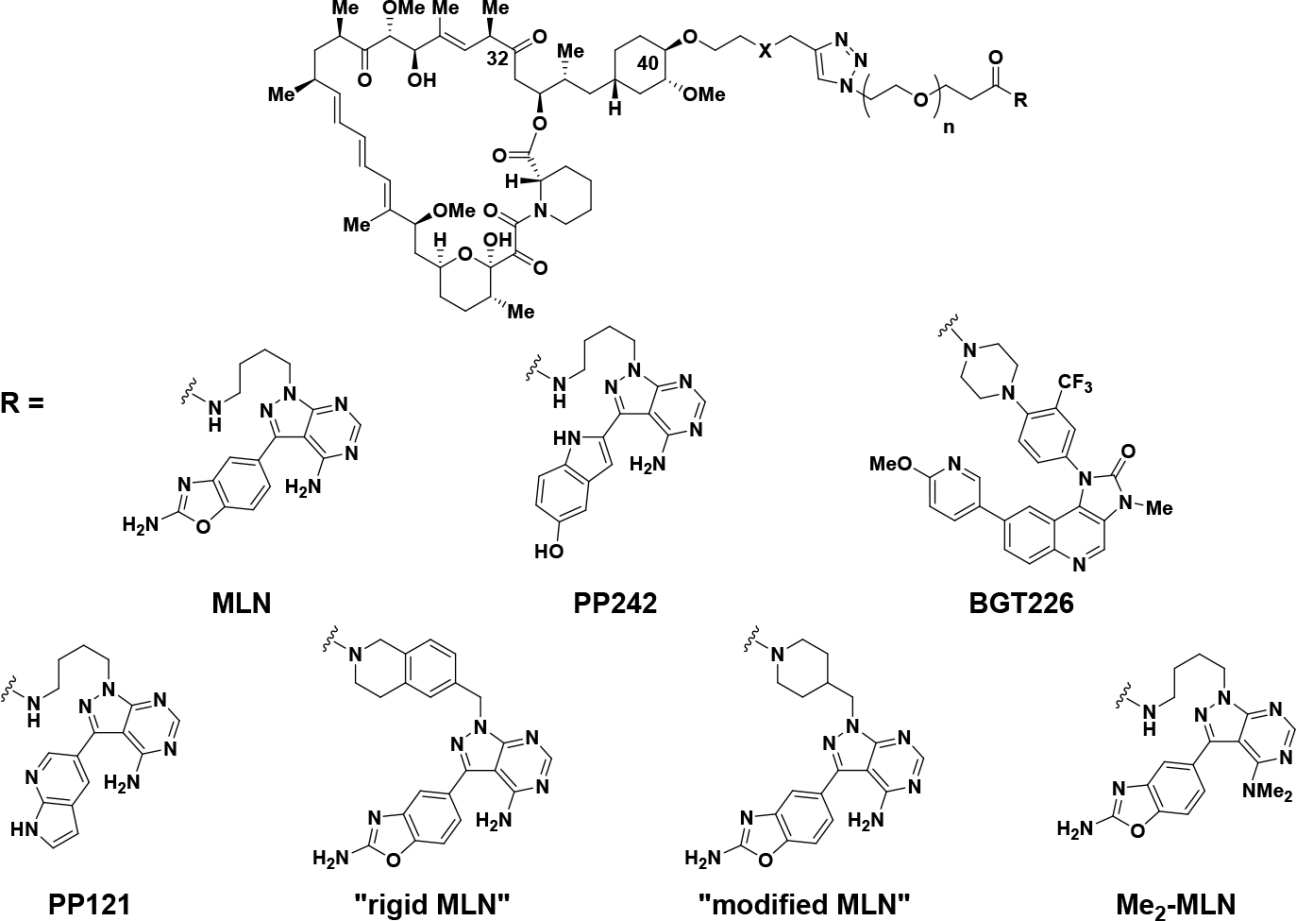
SAR of Active-Site Inhibitors with the C40-Ether Chemical Handle

entry	compound no.	X	PEG repeats	active-site inhibitor	pS6K IC_50_ (nM)	p4EBP1 IC_50_ (nM)	pAKT IC_50_ (nM)	selectivity
1	**3** (RapaLink-1)^50^	O	8	MLN	0.93	1.7	6.7	3.9
2	**4** (everolimus)				0.07	>1000	>1000	
3	**5** (sapanisertib)				0.69	19	1.8	0.1
4	**16** (RMC-4287)	C	8	MLN	0.42	1.1	3.1	2.8
5	**17**(51)	O	8	PP242	0.47	2.5	24	9.6
6	**18** (RMC-4627)	C	8	PP242	0.28	1.4	18	12.9
7	**6** (PP242)				33	320	38	0.1
8	**19**	C	8	BGT226	0.02	0.06	0.52	8.7
9	**7** (BGT226)				0.27	4.4	1.4	0.3
10	**20**	C	8	PP121	0.22	1.0	17	17.0
11	**8** (PP121)				86	>1000	100	
12	**21**	C	8	“rigid MLN”	0.13	0.44	8.8	20.0
13	**22**	C	8	“modified-MLN”	0.16	0.67	1.8	2.7
14	**23**	C	8	“Me_2_-MLN”	0.29	>1000	>1000	

Next, we examined the effect of the PEG linker chain length on the biological activity and mTORC1/mTORC2 selectivity of bi-steric inhibitors (Table 2). As a general trend, reducing a PEG linker from eight (8) PEG units resulted in a lower inhibitory activity of mTORC1, as measured by p4EBP1 inhibition.^50^ Inhibition of AKT phosphorylation by mTORC2 was also reduced with a shorter PEG chain length. However, mTORC2 inhibition was shifted less than mTORC1 inhibition, generally resulting in diminished mTORC1/mTORC2 selectivity ratios for bi-steric inhibitors containing a linker with fewer than eight (8) PEG units. This sensitivity to the PEG chain length was most pronounced for the more selective bi-steric inhibitors containing active-site inhibitors with moderate intrinsic potency toward mTORC2 inhibition. For example, in a bi-steric compound containing PP121 as the mTOR active-site inhibitor, varying the PEG chain length from 8 PEG units to 6 PEG units resulted in a >30-fold reduction in mTORC1 p4EBP1 activity (p4EBP1 IC_50_ from 1.0 nM for 8 PEG units to 31 nM for 6 PEG units; **20**, entry 1 and **24**, entry 2). Although mTORC2 activity was also reduced with a shorter 6 PEG unit linker (pAKT IC_50_ = 17 nM for **20** containing 8 PEG units and pAKT IC_50_ = 110 nM for **24** containing 6 PEG units; entries 1 and 2), the relative shift was lower than the mTORC1 p4EBP1 activity. Thus, the net effect was both to reduce on-target mTORC1 activity and to diminish the mTORC1/mTORC2 selectivity ratio (from 17 to 3.5) upon reducing the PEG linker from 8 to 6 PEG units (entries 1 and 2). A similar observation was apparent when a bi-steric compound utilized PP242 as the active-site inhibitor (entries 3 and 4). In this case, on-target mTORC1 activity dropped >250-fold (p4EBP1 IC_50_ = 1.4 nM to 370 nM) and the mTORC1/mTORC2 selectivity ratio dropped from approximately 13 to 0.3 upon changing the PEG chain length from 8 to 6 PEG units (compounds **18** and **25**, entries 3 and 4). Interestingly, a bi-steric compound containing BGT226 as the active-site inhibitor and with 6 instead of 8 PEG units in the linker resulted in a slight reduction of on-target mTORC1 activity (p4EBP1 IC_50_ = 0.06 nM for 8 PEG linker, cf. p4EBP1 IC_50_ = 0.08 nM for 6 PEG linker) and yet had little effect on the mTORC1/mTORC2 selectivity ratio (compounds **19** and **26**, entries 5 and 6). Finally, for a bi-steric compound using MLN as the active-site inhibitor, a reduction of PEG-linker length from 8 to 6 PEG units did not change on-target mTORC1 activity significantly (p4EBP1 IC_50_ = 1.1 nM in each instance; entries 7 and 8). Surprisingly, the mTORC1/mTORC2 selectivity ratio improved slightly for the 6 PEG linker, compound **27**, over the 8 PEG linker, compound **16** (entries 7 and 8). However, when the linker length was reduced further to 5 PEG units to give compound **28**, a significant diminution of on-target mTORC1 activity (p4EBP1 IC_50_ = 25 nM) and a lowering of the mTORC1/mTORC2 selectivity ratio was again observed (entry 9), demonstrating a general trend toward reduced mTORC1 inhibitory potency and mTORC1/mTORC2 selectivity with shorter linker lengths. Of all of the linker lengths investigated, our studies showed a preference for 8 PEG units, particularly for more selective bi-steric inhibitors.

**Table 2:**
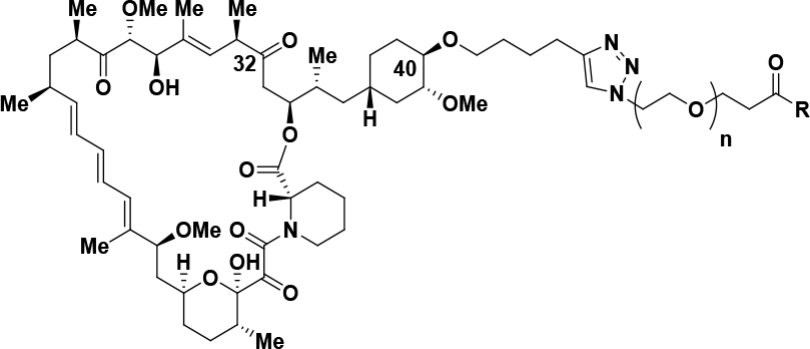
SAR of PEG Linker Length and Active-Site Inhibitors

entry	compound no.	PEG repeats	active-site inhibitor	pS6K IC_50_ (nM)	p4EBP1 IC_50_ (nM)	pAKT IC_50_ (nM)	selectivity
1	**20**	8	PP121	0.22	1.0	17	17.0
2	**24**	6		0.24	31	110	3.5
3	**18**	8	PP242	0.28	1.4	18	12.9
4	**25**	6		0.59	370	120	0.3
5	**19**	8	BGT226	0.02	0.06	0.52	8.7
6	**26**	6		0.03	0.08	0.74	9.3
7	**16**	8	MLN	0.42	1.1	3.1	2.8
8	**27**	6		0.26	1.1	5.2	4.7
9	**28**	5		0.37	25	33	1.3

We also examined the effect of removing a triazole in the linker. The inspiration behind this investigation was to identify moieties that may be attached to the C40 oxygen of rapamycin more efficiently than an ether, which suffered from poor yields of attachment and difficulty with purification of the product. In addition, formation of the triazole ring requires an azide-containing precursor that introduces potential safety concerns when larger scale preparations are required. An attractive solution was found by linking the C40 oxygen of rapamycin via a carbamate group, which could be attached reproducibly and in high yield with well-known *p*-nitrophenoxy chloroformate chemistry (Scheme 2).^80^ We were gratified to observe that the resulting carbamate-linked bi-steric inhibitors were potent mTORC1 inhibitors with good mTORC1/mTORC2 selectivity (Table 3). For instance, carbamate-linked bi-steric inhibitors **29** and **30**, which used MLN (entry 1) or “rigid-MLN” (entry 2) as the active-site inhibitor, respectively, exhibited potent mTORC1 inhibition (p4EBP1 IC_50_ = 1.7 nM for **29**, and p4EBP1 IC_50_ = 0.42 nM for **30**). The mTORC1 activity was comparable to triazole-linked counterparts **16** (p4EBP1 IC_50_ = 1.1 nM; Table 1, entry 4) and **21** (p4EBP1 IC_50_ = 0.44 nM; Table 1, entry 12), and mTORC1/mTORC2 selectivity was broadly similar (compare Table 3, entries 1 and 2, with Table 1, entries 4 and 12). In addition, bi-steric inhibitor **31**, which contains an active-site inhibitor based on XL388 (entry 3), also displayed potent on-target mTORC1 inhibition (p4EBP1 IC_50_ = 0.70 nM) with a moderate degree of mTORC1/mTORC2 selectivity (ratio = 4.1).

**Table 3:**
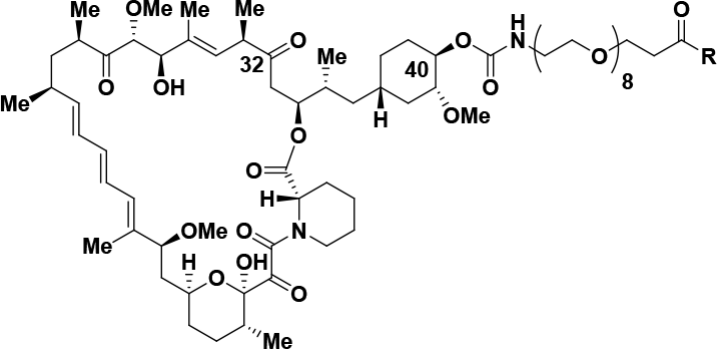
SAR of Active-Site Inhibitors with the C40-Carbamate Chemical Handle

entry	compound no.	PEG repeats	active-site inhibitor	pS6K IC_50_ (nM)	p4EBP1 IC_50_ (nM)	pAKT IC_50_ (nM)	selectivity
1	**29**	8	MLN	0.61	1.7	3.6	2.1
2	**30**	8	“rigid MLN”	0.19	0.42	4.7	11.2
3	**31**	8	XL388	0.23	0.70	2.9	4.1

**Figure 3:**
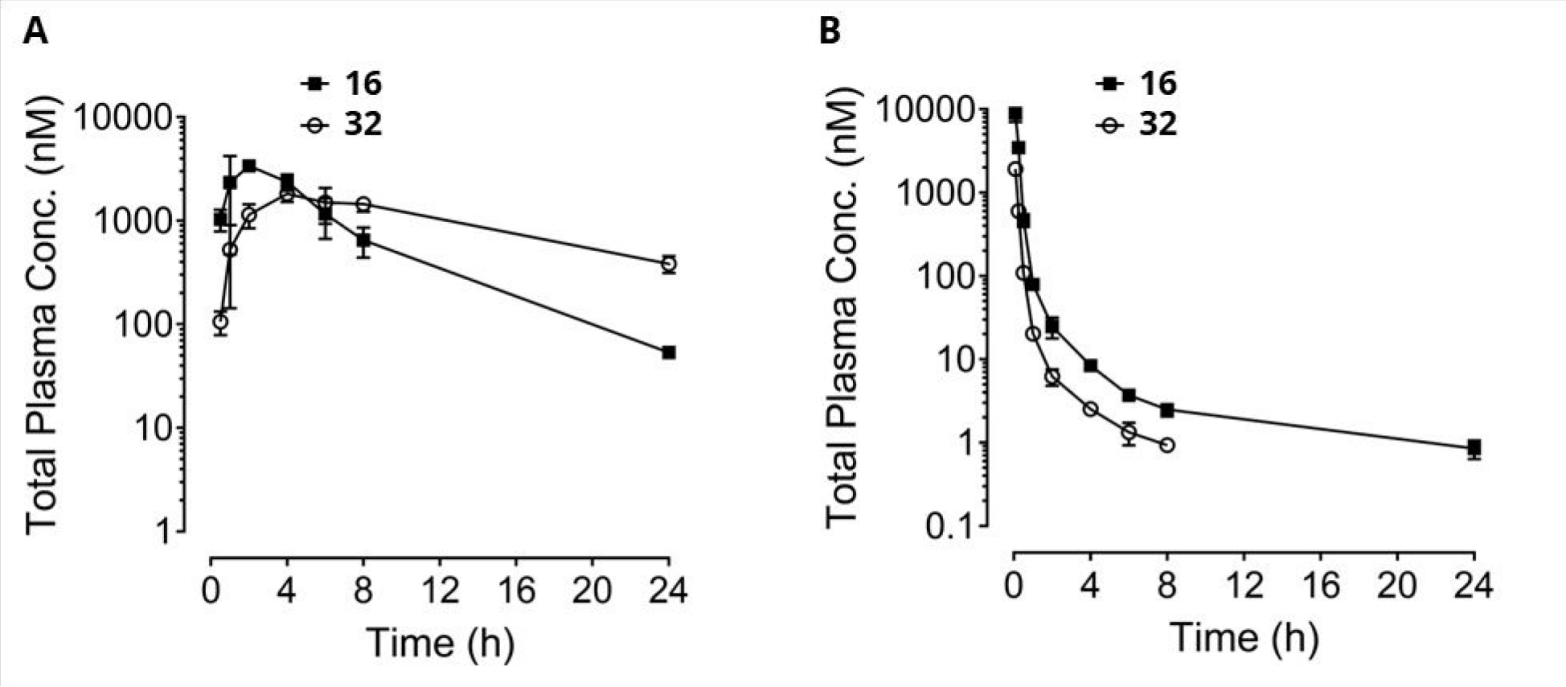
Bi-steric inhibitor **16** undergoes ring opening of the rapamycin macrocycle in vivo to form ring-opened **32**. (A) Mouse PK: male Balb/c mice (*n* = 3), IP = 3 mg/kg. (B) Rat PK: male Sprague–Dawley rats (*n* = 3), IV bolus = 1 mg/kg; vehicle = transcutol/solutol HS15/H_2_O 5%/5%/90% (v,w,v).

With a number of selective bi-steric inhibitors with potent cellular activity in hand, we began to investigate the pharmacokinetic properties in rodents (Figure 3). When bi-steric inhibitor **16** was administered to a rat (iv) or mouse (ip), a major species arising from ring opening of the rapamycin macrocycle was observed, which resulted from the elimination of the β-keto lactone at the C32 carbonyl. In the case of a rat, this seco-species could account for approximately 35% of the parent on an AUC_last_ basis. Other bi-steric compounds, such as **30**, were also susceptible to a similar ring-opening process to yield significant amounts of a seco-species after dosing to rats (data not shown). Comparable seco-species are a well-known degradant from rapamycin itself (where it is known as secorapamycin **34**), which forms in vivo,^91,92^ under enzymatic processes (CYP 3A4)^89,90^ and under basic conditions.^72,74,75^ Testing of the seco-products **32** and **33**, formed from bi-steric inhibitors **16** and **30**, respectively, indicated that they were much less potent as mTORC1 or mTORC2 inhibitors (Table 4).

**Table 4:**
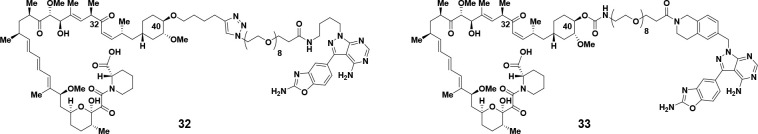
Biological Activity for Seco-Products **32** and **33**

entry	compound	pS6K IC_50_ (nM)	p4EBP1 IC_50_ (nM)	pAKT IC_50_ (nM)
1	**32**	59	180	450
2	**33**	80	320	>1000

**Figure 4:**
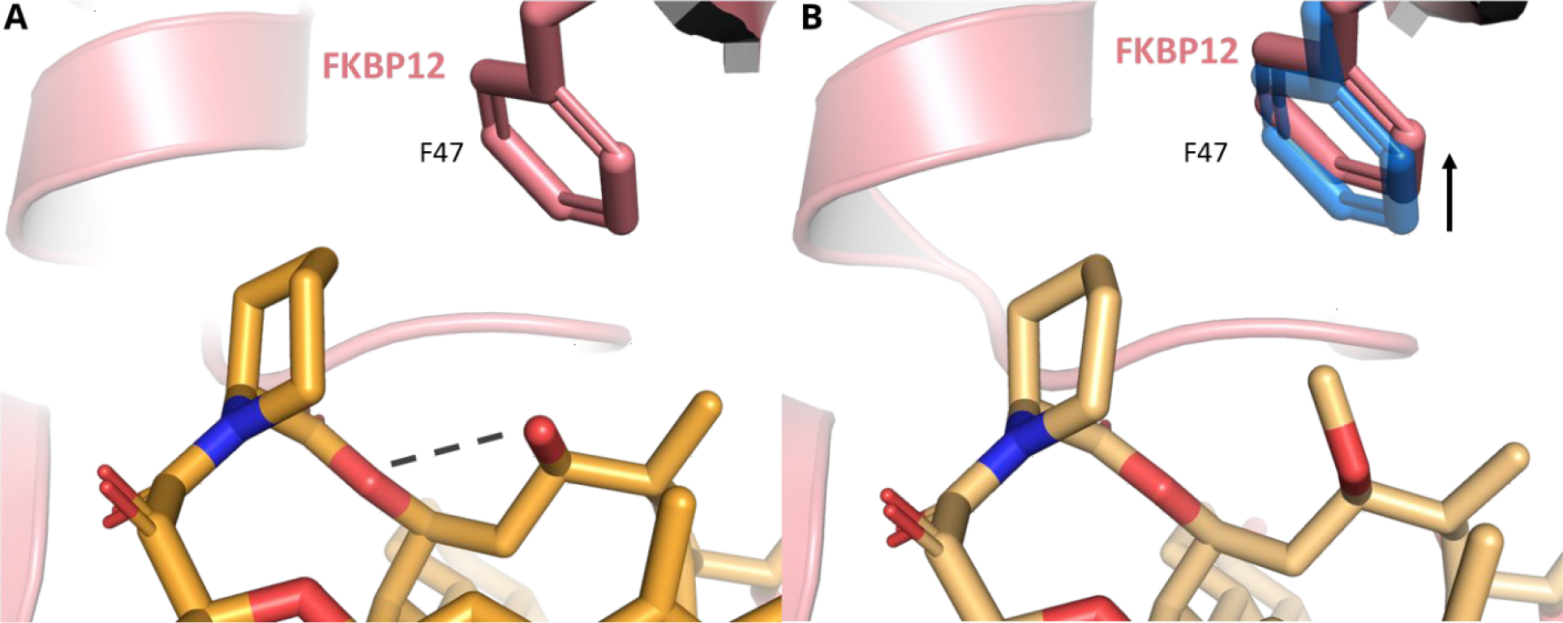
Rapamycin analogs **11** (PDB 8ER6) and **12** (PDB 8ER7) bind at FKBP12’s canonical rapamycin binding site. (A) Hydroxy analog **11** (darker orange) binds in the FKBP12 (red) pocket partially defined by residue F47. C32 hydroxyl residue donates an intramolecular hydrogen bond with the adjacent ester oxygen (dashed line). (B) Methyl ether **12** (lighter orange) is not able to form this intramolecular hydrogen bond, and the methyl group is oriented toward FKBP12 residue F47. As a result, F47 must shift upward to accommodate this additional steric bulk.

Due to the prevalence of a seco-product when bi-steric inhibitors containing a C32 carbonyl were dosed to rodents or when subjected to mildly basic conditions (Scheme 3), we sought to prepare additional compounds that would be less susceptible to forming a ring-opened degradant. To test if modifying the C32 carbonyl of rapamycin was a viable direction, we reduced the carbonyl to a hydroxyl group to give compound **11**(81−83) and also formed the C32 methyl ether to give compound **12** (Scheme 2).^54^ We then measured the affinity of rapamycin **1** together with compounds **11** and **12** for FKBP12 and affinity for FKBP12-FRB (Table 5). In addition, to understand the differences in binding affinities between the rapamycin analogs **11** and **12**, crystal structures of the ternary complex of FKBP12, the FRB domain of mTOR and compounds **11** and **12**, were solved to 2.8 and 3.1 Å, respectively (Figure 4). Elaboration of C32 to the methyl ether results in a ∼1600-fold decrease in affinity for FKBP12 with methyl ether **12** (*K*_d_ = 701 nM) relative to rapamyicin **1** (*K*_d_ = 0.44 nM). The incorporation of this larger group necessitates slight rearrangement of residue F47. However, by replacing the methoxy with a C32 hydroxyl group with compound **11**, the steric bulk was removed and an intramolecular hydrogen bond with the adjacent C34 ester oxygen was formed, which results in a 30-fold decrease in affinity for FKBP12 (*K*_d_ = 13.3 nM) relative to rapamycin **1** (*K*_d_ = 0.44 nM). Despite these differences in FKBP12 *K*_d_ binding, modified rapamycin cores **11** and **12** were potent in a FKBP12-FRB TR-FRET assay (EC_50_ < 10 nM), all being measured at the assay detection limit (Table 5, entries 2 and 3); however, differences in the affinity were observed through pS6K inhibition. The C32 hydroxyl **11** maintained comparable activity with rapamycin **1** (pS6K IC_50_ = 0.04 nM for **11** and 0.06 nM for **1**), whereas the C32 methyl ether resulted in a 6-fold decrease in potency (pS6K IC_50_ = 0.37 nM for **12**).

**Table 5:**
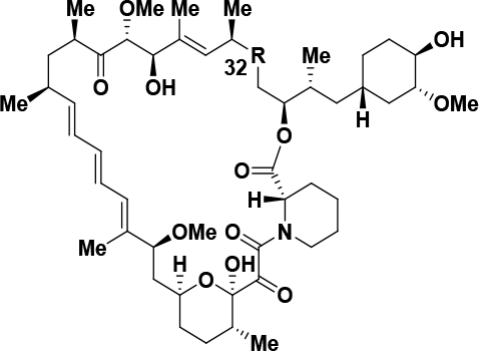
Modification of the Carbonyl at the C32 Position Modulates FKBP12 Binding

entry	compound	R	FKBP12 *K*_d_ (nM)	FKBP12-FRB EC_50_ (nM)	pS6K IC_50_ (nM)	p4EBP1 IC_50_ (nM)	pAKT IC_50_ (nM)
1	**1** (rapamycin)	=O	0.44	<10	0.06	>1000	>1000
2	**11**	(*R*)-OH	13.3	<10	0.04	>1000	>1000
3	**12**	(*R*)-OMe	701	<10	0.37	>1000	>1000

The FKBP12-FRB TR-FRET assay results in Table 5 suggest that bi-steric inhibitors containing a modified C32 position of rapamycin may be a viable alternative to bi-steric inhibitors based on rapamycin itself. Thus, we began to investigate bi-steric compounds in which the C32 carbonyl of rapamycin was reduced to an alcohol or replaced with a methoxy group (Table 6). Accordingly, compounds **35**–**40** were prepared and displayed potent on-target activity for mTORC1 together with a high degree of selectivity over mTORC2. For example, the C32-methoxy rapamycin modification in combination with an active-site inhibitor based on MLN, to give bi-steric inhibitor **35** (Table 6, entry 1), displayed an mTORC1 p4EBP1 inhibition IC_50_ of 1.3 nM and an approximate 10-fold selectivity over mTORC2, as measured by pAKT inhibition (IC_50_ = 13 nM). This compares favorably with its rapamycin counterpart (C32 carbonyl group), bi-steric compound **29** (Table 3, entry 1; p4EBP1 IC_50_ = 1.7 nM; pAKT IC_50_ = 3.6 nM; mTORC1/mTORC2 selectivity ca. 2.1). Similarly, C32-methoxy rapamycin bi-steric inhibitors **37** and **39** (Table 6, entries 3 and 5), which, respectively, utilize a “rigid-MLN” and a modified version of XL388 as the active-site inhibitor, were effective mTORC1 inhibitors with p4EBP1 IC_50_ = 1.6 nM for bi-steric inhibitor **37** and p4EBP1 IC_50_ = 0.97 nM for bi-steric inhibitor **39**, showing much reduced levels of pAKT inhibition (pAKT IC_50_ = 35 nM for **37**, entry 3; pAKT IC_50_ = 35 nM for **39**, entry 5), thus affording an impressive level of mTORC1/mTORC2 selectivity of ca. 21.9 for bi-steric inhibitor **37** and ca. 36.1 for bi-steric inhibitor **39**. We also explored the incorporation of the C32-hydroxy rapamycin core in bi-steric inhibitors, as C32-hydroxy rapamycin had improved binding to FKBP12 compared with the C32-methoxy modification of rapamycin (Table 5). Combination of the C32-hydroxy rapamycin core with an active-site inhibitor based on MLN afforded bi-steric inhibitor **36** (Table 6, entry 2), which had a p4EBP1 inhibition IC_50_ of 1.4 nM and an approximate 6-fold selectivity for mTORC1 over mTORC2. The C32-hydroxy rapamycin core was also combined with the “rigid-MLN” active-site ligand to give bi-steric inhibitor RMC-5552 **38** (entry 4). RMC-5552 **38** showed very potent p4EBP1 inhibition (IC_50_ = 0.48 nM) with much lower pAKT inhibition (IC_50_ = 19 nM), resulting in mTORC1/mTORC2 selectivity approaching 40-fold. Finally, combination of the C32-hydroxy rapamycin core with a modified XL388 active-site inhibitor afforded another impressive bi-steric inhibitor compound RMC-6272 **40** (entry 6), which had a p4EBP1 inhibition IC_50_ of 0.44 nM and significantly lower pAKT inhibition IC_50_ of 12 nM, thus displaying an approximate 27-fold selectivity for mTORC1 over mTORC2.

**Table 6:**
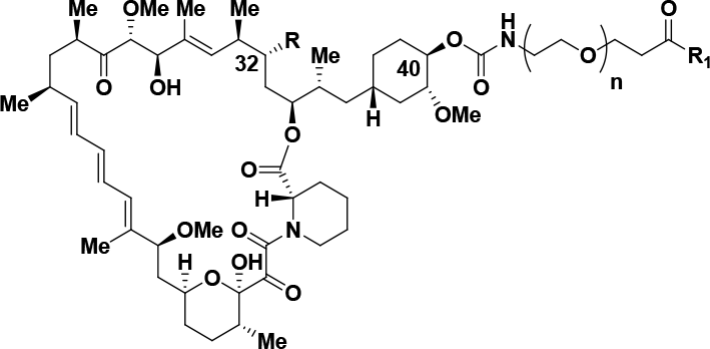
SAR of C32 Modifications and Active-Site Inhibitors

entry	compound no.	PEG repeats	R	active-site inhibitor (R_1_)	pS6K IC_50_ (nM)	p4EBP1 IC_50_ (nM)	pAKT IC_50_ (nM)	selectivity
1	**35**	8	OMe	MLN	0.24	1.3	13	10.0
2	**36**	8	OH		0.49	1.4	8.4	6.0
3	**37**	8	OMe	“rigid MLN”	0.17	1.6	35	21.9
4	**38** (RMC-5552)	8	OH		0.14	0.48	19	39.6
5	**39**	8	OMe	XL388	0.19	0.97	35	36.1
6	**40** (RMC-6272)	8	OH		0.14	0.44	12	27.3

**Figure 5:**
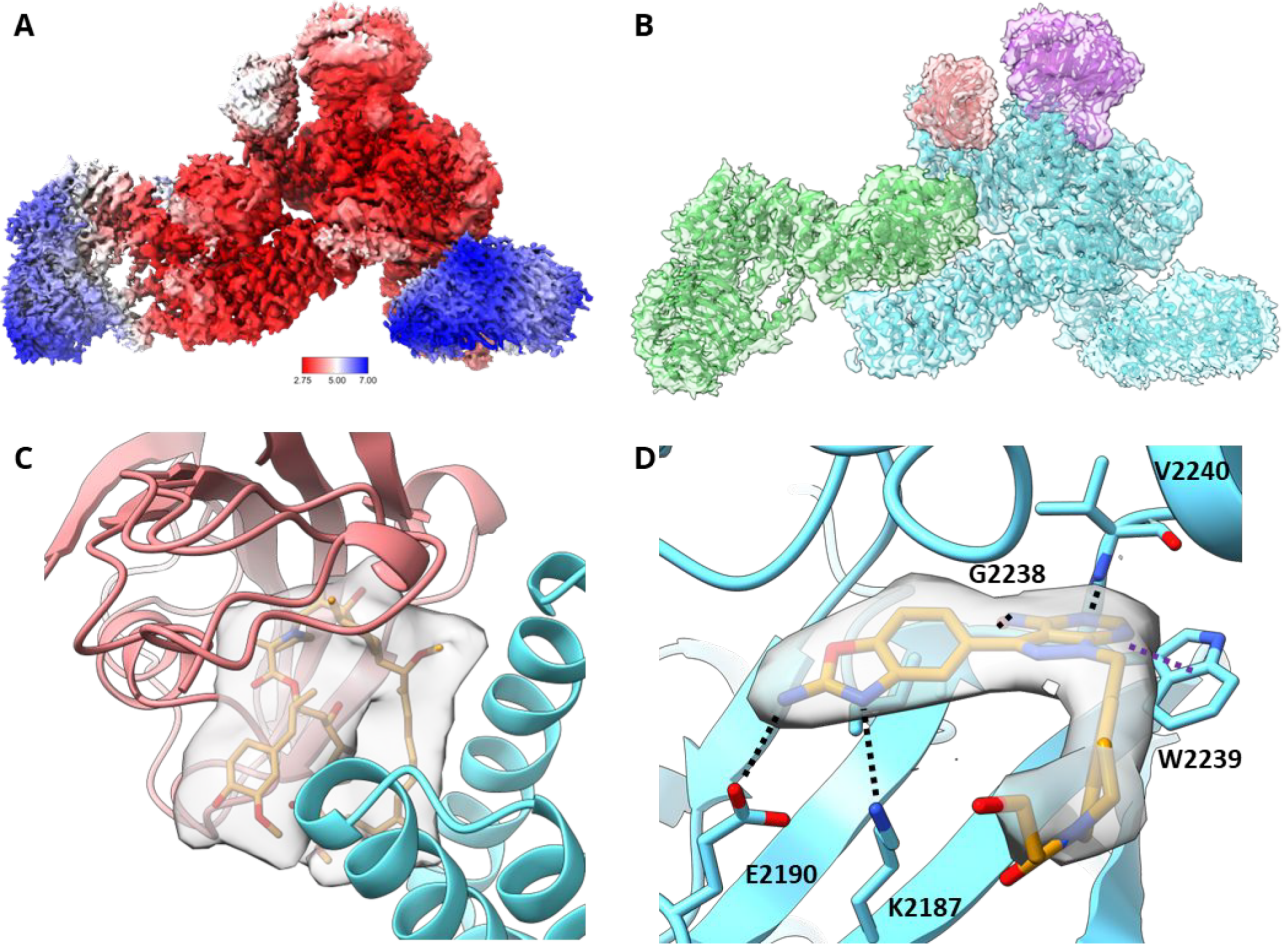
(A) Local resolution map of the mTORC1-RMC-5552-FKBP12 cryo-EM structure (PDB 8ERA). Higher resolution core of the map allows for confident modeling of side chains and both the rapalog-like and the ATP-competitive moieties of RMC-5552. N-HEAT domain of mTOR (right) and WD40 domain of Raptor (left) are more disordered than other regions of the map and consequently approach 7 Å. (B) Cryo-EM map of mTORC1-FKBP12-RMC-5552 **38** complex. mTORC1-FKBP12-RMC-5552 **38** complex cryo-EM map is shown colored by protomer. Blue is mTOR, green is Raptor, purple is mLST8, and red is FKBP12. (C) Cryo-EM map reveals density for rapalog moiety of RMC-5552 **38** binding at its expected location between FKBP12 and the FRB domain at threshold 0.0033. (D) Cryo-EM map reveals density corresponding to the ATP-competitive moiety of RMC-5552 **38** (orange) binding mTOR (blue) at its orthosteric site at threshold 0.024. Multiple hydrogen bonds are formed between mTOR and ligand, including with mTOR residues K2187, E2190, G2238, and V2240 (black dashed lines). 4-Aminopyrazolo[3,4-*d*]pyrimidine core of RMC-5552 π stacks with the aromatic side chain of mTOR residue W2239 (purple dashed line). Density (gray transparent surface) does not extend beyond the beginning of the linker region.

We employed cryogenic electron microscopy (cryo-EM) to determine the structure of the mTORC1-RMC-5552-FKBP12 complex. Three-dimensional (3D) reconstruction of about 800 000 particles yielded a map of 3.1 Å with clear secondary, tertiary, and quaternary structure elements and fit to previously obtained cryo-EM models.^87^ The mTORC1-RMC-5552-FKBP12 particles displayed 2-fold symmetry; therefore, each particle was split into its two monomers giving ∼1 600 000 particles for further 3D refinement. After postprocessing, a final monomeric map of 2.9 Å was used for modeling (Figure 5A). The density observed for mTOR, Raptor, and mLST8 and the overall structure of these elements is very similar to known structures. The maps also demonstrate the presence of FKBP12, whose recruitment would only be observed in the presence of the FKBP12-FRB allosteric modality of RMC-5552 **38** (Figure 5B). In addition, density for RMC-5552 **38** is evident at the interface between FKBP12 and the FRB domain of mTOR (Figure 5C). FKBP12 and the macrocycle interact with the FRB domain of mTOR with the same binding mode and in a similar orientation as observed in the crystal structures described in Figure 4. The ATP-competitive, orthosteric site also shows unambiguous density in the map. RMC-5552 **38** makes hydrogen bonds to the backbone of G2238 and V2240, the “hinge” of mTOR, via the 4-aminopyrazolo[3,4-*d*]pyrimidine core, and the 2-aminobenzoxazole makes hydrogen-bonding interactions to E2190 and K2187 (Figure 5D). The strong densities at both the ATP-competitive and the allosteric sites suggest that both sites are simultaneously occupied by RMC-5552 **38** in the sample. Unsurprisingly, no density is observed for the flexible PEG linker, and it is not modeled.

ADME/PK parameters for compounds RMC-5552 **38** and RMC-6272 **40** in Balb/c mice after a single intraperitoneal (ip) injection are shown in Table 7. RMC-5552 **38** showed a high *C*_max_ (3.19 ± 0.62 μM) and high exposure (AUC_last_ = 25.9 ± 3.0 μM × h), providing plasma concentrations several multiples above the cellular IC_50_ for p4EBP1 inhibition (IC_50_ = 0.48 nM) for an extended period. Compound RMC-6272 **40** also demonstrated an attractive pharmacokinetic profile with extended plasma concentrations above the IC_50_ for p4EBP1 inhibition (IC_50_ = 0.44 nM), although *C*_max_ (0.97 μM ± 0.10 μM) and overall exposure (AUC_last_ = 8.7 ± 1.2 μM × h) were somewhat lower than those for RMC-5552 **38**.

**Table 7:** PK Parameters of RMC-5552 **38** and RMC-6272 **40** in Mice at 1 mg/kg via IP Administration^a^

compounds	*T*_max_ (h)	*C*_max_ (ng/mL)	*C*_max_ (μM)	AUC_last_ (ng/mL × h)	AUC_last_ (μM × h)	*t*_1/2_ (h)
**38** RMC-5552	2.0 ± 0.0	5667 ± 1106	3.19 ± 0.62	46 089 ± 5320	25.9 ± 3.0	4.8 ± 0.4
**40** RMC-6272	2.3 ± 1.5	1793 ± 186	0.97 ± 0.10	16 169 ± 2293	8.7 ± 1.2	3.8 ± 0.6

a Male Balb/c mice (mean ± SD, *n* = 3); vehicle = transcutol/solutol HS15/H_2_O 5%/5%/90% (v,w,v)

**Figure 6:**
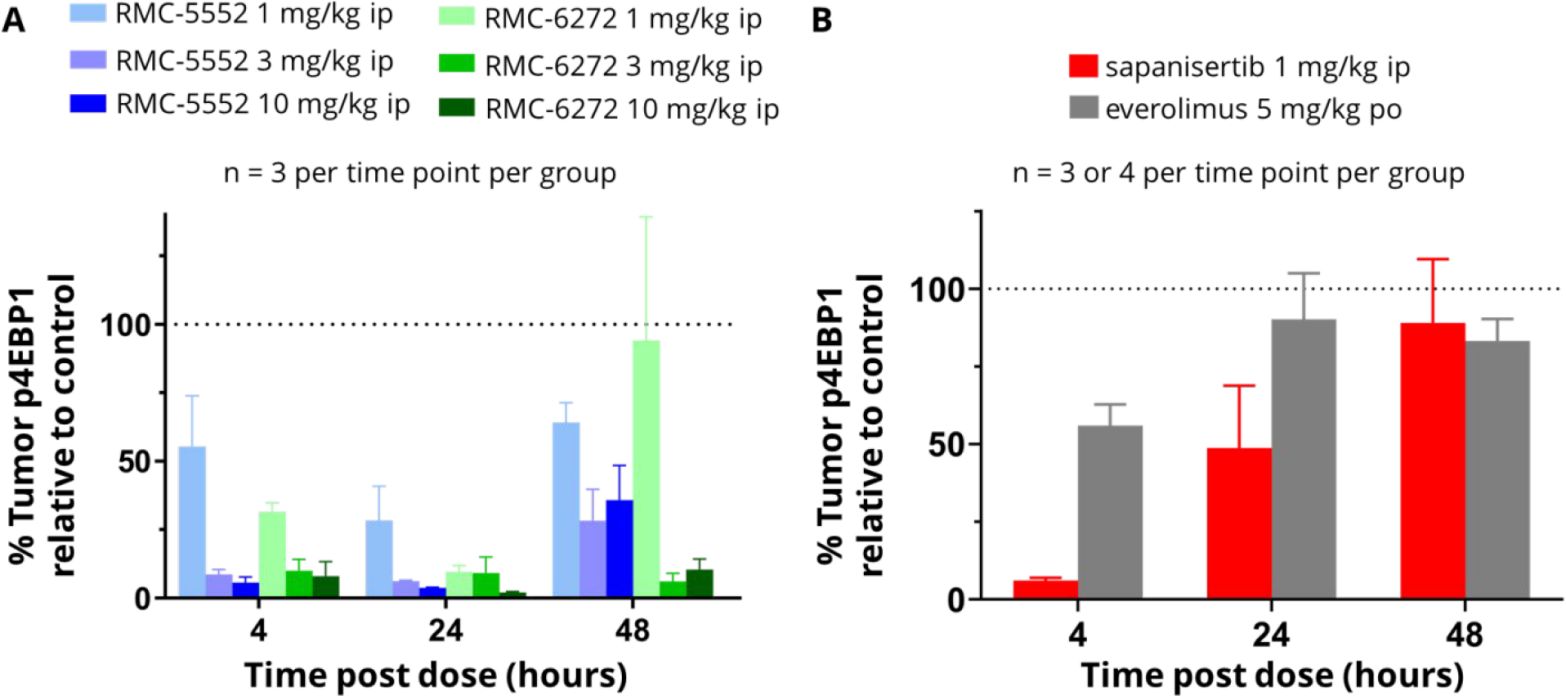
Tumor PD after a single dose of each inhibitor in the MCF-7 CDX model: (A) RMC-5552 **38** and RMC-6272 **40**; (B) sapanisertib **5** and everolimus **4**.

Overall, our SAR studies with the active-site inhibitor, linker composition, linker length, and chemical handle of attachment demonstrated that modifications to bi-steric inhibitors can afford new analogs with higher inhibitory potency for mTORC1 and increased mTORC1/mTORC2 selectivity. By reducing the C32 ketone on the rapamycin core, the affinity for FKBP12 and the FKBP12-FRB complexes could be modulated, and the chemical stability of bi-steric analogs could also be improved. Taken together with preliminary pharmacokinetic data, our work provided a number of potent and selective bi-steric inhibitors which were suitable for advancement to antitumor activity studies in vivo. We then examined representative compounds in pharmacokinetic–pharmacodynamic (PK–PD) studies using human cancer cell line-derived xenograft (CDX) tumor models in mice. The leading bi-steric inhibitor RMC-5552 **38** (p4EBP1 IC_50_ = 0.48 nM; mTORC1/mTORC2 ca. 40) was studied in a CDX model using the Michigan Cancer Foundation-7 (MCF-7) breast cancer cell line, which bears an activating mutation in the p110α catalytic subunit of Phosphoinositide 3-kinase (PIK3CA^E545K^). Administration of a single intraperitoneal (ip) dose of RMC-5552 **38** resulted in a dose-dependent, prolonged, and profound inhibition of tumor p4EBP1 levels up to 48 h (blue bars in Figure 6A). Separately, bi-steric inhibitor RMC-6272 **40** also showed dose-dependent, robust, and long-lasting inhibition of tumor p4EBP1 levels when dosed via intraperitoneal injection (green bars in Figure 6A). In comparison, everolimus **4** treatment showed a minimal effect upon p4EBP1 levels after oral administration of a single dose at 5 mg/kg (gray bars in Figure 6B). This result agrees with in vitro data showing that everolimus **4** is not a potent inhibitor of p4EBP1 (p4EBP1 IC_50_ > 1000 nM). The dual mTORC1/mTORC2 active-site inhibitor sapanisertib **5**, which is moderately potent for inhibition of 4EBP1 phosphorylation in vitro (p4EBP1 IC_50_ = 19 nM), caused significant (>80% inhibition) inhibition of tumor p4EBP1 at 4 h after intraperitoneal dosing at 1 mg/kg and reduced inhibition after 24 h, consistent with the elimination kinetics of this compound (red bars in Figure 6B).

**Figure 7:**
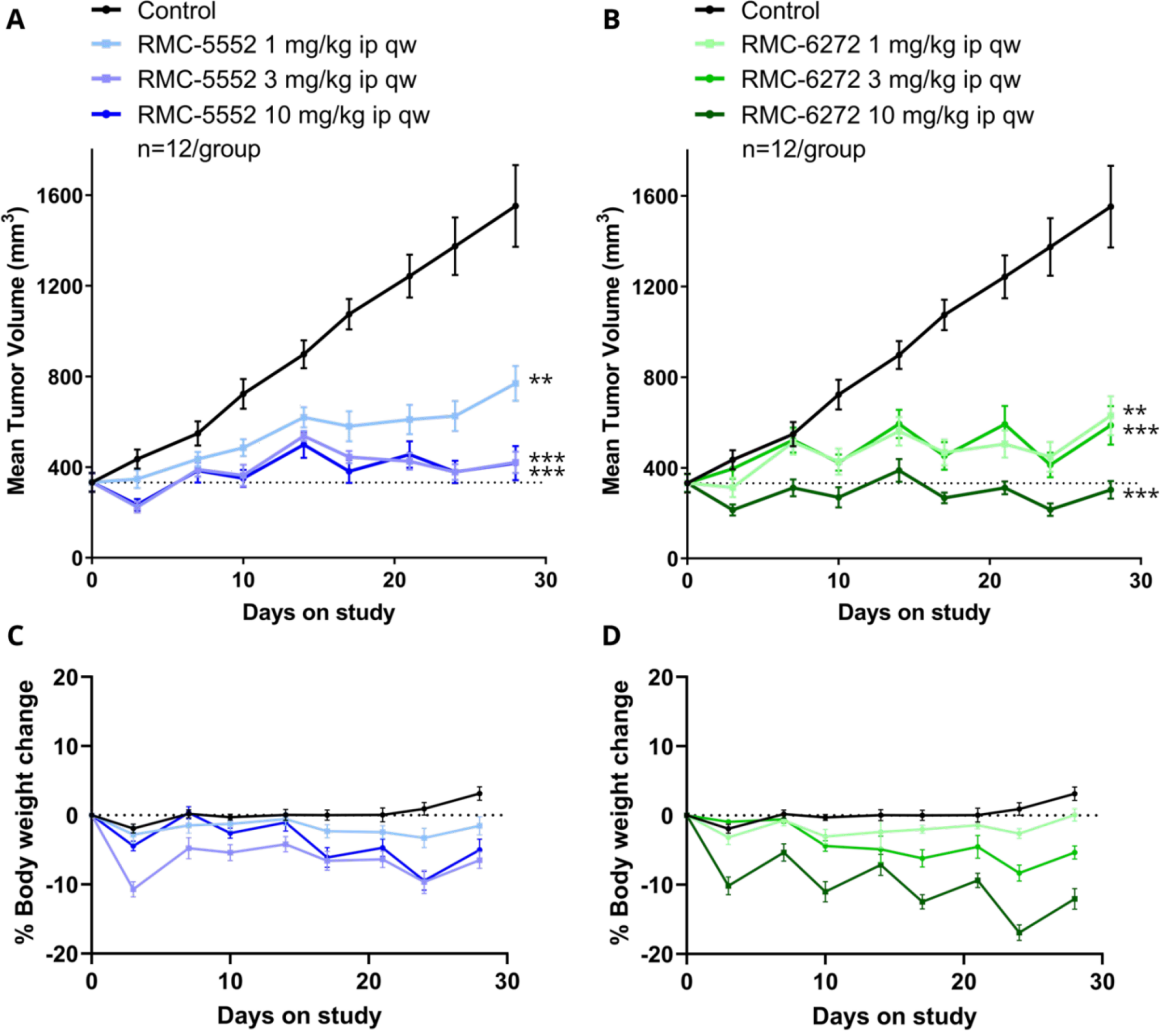
Mean tumor volume over time in MCF-7 CDX model for (A) RMC-5552 **38** and (B) RMC-6272 **40**. Data were analyzed by two-way repeated measures ANOVA; ** *p* < 0.01 and *** *p* < 0.001, as compared to control at end of study. Mean percentage body weight change for (C) RMC-5552 **38** and (D) RMC-6272 **40**.

The pharmacodynamic activity observed with RMC-5552 **38** and RMC-6272 **40** translated into antitumor activity upon repeat dosing when each compound was examined in a 28-day study in the MCF-7 (PIK3CA^E545K^) cell line-derived xenograft (CDX) model (Figure 7). RMC-5552 **38** or RMC-6272 **40** treatment resulted in a reduction in tumor volume when dosed once weekly via intraperitoneal injection,^56^ the most significant effects being observed at 3 or 10 mg/kg ip once weekly (Figure 7A and 7B, respectively). RMC-5552 **38** and RMC-6272 **40** both exhibited an acceptable tolerability profile up to 10 mg/kg ip once weekly with modest, cyclical effects on body weight loss (Figure 7C and 7D, respectively).

**Figure 8:**
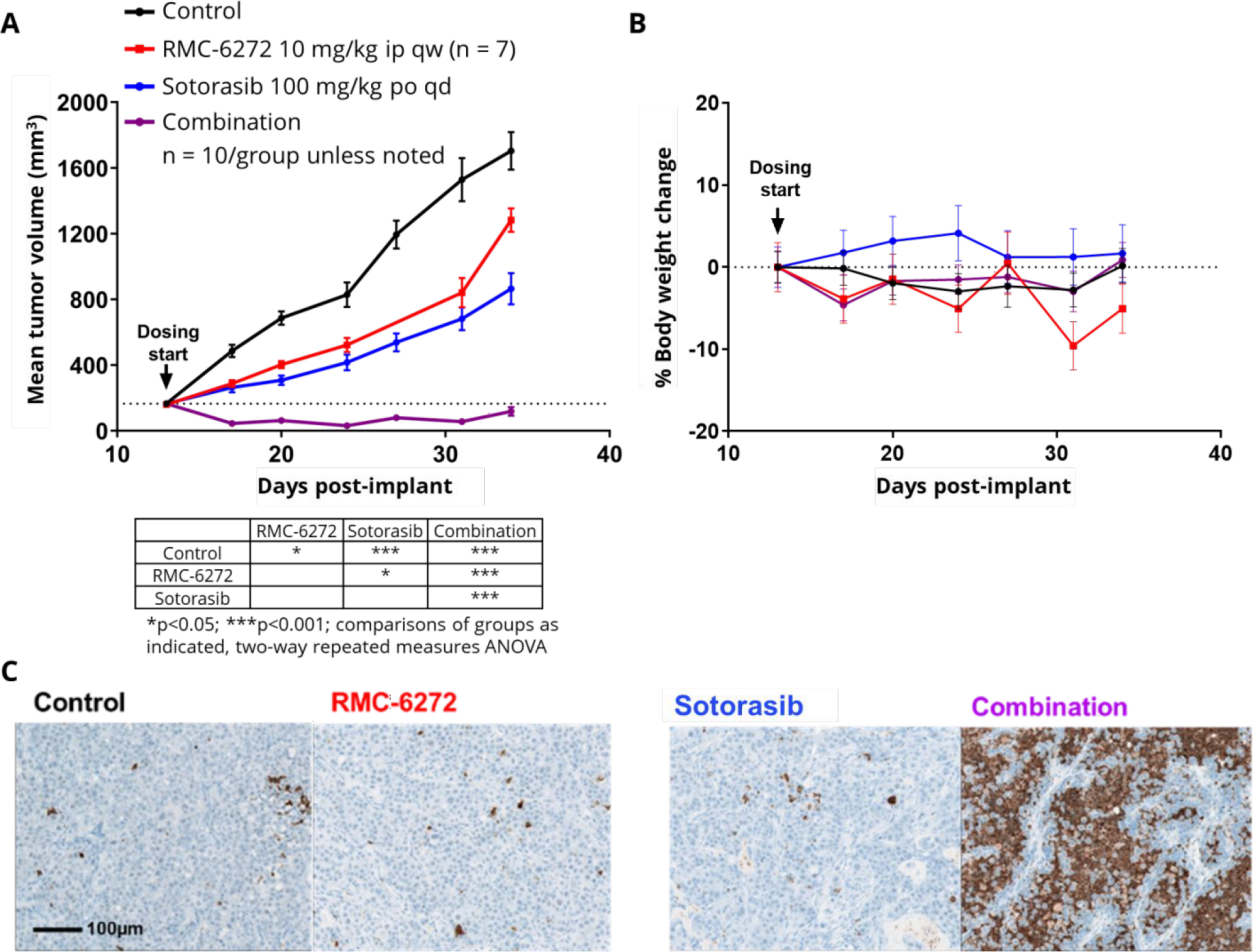
(A) Mean tumor volume over time, (B) mean percentage body weight change, and (C) caspase-3 cleavage staining 24 h post a single dose for RMC-6272 **40**, sotorasib, and the combination in the NCI-H2122 CDX model.

Given the central role of mTOR in cell growth and metabolism through extensive crosstalk with key biological pathways, selective mTORC1 inhibition and downstream reduction in p4EBP1 levels also offer significant opportunities beyond potential use as a monotherapy.^93^ One combination that is particularly appealing is with inhibitors of mutated oncogenic forms of Kirsten rat sarcoma (KRAS), such as KRAS^G12C^.^94−96^ We investigated the combination of RMC-6272 **40** and sotorasib (AMG-510), the first approved KRAS^G12C^ inhibitor for *KRAS*^*G12C*^-mutated locally advanced or metastatic nonsmall cell lung cancer (NSCLC),^97,98^ in the NCI-H2122 NSCLC CDX model. Despite harboring a *KRAS*^*G12C*^ mutation, this model is relatively insensitive to KRAS^G12C^ inhibition.^94,95^ Significantly, NCI-H2122 also harbors a *STK11*^*LOF*^ mutation. The STK11 gene product negatively regulates mTORC1 and functions as a tumor suppressor. Up to 25% of *KRAS*^*G12C*^ NSCLC patients have co-occurring *STK11*^*mut*^ with an estimate of over 5400 new patients per annum in the United States.^62^ This patient population appears refractory to anti-PD-1 treatment and is a significant unmet medical need.^98,99^ Neither RMC-6272 **40** (10 mg/kg ip once weekly) nor sotorasib (100 mg/kg po, qd) induced tumor regressions when dosed as single agents in this model (Figure 8A). However, the combination elicited a robust combinatorial antitumor response, causing tumor regressions. The combination regimen also showed acceptable tolerability as assessed by body weight loss (Figure 8B). In addition, significant induction of apoptosis (in contrast to minimal induction following monotherapy) was observed for the combination regimen of RMC-6272 **40** and sotorasib, consistent with the tumor regressions observed thereof. These preclinical data support the approach of modulating the 4EBP1-eIF4E axis to enhance the clinical effectiveness of KRAS^G12C^ inhibitors.

RMC-5552 **38** and RMC-6272 **40** were next evaluated against a series of off-targets and safety screens. Both compounds were >50-fold selective for mTORC1 over other lipid kinases and exhibited <30% inhibition when screened at 1 μM against a panel of 300 kinases.^100^ Inhibition of the human Ether-à-go-go-Related Gene (hERG) ion channel was low when the compounds were screened at 10 μM. RMC-5552 **38**, our most selective inhibitor with broad tolerability, emerged as a preferred clinical candidate. In addition, when RMC-5552 **38** (10 μM) was profiled in the Eurofins Safety Screen 44, a well-known “cross pharma”-recommended panel against undesirable off-targets,^101^ no significant inhibition was observed.

The profile of compound RMC-5552 **38** and compound RMC-6272 **40** is detailed in Table 8.

**Table 8:**
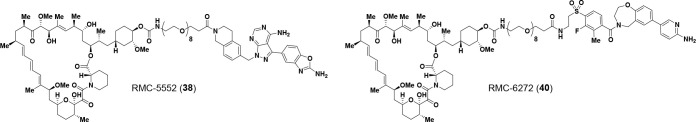
Preclinical Profile of RMC-5552 **38** and RMC-6272 **40**

key data summary	RMC-5552 **38**	RMC-6272 **40**
molecular weight (g/mol)	1778.2	1850.3
MDA-MB-468 cell p4EBP1 IC_50_ (nM)	0.48	0.44
mTORC1/2 selectivity in MDA-MB-486 cell assay	40×	27×
selectivity for mTORC1 over other lipid kinases	>53×	>1000×
cellular kinase panel (∼300) (1 μM)	<30% inhibition	<30% inhibition
MCF-7 tumor PD: dose proportional duration of >80% p4EBP1 inhibition	24–48 h	24–48 h
in vitro hERG inhibition (10 μM)	16%	6%
Eurofins Safety Screen 44 (10 μM)	<5% inhibition (low promiscuity)	

## Conclusion

Herein, we have highlighted the development of two bi-steric inhibitors RMC-5552 **38** and RMC-6272 **40** that potently and selectively inhibit mTORC1-mediated phosphorylation of 4EBP1 and other substrates. These inhibitors resulted from a systematic examination of the active-site inhibitor, linker, and rapamycin core in order to refine on-target mTORC1 activity, selectivity over mTORC2, pharmacokinetic properties, chemical stability, and synthetic tractability. Selective inhibition of mTORC1 could be beneficial for treating cancers with aberrant activation of mTORC1 via mutations in the PI3K/mTOR pathway, and indeed, RMC-5552 **38** and RMC-6272 **40** each exhibit single-agent antitumor activity in a human xenograft model of PIK3CA mutant breast cancer in mice in vivo. Aberrant activation of mTORC1 can also co-occur with mutations in other cancer drivers, such as RAS, limiting the antitumor activity of targeted RAS inhibitors. An mTORC1 inhibitor may have utility as a companion to RAS inhibitors in this context. Consistent with this hypothesis, RMC-6272 **40** induces widespread apoptosis and deep tumor regressions in a xenograft model of *KRAS*^*G12C*^ mutant NSCLC bearing a loss of function mutation in *STK11*, a negative regulator of mTORC1. This model exhibits only modest inhibition of tumor growth in response to single-agent treatment with the KRAS^G12C^ inhibitor sotorasib, suggesting that mTORC1 activation can contribute to resistance to KRAS^G12C^ inhibitors, which can be overcome by combination with an mTORC1 inhibitor. RMC-5552 **38** has now advanced to clinical studies for further evaluation.^71^

## Experimental Section

### Compound Synthesis and Characterization

All solvents and commercially available reagents were used as received. All reactions were followed by TLC analysis or LCMS. Column chromatography was performed on prepacked silica gel columns (Biotage SNAP KP-Sil) using a Biotage Isolera One system. Reverse-phase preparative chromatography was performed on a Uptisphere Strategy C18-HQ 5 μm 150 mm × 7 mm column using an Interchim PuriFlash system. The column was eluted with MeCN/H_2_O with 0.1% formic acid. All key compounds were >95% purity by HPLC. The purity for compounds and low-resolution mass spectra were determined using liquid chromatography mass spectrometry (LCMS) on a Shimadzu LC-20 instrument using electrospray ionization. LCMS conditions were as follows: Uptisphere Strategy C18-HQ 5 μm 150 × 4.6 mm, 55% → 100% MeCN (0.1% TFA) in H_2_O (0.1% TFA), 20 min run, oven temperature 60 °C, flow rate 0.5 mL/min, UV detection (λ = 280 nm). HRMS were performed on a Thermo Fisher LTQ Orbitrap using high-performance liquid chromatography with electrospray ionization Orbitrap mass spectrometry (HPLC ESI Orbitrap/MS). Liquid-state ^1^H NMR experiments for intermediates were recorded on 400, 500, or 600 MHz Bruker Avance III NMR spectrometers. Liquid-state ^1^H, COSY, ^13^C, and HMBC spectra were recorded on a 600 MHz Bruker Avance III NMR spectrometer (600 MHz for ^1^H, 151 MHz for ^13^C) using a triple-resonance ^1^H,^15^N,^13^C CP-TCI 5 mm cryoprobe (Bruker Biospin, Germany). All of the experiments used for the resonance assignment procedure and the elucidation of the final products structures (1D ^1^H, 1D ^13^C, 2D ^1^H–^1^H-COSY, 2D ^1^H–^1^H-TOCSY, 2D ^1^H–^1^H-ROESY, 2D ^1^H–^13^C-HSQC, 2D ^1^H–^13^C-HMBC) were recorded at 300 K. ^1^H chemical shifts are reported in δ (ppm) as s (singlet), d (doublet), t (triplet), q (quartet), dd (double doublet), m (multiplet), br s (broad singlet), and o (overlay) and are referenced to TMS as an internal standard.

#### *N*-(4-(4-Amino-3-(2-aminobenzo[*d*]oxazol-5-yl)-1*H*-pyrazolo[3,4-*d*]pyrimidin-1-yl)butyl)-1-(4-(4-(((1*R*,2*R*,4*S*)-4-((*R*)-2-((3*S*,6*R*,7*E*,9*R*,10*R*,12*R*,14*S*,15*E*,17*E*,19*E*,21*S*,23*S*,26*R*,27*R*,34a*S*)-9,27-dihydroxy-10,21-dimethoxy-6,8,12,14,20,26-hexamethyl-1,5,11,28,29-pentaoxo-1,4,5,6,9,10,11,12,13,14,21,22,23,24,25,26,27,28,29,31,32,33,34,34a-tetracosahydro-3*H*-23,27-epoxypyrido[2,1-*c*][1]oxa[4]azacyclohentriacontin-3-yl)propyl)-2-methoxycyclohexyl)oxy)butyl)-1*H*-1,2,3-triazol-1-yl)-3,6,9,12,15,18,21,24-octaoxaheptacosan-27-amide (**16**)

To a solution of **10** (207 mg, 208 μmol, 1.0 equiv) and **SI-7** (281.2 mg, 357 μmol, 1.7 equiv) in DMSO (5.2 mL) was added Cu(MeCN)_4_PF_6_ (155 mg, 416 μmol, 2.0 equiv) followed by TBTA (444 mg, 837 μmol, 4.0 equiv). The reaction mixture was then stirred at room temperature for 5 h. Purification of the reaction mixture by reverse-phase chromatography (40% → 90% MeCN/H_2_O) afforded the desired product (109.1 mg, 30% yield) as a colorless amorphous solid. LCMS (ESI) *m*/*z*: [M+H] calcd for C_92_H_140_N_12_O_23_ 1782.02; found 1782.2.

#### *N*-(4-(4-Amino-3-(5-hydroxy-1*H*-indol-2-yl)-1*H*-pyrazolo[3,4-*d*]pyrimidin-1-yl)butyl)-1-(4-(4-(((1*R*,2*R*,4*S*)-4-((*R*)-2-((3*S*,6*R*,7*E*,9*R*,10*R*,12*R*,14*S*,15*E*,17*E*,19*E*,21*S*,23*S*,26*R*,27*R*,34a*S*)-9,27-dihydroxy-10,21-ddimethoxy-6,8,12,14,20,26-hexamethyl-1,5,11,28,29-pentaoxo-1,4,5,6,9,10,11,12,13,14,21,22,23,24,25,26,27,28,29,31,32,33,34,34a-tetracosahydro-3*H*-23,27-epoxypyrido[2,1-*c*][1]oxa[4]azacyclohentriacontin-3-yl)propyl)-2-methoxycyclohexyl)oxy)butyl)-1*H*-1,2,3-triazol-1-yl)-3,6,9,12,15,18,21,24-octaoxaheptacosan-27-amide (**18**)

To a solution of **10** (0.45 g, 0.45 mmol, 1.0 equiv) and **SI-13** (623 mg, 0.792 mmol, 1.8 equiv) in DMSO (9.1 mL) was added Cu(MeCN)_4_PF_6_ (337 mg, 0.904 mmol, 2.0 equiv) followed by TBTA (960 mg, 1.8 mmol, 4.0 equiv). The reaction mixture was then stirred at room temperature for 6 h. Purification of the reaction mixture by reverse-phase chromatography (40% → 90% MeCN/H_2_O) afforded the desired product (164 mg, 20% yield) as a colorless amorphous solid. LCMS (ESI) *m*/*z*: [M+H] calcd for C_93_H_141_N_11_O_23_ 1781.02; found 1781.2

#### (3*S*,6*R*,7*E*,9*R*,10*R*,12*R*,14*S*,15*E*,17*E*,19*E*,21*S*,23*S*,26*R*,27*R*,34a*S*)-9,27-Dihydroxy-10,21-ddimethoxy-3-((*R*)-1-((1*S*,3*R*,4*R*)-3-methoxy-4-(4-(1-(27-(4-(4-(8-(6-methoxypyridin-3-yl)-3-methyl-2-oxo-2,3-dihydro-1*H*-imidazo[4,5-*c*]quinolin-1-yl)-2-(trifluoromethyl)phenyl)piperazin-1-yl)-27-oxo-3,6,9,12,15,18,21,24-octaoxaheptacosyl)-1*H*-1,2,3-triazol-4-yl)butoxy)cyclohexyl)propan-2-yl)-6,8,12,14,20,26-hexamethyl-9,10,12,13,14,21,22,23,24,25,26,27,32,33,34,34a-hexadecahydro-3*H*-23,27-epoxypyrido[2,1-*c*][1]oxa[4]azacyclohentriacontine-1,5,11,28,29(4*H*,6*H*,31*H*)-pentaone (**19**)

To a solution of **10** (0.40 g, 0.402 mmol, 1.0 equiv) and **SI-15** (791 mg, 0.804 mmol, 2.0 equiv) in DMSO (8 mL) was added Cu(MeCN)_4_PF_6_ (299 mg, 0.804 mmol, 2.0 equiv) followed by TBTA (848 mg, 1.6 mmol, 4.0 equiv). The reaction mixture was then stirred at room temperature for 6 h. Purification of the reaction mixture by reverse-phase chromatography (40% → 90% MeCN/H_2_O) afforded the desired product (279 mg, 35% yield) as a colorless amorphous solid. LCMS (ESI) *m*/*z*: [M+H] calcd for C_104_H_147_F_3_N_10_O_24_ 1978.06; found 1977.9.

#### *N*-(4-(4-Amino-3-(1*H*-pyrrolo[2,3-*b*]pyridin-5-yl)-1*H*-pyrazolo[3,4-*d*]pyrimidin-1-yl)butyl)-1-(4-(4-(((1*R*,2*R*,4*S*)-4-((*R*)-2-((3*S*,6*R*,7*E*,9*R*,10*R*,12*R*,14*S*,15*E*,17*E*,19*E*,21*S*,23*S*,26*R*,27*R*,34a*S*)-9,27-dihydroxy-10,21-ddimethoxy-6,8,12,14,20,26-hexamethyl-1,5,11,28,29-pentaoxo-1,4,5,6,9,10,11,12,13,14,21,22,23,24,25,26,27,28,29,31,32,33,34,34a-tetracosahydro-3*H*-23,27-epoxypyrido[2,1-*c*][1]oxa[4]azacyclohentriacontin-3-yl)propyl)-2-methoxycyclohexyl)oxy)butyl)-1*H*-1,2,3-triazol-1-yl)-3,6,9,12,15,18,21,24-octaoxaheptacosan-27-amide (**20**)

To a solution of **10** (400 mg, 0.402 mmol, 1.0 equiv) and **SI-19** (620 mg, 0.804 mmol, 2.0 equiv) in DMSO (8 mL) was added Cu(MeCN)_4_PF_6_ (299 mg, 0.804 mmol, 2.0 equiv) followed by TBTA (848 mg, 1.6 mmol, 4.0 equiv). The reaction mixture was then stirred at room temperature for 6 h. Purification of the reaction mixture by reverse-phase chromatography (40% → 90% MeCN/H_2_O) afforded the desired product (298 mg, 42% yield) as a colorless amorphous solid. LCMS (ESI) *m*/*z*: [M+H] calcd for C_92_H_140_N_12_O_22_ 1766.03; found 1765.9.

#### (3*S*,6*R*,7*E*,9*R*,10*R*,12*R*,14*S*,15*E*,17*E*,19*E*,21*S*,23*S*,26*R*,27*R*,34a*S*)-3-((*R*)-1-((1*S*,3*R*,4*R*)-4-(4-(1-(27-(6-((4-Amino-3-(2-aminobenzo[*d*]oxazol-5-yl)-1*H*-pyrazolo[3,4-*d*]pyrimidin-1-yl)methyl)-3,4-dihydroisoquinolin-2(1*H*)-yl)-27-oxo-3,6,9,12,15,18,21,24-octaoxaheptacosyl)-1*H*-1,2,3-triazol-4-yl)butoxy)-3-methoxycyclohexyl)propan-2-yl)-9,27-dihydroxy-10,21-ddimethoxy-6,8,12,14,20,26-hexamethyl-9,10,12,13,14,21,22,23,24,25,26,27,32,33,34,34a-hexadecahydro-3*H*-23,27-epoxypyrido[2,1-*c*][1]oxa[4]azacyclohentriacontine-1,5,11,28,29(4*H*,6*H*,31*H*)-pentaone (**21**)

To a solution of **10** (400 mg, 402.2 μmol, 1.0 equiv) and **SI-24** (589 mg, 683.7 μmol, 1.7 equiv) in DMSO (8 mL) was added Cu(MeCN)_4_PF_6_ (299 mg, 804.4 μmol, 2.0 equiv) followed by TBTA (848 mg, 1.6 mmol, 4.0 equiv). The reaction mixture was then stirred at room temperature for 6 h. Purification of the reaction mixture by reverse-phase chromatography (40% → 90% MeCN/H_2_O) afforded the desired product (424 mg, 57% yield) as a colorless amorphous solid. LCMS (ESI) *m*/*z*: [M+H] calcd for C_98_H_142_N_12_O_23_ 1856.04; found 1856.0.

#### (3*S*,6*R*,7*E*,9*R*,10*R*,12*R*,14S,15*E*,17*E*,19*E*,21*S*,23*S*,26*R*,27*R*,34a*S*)-3-((*R*)-1-((1*S*,3*R*,4*R*)-4-(4-(1-(27-(4-((4-Amino-3-(2-aminobenzo[*d*]oxazol-5-yl)-1*H*-pyrazolo[3,4-*d*]pyrimidin-1-yl)methyl)piperidin-1-yl)-27-oxo-3,6,9,12,15,18,21,24-octaoxaheptacosyl)-1*H*-1,2,3-triazol-4-yl)butoxy)-3-methoxycyclohexyl)propan-2-yl)-9,27-dihydroxy-10,21-ddimethoxy-6,8,12,14,20,26-hexamethyl-9,10,12,13,14,21,22,23,24,25,26,27,32,33,34,34a-hexadecahydro-3*H*-23,27-epoxypyrido[2,1-*c*][1]oxa[4]azacyclohentriacontine-1,5,11,28,29(4*H*,6*H*,31*H*)-pentaone (**22**)

To a solution of **10** (20 mg, 20.1 μmol, 1.0 equiv) and **SI-28** (32.7 mg, 40.2 μmol, 2.0 equiv) in DMSO (2 mL) was added Cu(MeCN)_4_PF_6_ (14.9 mg, 40.2 μmol, 2.0 equiv) followed by TBTA (42.6 mg, 80.4 μmol, 4.0 equiv). The reaction mixture was then stirred at room temperature for 3 h. Purification of the reaction mixture by reverse-phase chromatography (40% → 90% MeCN/H_2_O) afforded the desired product (22.5 mg, 62% yield) as a colorless amorphous solid. LCMS (ESI) *m*/*z*: [M+H] calcd for C_94_H_142_N_12_O_23_ 1808.04; found 1808.1.

#### *N*-(4-(3-(2-Aminobenzo[*d*]oxazol-5-yl)-4-(dimethylamino)-1*H*-pyrazolo[3,4-*d*]pyrimidin-1-yl)butyl)-1-(4-(4-(((1*R*,2*R*,4*S*)-4-((*R*)-2-((3*S*,6*R*,7*E*,9*R*,10*R*,12*R*,14*S*,15*E*,17*E*,19*E*,21*S*,23*S*,26*R*,27*R*,34a*S*)-9,27-dihydroxy-10,21-ddimethoxy-6,8,12,14,20,26-hexamethyl-1,5,11,28,29-pentaoxo-1,4,5,6,9,10,11,12,13,14,21,22,23,24,25,26,27,28,29,31,32,33,34,34a-tetracosahydro-3*H*-23,27-epoxypyrido[2,1-*c*][1]oxa[4]azacyclohentriacontin-3-yl)propyl)-2-methoxycyclohexyl)oxy)butyl)-1*H*-1,2,3-triazol-1-yl)-3,6,10,13,16,19,22,25-octaoxaoctacosan-28-amide (**23**)

To a solution of **10** (150 mg, 151 μmol, 1.0 equiv) and **SI-32** (209 mg, 256.3 μmol, 1.7 equiv) in DMSO (3 mL) was added Cu(MeCN)_4_PF_6_ (112 mg, 302 μmol, 2.0 equiv) followed by TBTA (320 mg, 603 μmol, 4.0 equiv). The reaction mixture was then stirred at room temperature for 4 h. Purification of the reaction mixture by reverse-phase chromatography (40% → 90% MeCN/H_2_O) afforded the desired product (153 mg, 56% yield) as a colorless amorphous solid. LCMS (ESI) *m*/*z*: [M+H] calcd for C_94_H_144_N_12_O_23_ 1810.05; found 1810.0.

#### *N*-(4-(4-Amino-3-(1*H*-pyrrolo[2,3-*b*]pyridin-5-yl)-1*H*-pyrazolo[3,4-*d*]pyrimidin-1-yl)butyl)-1-(4-(4-(((1*R*,2*R*,4*S*)-4-((*R*)-2-((3*S*,6*R*,7*E*,9*R*,10*R*,12*R*,14*S*,15*E*,17*E*,19*E*,21*S*,23*S*,26*R*,27*R*,34a*S*)-9,27-dihydroxy-10,21-ddimethoxy-6,8,12,14,20,26-hexamethyl-1,5,11,28,29-pentaoxo-1,4,5,6,9,10,11,12,13,14,21,22,23,24,25,26,27,28,29,31,32,33,34,34a-tetracosahydro-3*H*-23,27-epoxypyrido[2,1-*c*][1]oxa[4]azacyclohentriacontin-3-yl)propyl)-2-methoxycyclohexyl)oxy)butyl)-1*H*-1,2,3-triazol-1-yl)-3,6,9,12,15,18-hexaoxahenicosan-21-amide (**24**)

To a solution of **10** (50 mg, 50.2 μmol, 1.0 equiv) and **SI-20** (51.5 mg, 75.2 μmol, 1.5 equiv) in MeOH (10 mL) was added a 1 M solution of CuSO_4_ in H_2_O (0.186 mL, 186 μmol, 3.7 equiv) followed by a 1 M solution of sodium ascorbate in H_2_O (0.251 mL, 251 μmol, 5 equiv). After stirring overnight, a 1 M solution of CuSO_4_ in H_2_O (185 μmol, 3.7 equiv) followed by a 1 M solution of sodium ascorbate in H_2_O (251 μmol, 5 equiv) was added. After stirring overnight, the reaction mixture was concentrated under reduced pressure, dissolved in DMSO, and filtered, and formic acid (300 μL) was added. Purification of the reaction mixture by reverse-phase chromatography (10% → 60% MeCN/H_2_O) afforded the desired product (21.0 mg, 25% yield) as a colorless amorphous solid. LCMS (ESI) *m*/*z*: [M+H] calcd for C_88_H_132_N_12_O_20_ 1677.98; found 1677.9.

#### *N*-(4-(4-Amino-3-(5-hydroxy-1*H*-indol-2-yl)-1*H*-pyrazolo[3,4-*d*]pyrimidin-1-yl)butyl)-1-(4-(4-(((1*R*,2*R*,4*S*)-4-((*R*)-2-((3*S*,6*R*,7*E*,9*R*,10*R*,12*R*,14*S*,15*E*,17*E*,19*E*,21*S*,23*S*,26*R*,27*R*,34a*S*)-9,27-dihydroxy-10,21-ddimethoxy-6,8,12,14,20,26-hexamethyl-1,5,11,28,29-pentaoxo-1,4,5,6,9,10,11,12,13,14,21,22,23,24,25,26,27,28,29,31,32,33,34,34a-tetracosahydro-3*H*-23,27-epoxypyrido[2,1-*c*][1]oxa[4]azacyclohentriacontin-3-yl)propyl)-2-methoxycyclohexyl)oxy)butyl)-1*H*-1,2,3-triazol-1-yl)-3,6,9,12,15,18-hexaoxahenicosan-21-amide (**25**)

To a solution of **10** (50 mg, 50.2 μmol, 1.0 equiv) and **SI-14** (43.8, 62.7 μmol, 1.3 equiv) in MeOH (10 mL) was added a 1 M solution of CuSO_4_ in H_2_O (0.187 mL, 187 μmol, 3.7 equiv) followed by a 1 M solution of sodium ascorbate in H_2_O (0.251 mL, 251 μmol, 5 equiv). After stirring overnight, the reaction mixture was concentrated under reduced pressure, dissolved in DMSO, and filtered, and formic acid (300 μL) was added. Purification of the reaction mixture by reverse-phase chromatography (10% → 60% MeCN/H_2_O) afforded the desired product (16.2 mg, 19% yield) as a colorless amorphous solid. LCMS (ESI) *m*/*z*: [M+H] calcd for C_89_H_133_N_11_O_21_ 1692.98; found 1692.9.

#### (3*S*,6*R*,7*E*,9*R*,10*R*,12*R*,14*S*,15*E*,17*E*,19*E*,21*S*,23*S*,26*R*,27*R*,34a*S*)-9,27-Dihydroxy-10,21-ddimethoxy-3-((*R*)-1-((1*S*,3*R*,4*R*)-3-methoxy-4-(4-(1-(21-(4-(4-(8-(6-methoxypyridin-3-yl)-3-methyl-2-oxo-2,3-dihydro-1*H*-imidazo[4,5-*c*]quinolin-1-yl)-2-(trifluoromethyl)phenyl)piperazin-1-yl)-21-oxo-3,6,9,12,15,18-hexaoxahenicosyl)-1*H*-1,2,3-triazol-4-yl)butoxy)cyclohexyl)propan-2-yl)-6,8,12,14,20,26-hexamethyl-9,10,12,13,14,21,22,23,24,25,26,27,32,33,34,34a-hexadecahydro-3*H*-23,27-epoxypyrido[2,1-*c*][1]oxa[4]azacyclohentriacontine-1,5,11,28,29(4*H*,6*H*,31*H*)-pentaone (**26**)

To a solution of **10** (40 mg, 40.8 μmol, 1.0 equiv) and **SI-16** (40.1, 40.8 μmol, 1.0 equiv) in MeOH (8 mL) was added a 1 M solution of CuSO_4_ in H_2_O (0.150 mL, 0.150 μmol, 3.7 equiv) followed by a 1 M solution of sodium ascorbate in H_2_O (0.203 mL, 203 μmol, 5 equiv). After stirring overnight, the reaction mixture was concentrated under reduced pressure, dissolved in DMSO, and filtered, and formic acid (300 μL) was added. Purification of the reaction mixture by reverse-phase chromatography (10% → 60% MeCN/H_2_O) afforded the desired product (29.7 mg, 37% yield) as a colorless amorphous solid. LCMS (ESI) *m*/*z*: [M+H] calcd for C_100_H_139_F_3_N_10_O_22_ 1890.1; found 1890.0.

#### *N*-(4-(4-Amino-3-(2-aminobenzo[*d*]oxazol-5-yl)-1*H*-pyrazolo[3,4-*d*]pyrimidin-1-yl)butyl)-1-(4-(4-(((1*R*,2*R*,4*S*)-4-((*R*)-2-((3*S*,6*R*,7*E*,9*R*,10*R*,12*R*,14*S*,15*E*,17*E*,19*E*,21*S*,23*S*,26*R*,27*R*,34a*S*)-9,27-dihydroxy-10,21-ddimethoxy-6,8,12,14,20,26-hexamethyl-1,5,11,28,29-pentaoxo-1,4,5,6,9,10,11,12,13,14,21,22,23,24,25,26,27,28,29,31,32,33,34,34a-tetracosahydro-3*H*-23,27-epoxypyrido[2,1-*c*][1]oxa[4]azacyclohentriacontin-3-yl)propyl)-2-methoxycyclohexyl)oxy)butyl)-1*H*-1,2,3-triazol-1-yl)-3,6,9,12,15,18-hexaoxahenicosan-21-amide (**27**)

To a solution of **10** (54 mg, 54.3 μmol, 1.0 equiv) and **SI-8** (45.5 mg, 65.1 μmol, 1.2 equiv) in MeOH (10.5 mL) was added a 1 M solution of CuSO_4_ in H_2_O (200 μmol, 5 equiv) followed by a 1 M solution of sodium ascorbate in H_2_O (271 μmol, 5 equiv). After 2 h, the reaction mixture was concentrated under reduced pressure, dissolved in DMSO, and filtered, and formic acid (300 μL) was added. Purification of the reaction mixture by reverse-phase chromatography (10% → 65% MeCN/H_2_O) afforded the desired product (16.7 mg, 18% yield) as a colorless amorphous solid. LCMS (ESI) *m*/*z*: [M+H] calcd for C_88_H_132_N_12_O_21_ 1693.97; found 1694.0.

#### *N*-(4-(4-Amino-3-(2-aminobenzo[*d*]oxazol-5-yl)-1*H*-pyrazolo[3,4-*d*]pyrimidin-1-yl)butyl)-1-(4-(4-(((1*R*,2*R*,4*S*)-4-((*R*)-2-((3*S*,6*R*,7*E*,9*R*,10*R*,12*R*,14*S*,15*E*,17*E*,19*E*,21*S*,23*S*,26*R*,27*R*,34a*S*)-9,27-dihydroxy-10,21-ddimethoxy-6,8,12,14,20,26-hexamethyl-1,5,11,28,29-pentaoxo-1,4,5,6,9,10,11,12,13,14,21,22,23,24,25,26,27,28,29,31,32,33,34,34a-tetracosahydro-3*H*-23,27-epoxypyrido[2,1-*c*][1]oxa[4]azacyclohentriacontin-3-yl)propyl)-2-methoxycyclohexyl)oxy)butyl)-1*H*-1,2,3-triazol-1-yl)-3,6,9,12,15-pentaoxaoctadecan-18-amide (**28**)

To a solution of **10** (50 mg, 50.2 μmol, 1.0 equiv) and **SI-9** (39.4 mg, 60.2 μmol, 1.2 equiv) in MeOH (10 mL) was added a 1 M solution of CuSO_4_ in H_2_O (185 μmol, 3.7 equiv) followed by a 1 M solution of sodium ascorbate in H_2_O (251 μmol, 5 equiv). After stirring overnight, a 1 M solution of CuSO_4_ in H_2_O (185 μmol, 3.7 equiv) followed by a 1 M solution of sodium ascorbate in H_2_O (251 μmol, 5 equiv) was added. After stirring overnight, the reaction mixture was concentrated under reduced pressure, dissolved in DMSO, and filtered, and formic acid (300 μL) was added. Purification of the reaction mixture by reverse-phase chromatography (10% → 60% MeCN/H_2_O) afforded the desired product (24.5 mg, 30% yield) as a colorless amorphous solid. LCMS (ESI) *m*/*z*: [M+H] calcd for C_86_H_128_N_12_O_20_ 1649.94; found 1650.0.

#### (1*R*,2*R*,4*S*)-4-((*R*)-2-((3*S*,6*R*,7*E*,9*R*,10*R*,12*R*,14*S*,15*E*,17*E*,19*E*,21*S*,23*S*,26*R*,27*R*,34a*S*)-9,27-Dihydroxy-10,21-dimethoxy-6,8,12,14,20,26-hexamethyl-1,5,11,28,29-pentaoxo-1,4,5,6,9,10,11,12,13,14,21,22,23,24,25,26,27,28,29,31,32,33,34,34a-tetracosahydro-3*H*-23,27-epoxypyrido[2,1-*c*][1]oxa[4]azacyclohentriacontin-3-yl)propyl)-2-methoxycyclohexyl (32-(4-amino-3-(2-aminobenzo[*d*]oxazol-5-yl)-1*H*-pyrazolo[3,4-*d*]pyrimidin-1-yl)-27-oxo-3,6,9,12,15,18,21,24-octaoxa-28-azadotriacontyl)carbamate (**29**)

To a solution of **13** (22 mg, 20.3 μmol, 1.0 equiv) and **SI-34** (44.4 mg, 50.7 μmol, 2.5 equiv) in DMA (0.2 mL) was added DIPEA (17.5 μL, 1.1 μmol, 5.0 equiv). The reaction was stirred for 7 h, at which point the reaction mixture was purified by reverse-phase chromatography (40% → 100% MeCN/H_2_O) to afford the desired product (16.7 mg, 48% yield) as a colorless amorphous solid. LCMS (ESI) *m*/*z*: [M+H] calcd for C_87_H_132_N_10_O_24_ 1701.95; found 1701.8.

#### (1*R*,2*R*,4*S*)-4-((*R*)-2-((3*S*,6*R*,7*E*,9*R*,10*R*,12*R*,14*S*,15*E*,17*E*,19*E*,21*S*,23*S*,26*R*,27*R*,34a*S*)-9,27-Dihydroxy-10,21-ddimethoxy-6,8,12,14,20,26-hexamethyl-1,5,11,28,29-pentaoxo-1,4,5,6,9,10,11,12,13,14,21,22,23,24,25,26,27,28,29,31,32,33,34,34a-tetracosahydro-3*H*-23,27-epoxypyrido[2,1-*c*][1]oxa[4]azacyclohentriacontin-3-yl)propyl)-2-methoxycyclohexyl (27-(6-((4-amino-3-(2-aminobenzo[*d*]oxazol-5-yl)-1*H*-pyrazolo[3,4-*d*]pyrimidin-1-yl)methyl)-3,4-dihydroisoquinolin-2(1*H*)-yl)-27-oxo-3,6,9,12,15,18,21,24-octaoxaheptacosyl)carbamate (**30**)

To a solution of **SI-36** (774 mg, 0.926 mmol, 2.0 equiv) in DMA (2.3 mL) at 0 °C was added DIPEA (322 μL, 1.85 mmol, 4.0 equiv) followed by **13** (500.0 mg, 0.463 mmol, 1.0 equiv). The reaction mixture was warmed to room temperature and stirred for 5 h. The reaction mixture was acidified with formic acid and purified by reverse-phase chromatography (40% → 100% MeCN/H_2_O) to afford the desired product (500 mg, 61% yield) as a colorless amorphous solid. LCMS (ESI) *m*/*z*: [M+H] calcd for C_93_H_134_N_10_O_24_ 1775.97; found 1775.9.

#### (1*R*,2*R*,4*S*)-4-((*R*)-2-((3*S*,6*R*,7*E*,9*R*,10*R*,12*R*,14*S*,15*E*,17*E*,19*E*,21*S*,23*S*,26*R*,27*R*,34a*S*)-9,27-Dihydroxy-10,21-ddimethoxy-6,8,12,14,20,26-hexamethyl-1,5,11,28,29-pentaoxo-1,4,5,6,9,10,11,12,13,14,21,22,23,24,25,26,27,28,29,31,32,33,34,34a-tetracosahydro-3*H*-23,27-epoxypyrido[2,1-*c*][1]oxa[4]azacyclohentriacontin-3-yl)propyl)-2-methoxycyclohexyl (30-((4-(7-(6-aminopyridin-3-yl)-2,3,4,5-tetrahydrobenzo[*f*][1,4]oxazepine-4-carbonyl)-2-fluoro-3-methylphenyl)sulfonyl)-27-oxo-3,6,9,12,15,18,21,24-octaoxa-28-azatriacontyl)carbamate (**31**)

To a solution of **SI-44** (650 mg, 715 μmol, 1.8 equiv) in DMA (8 mL) was added DIPEA (343 μL, 1.98 mmol, 5 equiv) followed by **13** (428 mg, 397 μmol, 1.0 equiv). The reaction mixture was stirred overnight at room temperature. The reaction mixture was acidified with formic acid and purified by reverse-phase chromatography (40% → 100% MeCN/H_2_O) to afford the desired product (344 mg, 47% yield) as a colorless amorphous solid. LCMS (ESI) *m*/*z*: [M+H] calcd for C_95_H_139_FN_6_O_27_S 1847.95; found 1848.0.

#### (*S*)-1-(2-((2*R*,3*R*,6*S*)-6-((2*S*,3*E*,5*E*,7*E*,9*S*,11*R*,13*R*,14*R*,15*E*,17*R*,21*R*)-22-((1*S*,3*R*,4*R*)-4-(4-(1-(32-(4-Amino-3-(2-aminobenzo[*d*]oxazol-5-yl)-1*H*-pyrazolo[3,4-*d*]pyrimidin-1-yl)-27-oxo-3,6,9,12,15,18,21,24-octaoxa-28-azadotriacontyl)-1*H*-1,2,3-triazol-4-yl)butoxy)-3-methoxycyclohexyl)-14-hydroxy-2,13-ddimethoxy-3,9,11,15,17,21-hexamethyl-12,18-dioxodocosa-3,5,7,15,19-pentaen-1-yl)-2-hydroxy-3-methyltetrahydro-2*H*-pyran-2-yl)-2-oxoacetyl)piperidine-2-carboxylic Acid (**32**)

To a solution of **16** (70.6 mg, 39.6 μmol, 1.0 equiv) in DMA (2 mL) was added NH_4_OAc (30.5 mg, 396 μmol, 10 equiv). The reaction mixture was heated to 40 °C, and after 6 h it was cooled to room temperature. Purification of the reaction mixture by reverse-phase chromatography (40% → 100% MeCN/H_2_O) afforded the desired product (19.4 mg, 28% yield, 99.5% purity) as a colorless amorphous solid. LCMS (ESI) *m*/*z*: [M+Na] calcd for C_92_H_140_N_12_O_23_ 1804.01; found 1804.0.

#### (*S*)-1-(2-((2*R*,3*R*,6*S*)-6-((2*S*,3*E*,5*E*,7*E*,9*S*,11*R*,13*R*,14*R*,15*E*,17*R*,19*Z*,21*R*)-22-((1*S*,3*R*,4*R*)-4-(((27-(6-((4-Amino-3-(2-aminobenzo[*d*]oxazol-5-yl)-1*H*-pyrazolo[3,4-*d*]pyrimidin-1-yl)methyl)-3,4-dihydroisoquinolin-2(1*H*)-yl)-27-oxo-3,6,9,12,15,18,21,24-octaoxaheptacosyl)carbamoyl)oxy)-3-methoxycyclohexyl)-14-hydroxy-2,13-ddimethoxy-3,9,11,15,17,21-hexamethyl-12,18-dioxodocosa-3,5,7,15,19-pentaen-1-yl)-2-hydroxy-3-methyltetrahydro-2*H*-pyran-2-yl)-2-oxoacetyl)piperidine-2-carboxylic Acid (**33**)

To a solution of **30** (50 mg, 28.1 μmol, 1.0 equiv) in DMA (1.4 mL) was added NH_4_OAc (21.6 mg, 280 μmol, 10 equiv). The reaction mixture was heated to 40 °C, and after 5 h it was cooled to room temperature. Purification of the reaction mixture by reverse-phase chromatography (10% → 100% MeCN/H_2_O) afforded the desired product (13.8 mg, 28% yield, 99.3% purity) as a colorless amorphous solid. LCMS (ESI) *m*/*z*: [M+Na] calcd for C_93_H_134_N_10_O_24_ 1775.97; found 1775.8.

#### (1*R*,2*R*,4*S*)-4-((*R*)-2-((3*S*,5*R*,6*R*,7*E*,9*R*,10*R*,12*R*,14*S*,15*E*,17*E*,19*E*,21*S*,23*S*,26*R*,27*R*,34a*S*)-9,27-Dihydroxy-5,10,21-trdimethoxy-6,8,12,14,20,26-hexamethyl-1,11,28,29-tetraoxo-1,4,5,6,9,10,11,12,13,14,21,22,23,24,25,26,27,28,29,31,32,33,34,34a-tetracosahydro-3*H*-23,27-epoxypyrido[2,1-*c*][1]oxa[4]azacyclohentriacontin-3-yl)propyl)-2-methoxycyclohexyl (32-(4-Amino-3-(2-aminobenzo[*d*]oxazol-5-yl)-1*H*-pyrazolo[3,4-*d*]pyrimidin-1-yl)-27-oxo-3,6,9,12,15,18,21,24-octaoxa-28-azadotriacontyl)carbamate (**35**)

To a solution of **15** (25 mg, 22.8 μmol, 1.0 equiv) and **SI-34** (34.7 mg, 45.6 μmol, 2.0 equiv) in DMA (1.15 mL) was added DIPEA (15.7 μL, 91.2 μmol, 4.0 equiv). The reaction was stirred overnight, and then the reaction mixture was purified by reverse-phase chromatography (40% → 100% MeCN/H_2_O) to afford the desired product (24.5 mg, 63% yield) as a colorless amorphous solid. LCMS (ESI) *m*/*z*: [M+H] calcd for C_88_H_136_N_10_O_24_ 1717.98; found 1718.0.

#### (1*R*,2*R*,4*S*)-2-methoxy-4-((*R*)-2-((3*S*,5*R*,6*R*,7*E*,9*R*,10*R*,12*R*,14*S*,15*E*,17*E*,19*E*,21*S*,23*S*,26*R*,27*R*,34a*S*)-5,9,27-Trihydroxy-10,21-ddimethoxy-6,8,12,14,20,26-hexamethyl-1,11,28,29-tetraoxo-1,4,5,6,9,10,11,12,13,14,21,22,23,24,25,26,27,28,29,31,32,33,34,34a-tetracosahydro-3*H*-23,27-epoxypyrido[2,1-*c*][1]oxa[4]azacyclohentriacontin-3-yl)propyl)cyclohexyl (32-(4-Amino-3-(2-aminobenzo[*d*]oxazol-5-yl)-1*H*-pyrazolo[3,4-*d*]pyrimidin-1-yl)-27-oxo-3,6,9,12,15,18,21,24-octaoxa-28-azadotriacontyl)carbamate (**36**)

To a solution of **14** (25 mg, 23.1 μmol, 1.0 equiv) and **SI-34** (35.1 mg, 231 μmol, 2.0 equiv) in DMA (1.15 mL) was added DIPEA (16.0 μL, 92.4 μmol, 4.0 equiv). The reaction was stirred overnight, and then the reaction mixture was purified by reverse-phase chromatography (40% → 100% MeCN/H_2_O) to afford the desired product (15.4 mg, 39% yield) as a colorless amorphous solid. LCMS (ESI) *m*/*z*: [M+H] calcd for C_87_H_134_N_10_O_24_ 1703.97; found 1703.7.

#### (1*R*,2*R*,4*S*)-4-((*R*)-2-((3*S*,5*R*,6*R*,7*E*,9*R*,10*R*,12*R*,14*S*,15*E*,17*E*,19*E*,21*S*,23*S*,26*R*,27*R*,34a*S*)-9,27-Dihydroxy-5,10,21-trdimethoxy-6,8,12,14,20,26-hexamethyl-1,11,28,29-tetraoxo-1,4,5,6,9,10,11,12,13,14,21,22,23,24,25,26,27,28,29,31,32,33,34,34a-tetracosahydro-3*H*-23,27-epoxypyrido[2,1-*c*][1]oxa[4]azacyclohentriacontin-3-yl)propyl)-2-methoxycyclohexyl (27-(6-((4-Amino-3-(2-aminobenzo[*d*]oxazol-5-yl)-1*H*-pyrazolo[3,4-*d*]pyrimidin-1-yl)methyl)-3,4-dihydroisoquinolin-2(1*H*)-yl)-27-oxo-3,6,9,12,15,18,21,24-octaoxaheptacosyl)carbamate (**37**)

To a solution of **SI-36** (250 mg, 299 μmol, 2.2 equiv) in DMA (6.8 mL) was added DIPEA (118 μL, 680 μmol, 5 equiv) followed by **15** (150 mg, 136 μmol, 1.0 equiv). The reaction mixture was stirred overnight at room temperature. The reaction mixture was acidified with formic acid and purified by reverse-phase chromatography (40% → 100% MeCN/H_2_O) to afford the desired product (108 mg, 44% yield) as a colorless amorphous solid. LCMS (ESI) *m*/*z*: [M+H] calcd for C_93_H_136_N_10_O_24_ 1777.98; found 1777.7.

#### (1*R*,2*R*,4*S*)-2-Methoxy-4-((*R*)-2-((3*S*,5*R*,6*R*,7*E*,9*R*,10*R*,12*R*,14*S*,15*E*,17*E*,19*E*,21*S*,23*S*,26*R*,27*R*,34a*S*)-5,9,27-trihydroxy-10,21-ddimethoxy-6,8,12,14,20,26-hexamethyl-1,11,28,29-tetraoxo-1,4,5,6,9,10,11,12,13,14,21,22,23,24,25,26,27,28,29,31,32,33,34,34a-tetracosahydro-3*H*-23,27-epoxypyrido[2,1-*c*][1]oxa[4]azacyclohentriacontin-3-yl)propyl)cyclohexyl (27-(6-((4-Amino-3-(2-aminobenzo[*d*]oxazol-5-yl)-1*H*-pyrazolo[3,4-*d*]pyrimidin-1-yl)methyl)-3,4-dihydroisoquinolin-2(1*H*)-yl)-27-oxo-3,6,9,12,15,18,21,24-octaoxaheptacosyl)carbamate (**38**)

To a solution of **SI-36** (463 mg, 0.555 mmol, 2.0 equiv) in DMA (1.4 mL) at 0 °C was added DIPEA (191 μL, 1.1 mmol, 4.0 equiv) followed by **14** (300.0 mg, 0.277 mmol, 1.0 equiv). The reaction mixture was warmed to room temperature and stirred for 5 h. The reaction mixture was acidified with formic acid and purified by reverse-phase chromatography (40% → 100% MeCN/H_2_O) to afford the desired product (179 mg, 36% yield) as a colorless amorphous solid. HRMS (ESI) *m*/*z*: [M+H] calcd for C_93_H_136_N_10_O_24_ 1777.9807; found 1777.9813.

#### (1*R*,2*R*,4*S*)-4-((*R*)-2-((3*S*,5*R*,6*R*,7*E*,9*R*,10*R*,12*R*,14*S*,15*E*,17*E*,19*E*,21*S*,23*S*,26*R*,27*R*,34a*S*)-9,27-Dihydroxy-5,10,21-trdimethoxy-6,8,12,14,20,26-hexamethyl-1,11,28,29-tetraoxo-1,4,5,6,9,10,11,12,13,14,21,22,23,24,25,26,27,28,29,31,32,33,34,34a-tetracosahydro-3*H*-23,27-epoxypyrido[2,1-*c*][1]oxa[4]azacyclohentriacontin-3-yl)propyl)-2-methoxycyclohexyl (30-((4-(7-(6-Aminopyridin-3-yl)-2,3,4,5-tetrahydrobenzo[*f*][1,4]oxazepine-4-carbonyl)-2-fluoro-3-methylphenyl)sulfonyl)-27-oxo-3,6,9,12,15,18,21,24-octaoxa-28-azatriacontyl)carbamate (**39**)

To a solution of **SI-44** (55.8 mg, 54.6 μmol, 2.0 equiv) in DMA (1.4 mL) at 0 °C was added DIPEA (28.3 μL, 163 μmol, 6 equiv) followed by **15** (30 mg, 27.3 μmol, 1.0 equiv). The reaction mixture was stirred overnight at room temperature. The reaction mixture was acidified with formic acid and purified by reverse-phase chromatography (10% → 100% MeCN/H_2_O) to afford the desired product (29.5 mg, 58% yield) as a colorless amorphous solid. LCMS (ESI) *m*/*z*: [M+H] calcd for C_96_H_143_FN_6_O_27_S 1863.98 found 1864.0.

#### (1*R*,2*R*,4*S*)-2-Methoxy-4-((*R*)-2-((3*S*,5*R*,6*R*,7*E*,9*R*,10*R*,12*R*,14*S*,15*E*,17*E*,19*E*,21*S*,23*S*,26*R*,27*R*,34a*S*)-5,9,27-trihydroxy-10,21-ddimethoxy-6,8,12,14,20,26-hexamethyl-1,11,28,29-tetraoxo-1,4,5,6,9,10,11,12,13,14,21,22,23,24,25,26,27,28,29,31,32,33,34,34a-tetracosahydro-3*H*-23,27-epoxypyrido[2,1-*c*][1]oxa[4]azacyclohentriacontin-3-yl)propyl)cyclohexyl (30-((4-(7-(6-Aminopyridin-3-yl)-2,3,4,5-tetrahydrobenzo[*f*][1,4]oxazepine-4-carbonyl)-2-fluoro-3-methylphenyl)sulfonyl)-27-oxo-3,6,9,12,15,18,21,24-octaoxa-28-azatriacontyl)carbamate (**40**)

To a solution of **SI-44** (630 mg, 667 μmol, 2.0 equiv) in DMA (6.7 mL) at 0 °C was added DIPEA (462 μL, 2.66 mmol, 8 equiv) followed by **14** (360 mg, 334 μmol, 1.0 equiv). The reaction mixture was stirred overnight at room temperature. The reaction mixture was acidified with formic acid and purified by reverse-phase chromatography (10% → 90% MeCN/H_2_O) to afford the desired product (345 mg, 56% yield) as a colorless amorphous solid. LCMS (ESI) *m*/*z*: [M+H] calcd for C_95_H_141_FN_6_O_27_S 1849.96 found 1850.0.

## Supporting Information Available

Details of data collection, processing, and structure refinement statistics for the protein crystal structures of **11** and **12**; biochemical and cellular assay protocols; synthetic procedures for intermediates; NMR characterization of RMC-5552 **38**, analytical HPLC traces of RMC-5552 **38** and RMC-6272 **40**; in vitro characterization of RMC-5552 **38** and RMC-6272 **40** (PDF)
Molecular formula strings (CSV)

## Accession Codes

PDB ID 8ER6, 8ER7, and 8ERA. Authors will release the atomic coordinates and experimental data upon article publication.

## Abbreviations Used

ANOVA: analysis of variance
CDX: cell line-derived xenograft
cryo-EM: cryogenic electron microscopy
4EBP1: eukaryotic initiation factor 4E-binding protein 1
eIF4E: eukaryotic translation initiation factor 4E
FKBP: FK binding protein
FRB: FKBP12-rapamycin-binding
KRAS: Kirsten rat sarcoma virus
mTOR: mechanistic target of rapamycin
mTORC1: mechanistic target of rapamycin complex 1
mTORC2: mechanistic target of rapamycin complex 2
NFAT: nuclear factor of activated T cells
PIKK: phosphatidylinositol 3-kinase-related kinase
PNP: *p*-nitrophenyl
Raptor: regulatory-associated protein of mTOR
RAS: rat sarcoma virus
Rictor: rapamycin-insensitive companion of mTOR
RTK: receptor tyrosine kinase
S6K: ribosomal protein S6 kinase
Sin1: stress-activated map kinase-interacting protein 1
TOR1: target of rapamycin 1
TOR2: target of rapamycin 2
TR-FRET: time-resolved fluorescence energy transfer

## References

[ref1] Sehgal S. N.; Blazekovic T. M.; Vezina C. Rapamycin und seine herstellung. Patent application number DE1973-2347682, 1973; *Chem Abstr.* **1974**, *81*, 24166.

[ref2] Vézina C.; Kudelski A.; Sehgal S. N. Rapamycin (AY-22,989), a new antifungal antibiotic. I. Taxonomy of the producing streptomycete and isolation of the active principle. J. Antibiot. 1975, 28, 721–726. 10.7164/antibiotics.28.721. 1102508

[ref3] Sehgal S. N.; Baker H.; Vézina C. Rapamycin (AY-22,989), a new antifungal antibiotic. II. Fermentation, isolation, and characterization. J. Antibiot. 1975, 28, 727–732. 10.7164/antibiotics.28.727. 1102509

[ref4] Sehgal S. N.; Blazekovic T. M.; Vézina C. Rapamycin and process of preparation. U.S. Patent 3,929,992, 1975; *Chem Abstr.* **1976**, *85*, 3861.

[ref5] Sehgal S. N.; Blazekovic T. M.; Vezina C. Rapamycin and process of preparation. U.S. Patent 3,993,749, 1976; *Chem Abstr.* **1977**, *86*, 41806.

[ref6] Sidorowicz H.; Baker H.; Vézina C. Rapamycin (AY-22,989), a new antifungal antibiotic: in vitro and in vivo studies. 15th Interscience Conference on Antimicrobial Agents and Chemotherapy, Sept 24–26, 1975, Washington, DC; American Society for Microbiology, 1975; Abstract 26.

[ref7] Baker H.; Sidorowicz A.; Sehgal S. N.; Vézina C. Rapamycin (AY-22,989), a new antifungal antibiotic. III. In vitro and in vivo evaluation. J. Antibiot. 1978, 31, 539–545. 10.7164/antibiotics.31.539. 28309

[ref8] Martel R. R.; Klicius J.; Galet S. Inhibition of the immune response by rapamycin, a new antifungal antibiotic. Can. J. Physiol. Pharmacol. 1977, 55, 48–51. 10.1139/y77-007. 843990

[ref9] Calne R. Y.; Collier D. S.; Lim S.; Pollard S. G.; Samaan A.; White D. J.; Thiru S. Rapamycin for immunosuppression in organ allografting. Lancet 1989, 334, 227 10.1016/S0140-6736(89)90417-0. 2568561

[ref10] Morris R. E.; Meiser B. M. Identification of a new pharmacologic action for an old compound. Med. Sci. Res. 1989, 17, 609–610.

[ref11] aSehgal S. N.; Vézina C. Compositions pharmaceutiques à base de rapamycine pour le traitement de tumeurs carcinogens. Patent number BE877700, 1980; *Chem Abstr.* **1980**, *93*, 88940. See also.

[ref12] Eng C. P. Combination of rapamycin and picibanil for the treatment of tumors. U.S. Patent 4,401,653, 1983; *Chem Abstr.* **1983**, *98*, 22284.

[ref13] Houchens D. P.; Ovejera A. A.; Riblet S. M.; Slagel D. E. Human brain tumor xenografts in nude mice as a chemotherapy model. Eur. J. Can. Clin. Oncol. 1983, 19, 799–805. 10.1016/0277-5379(83)90012-3. 6683650

[ref14] Venditti J. M.; Wesley R. A.; Plowman J. Current NCI preclinical antitumor screening in vivo: results of tumor panel screening, 1976–1982, and future directions. Advances in Pharmacology; Elsevier 1984, 20, 1–20. 10.1016/S1054-3589(08)60263-X. 6398966

[ref15] Eng C. P.; Sehgal S. N.; Vézina C. Activity of rapamycin (AY-22,989) against transplanted tumors. J. Antibiot. 1984, 37, 1231–1237. 10.7164/antibiotics.37.1231. 6501094

[ref16] Kino T.; Hatanaka H.; Hashimoto M.; Nishiyama M.; Goto T.; Okuhara M.; Kohsaka M.; Aoki H.; Imanaka H. FK-506, a novel immunosuppressant isolated from a Streptomyces. I. Fermentation, isolation, and physico-chemical and biological characteristics. J. Antibiot. 1987, 40, 1249–1255. 10.7164/antibiotics.40.1249. 2445721

[ref17] Kino T.; Hatanaka H.; Miyata S.; Inamura N.; Nishiyama M.; Yajima T.; Goto T.; Okuhara M.; Kohsaka M.; Aoki H.; Ochiai T. FK-506, a novel immunosuppressant isolated from a Streptomyces. II. Immunosuppressive effect of FK-506 in vitro. J. Antibiot. 1987, 40, 1256–1265. 10.7164/antibiotics.40.1256. 2445722

[ref18] Siekierka J. J.; Hung S. H. Y.; Poe M.; Lin C. S.; Sigal N. H. A cytosolic binding protein for the immunosuppressant FK506 has peptidyl-prolyl isomerase activity but is distinct from cyclophilin. Nature 1989, 341, 755–757. 10.1038/341755a0. 2477714

[ref19] Harding M. W.; Galat A.; Uehling D. E.; Schreiber S. L. A receptor for the immuno-suppressant FK506 is a cis-trans peptidyl-prolyl isomerase. Nature 1989, 341, 758–760. 10.1038/341758a0. 2477715

[ref20] Bierer B. E.; Mattila P. S.; Standaert R. F.; Herzenberg L. A.; Burakoff S. J.; Crabtree G.; Schreiber S. L. Two distinct signal transmission pathways in T lymphocytes are inhibited by complexes formed between an immunophilin and either FK506 or rapamycin. Proc. Natl. Acad. Sci. U.S.A. 1990, 87, 9231–9235. 10.1073/pnas.87.23.9231. 2123553 PMC55138

[ref21] Van Duyne G. D.; Standaert R. F.; Schreiber S. L.; Clardy J. Atomic structure of the rapamycin human immunophilin FKBP-12 complex. J. Am. Chem. Soc. 1991, 113, 7433–7434. 10.1021/ja00019a057.

[ref22] Liu J.; Farmer J. D. Jr; Lane W. S.; Friedman J.; Weissman I.; Schreiber S. L. Calcineurin is a common target of cyclophilin-cyclosporin A and FKBP-FK506 complexes. Cell 1991, 66, 807–815. 10.1016/0092-8674(91)90124-H. 1715244

[ref23] Morris R. E. Rapamycin: FK506’s fraternal twin or distant cousin?. Immonol. Today 1991, 12, 137–140. and references cited therein 10.1016/S0167-5699(05)80040-4. 1715165

[ref24] Heitman J.; Movva N. R.; Hall M. N. Targets for cell cycle arrest by the immunosuppressant rapamycin in yeast. Science 1991, 253, 905–909. 10.1126/science.1715094. 1715094

[ref25] Brown E. J.; Albers M. W.; Bum Shin T.; ichikawa K.; Keith C. T.; Lane W. S.; Schreiber S. L. A mammalian protein targeted by G1-arresting rapamycin receptor complex. Nature 1994, 369, 756–758. 10.1038/369756a0. 8008069

[ref26] Sabatini D. M.; Erdjument-Bromage H.; Lui M.; Tempst P.; Snyder S. H. RAFT1: A mammalian protein that binds to FKBP12 in a rapamycin-dependent fashion and is homologous to yeast TORs. Cell 1994, 78, 35–43. 10.1016/0092-8674(94)90570-3. 7518356

[ref27] Sabers C. J.; Martin M. M.; Brunn G. J.; Williams J. M.; Dumont F. J.; Wiederrecht G.; Abraham R. T. Isolation of a protein target of the FKBP12-rapamycin complex in mammalian cells. J. Biol. Chem. 1995, 270, 815–822. 10.1074/jbc.270.2.815. 7822316

[ref28] FRAP subfamily: mechanistic target of rapamycin kinase. IUPHAR/BPS Guide to PHARMACOLOGY; Last modified Feb 28, 2020 (accessed Aug 6, 2022); http://www.guidetopharmacology.org/GRAC/ObjectDisplayForward?objectId=2109.

[ref29] Kunz J.; Henriquez R.; Schneider U.; Deuter-Reinhard M.; Movva N. R.; Hall M. N. Target of rapamycin in yeast, TOR2, is an essential phosphatidylinositol kinase homolog required for G1 progression. Cell 1993, 73, 585–596. 10.1016/0092-8674(93)90144-F. 8387896

[ref30] Walker E. H.; Perisic O.; Ried C.; Stephens L.; Williams R. L. Structural insights into phosphoinositide 3-kinase catalysis and signalling. Nature 1999, 402, 313–320. 10.1038/46319. 10580505

[ref31] Yang H.; Rudge D. G.; Koos J. D.; Vaidialingam B.; Yang H. J.; Pavletich N. P. mTOR kinase structure, mechanism and regulation. Nature 2013, 497, 217–223. 10.1038/nature12122. 23636326 PMC4512754

[ref32] Chen J.; Zheng X.-F.; Brown E. J.; Schreiber S. L. Identification of an 11-kDa FKBP12-rapamycin-binding domain within the 289-kDa FKBP12-rapamycin-associated protein and characterization of a critical serine residue. Proc. Natl. Acad. Sci. U.S.A. 1995, 92, 4947–4951. 10.1073/pnas.92.11.4947. 7539137 PMC41824

[ref33] Yang Q.; Guan K.-L. Expanding mTOR signaling. Cell Res. 2007, 17, 666–681. 10.1038/cr.2007.64. 17680028

[ref34] Loewith R.; Jacinto E.; Wullschleger S.; Lorberg A.; Crespo J. L.; Bonenfant D.; Oppliger W.; Jenoe P.; Hall M. N. Two TOR complexes, only one of which is rapamycin sensitive, have distinct roles in cell growth control. Mol. Cell 2002, 10, 457–468. 10.1016/S1097-2765(02)00636-6. 12408816

[ref35] Kim D.-H.; Sarbassov D. D.; Ali S. M.; King J. E.; Latek R. R.; Erdjument-Bromage H.; Tempst P.; Sabatini D. M. mTOR interacts with raptor to form a nutrient-sensitive complex that signals to the cell growth machinery. Cell 2002, 110, 163–175. 10.1016/S0092-8674(02)00808-5. 12150925

[ref36] Brunn G. J.; Hudson C. C.; Sekulić A.; Williams J. M.; Hosoi H.; Houghton P. J.; Lawrence J. C.; Abraham R. T. Phosphorylation of the translational repressor PHAS-I by the mammalian target of rapamycin. Science 1997, 277, 99–101. 10.1126/science.277.5322.99. 9204908

[ref37] Burnett P. E.; Barrow R. K.; Cohen N. A.; Snyder S. H.; Sabatini D. M. RAFT1 phosphorylation of the translational regulators p70 S6 kinase and 4E-BP1. Proc. Natl. Acad. Sci. U.S.A. 1998, 95, 1432–1437. 10.1073/pnas.95.4.1432. 9465032 PMC19032

[ref38] Isotani S.; Hara K.; Tokunaga C.; Inoue H.; Avruch J.; Yonezawa K. Immunopurified mammalian target of rapamycin phosphorylates and activates p70 S6 kinase α in vitro. J. Biol. Chem. 1999, 274, 34493–34498. 10.1074/jbc.274.48.34493. 10567431

[ref39] Nojima H.; Tokunaga C.; Eguchi S.; Oshiro N.; Hidayat S.; Yoshino K.; Hara K.; Tanaka N.; Avruch J.; Yonezawa K. The mammalian target of rapamycin (mTOR) partner, raptor, binds the mTOR substrates p70 S6 kinase and 4E-BP1 through their TOR signaling (TOS) motif. J. Biol. Chem. 2003, 278, 15461–15464. 10.1074/jbc.C200665200. 12604610

[ref40] Musa J.; Orth M. F.; Dallmayer M.; Baldauf M.; Pardo C.; Rotblat B.; Kirchner T.; Leprivier G.; Grünewald T. G. P. Eukaryotic initiation factor 4E-binding protein 1 (4E-BP1): a master regulator of mRNA translation involved in tumorigenesis. Oncogene 2016, 35, 4675–4688. and references cited therein 10.1038/onc.2015.515. 26829052

[ref41] Sarbassov D. D.; Ali S. M.; Kim D.-H.; Guertin D. A.; Latek R. R.; Erdjument-Bromage H.; Tempst P.; Sabatini D. M. Rictor, a novel binding partner of mTOR, defines a rapamycin-insensitive and Raptor-independent pathway that regulates the cytoskeleton. Curr. Biol. 2004, 14, 1296–1302. 10.1016/j.cub.2004.06.054. 15268862

[ref42] Jacinto E.; Loewith R.; Schmidt A.; Lin S.; Rüegg M. A.; Hall A.; Hall M. N. Mammalian TOR complex 2 controls the actin cytoskeleton and is rapamycin insensitive. Nat. Cell Biol. 2004, 6, 1122–1128. 10.1038/ncb1183. 15467718

[ref43] Jacinto E.; Facchinetti V.; Liu D.; Soto N.; Wei S.; Jung S. Y.; Huang Q.; Qin J.; Su B. SIN1/MIP1 maintains rictor-mTOR complex integrity and regulates Akt phosphorylation and substrate specificity. Cell 2006, 127, 125–137. 10.1016/j.cell.2006.08.033. 16962653

[ref44] Sarbassov D. D.; Guertin D. A.; Ali S. M.; Sabatini D. M. Phosphorylation and regulation of Akt/PKB by the rictor-mTOR complex. Science 2005, 307, 1098–1101. 10.1126/science.1106148. 15718470

[ref45] Saxton R. A.; Sabatini D. M. mTOR signaling in growth, metabolism, and disease. Cell 2017, 168, 960–976. 10.1016/j.cell.2017.02.004. 28283069 PMC5394987

[ref46] https://www.accessdata.fda.gov/drugsatfda_docs/nda/99/21083A.cfm (accessed July 23, 2022).

[ref47] Chen Y.; Zhou X. Research progress of mTOR inhibitors. Eur. J. Med. Chem. 2020, 208, 112820 10.1016/j.ejmech.2020.112820. 32966896

[ref48] Choo A. Y.; Yoon S.-O.; Kim S. G.; Roux P. P.; Blenis J. Rapamycin differentially inhibits S6Ks and 4E-BP1 to mediate cell-type-specific repression of mRNA translation. Proc. Natl. Acad. Sci. U.S.A. 2008, 105, 17414–17419. 10.1073/pnas.0809136105. 18955708 PMC2582304

[ref49] Zhang Y.; Yan H.; Xu Z.; Yang B.; Luo P.; He Q. Molecular basis for class side effects associated with PI3K/AKT/mTOR pathway inhibitors. Expert Opin. Drug Metab. Toxicol. 2019, 15, 767–774. 10.1080/17425255.2019.1663169. 31478386

[ref50] Rodrik-Outmezguine V. S.; Okaniwa M.; Yao Z.; Novotny C. J.; McWhirter C.; Banaji A.; Won H.; Wong W.; Berger M.; de Stanchina E.; Barratt D. G.; Cosulich S.; Klinowska T.; Rosen N.; Shokat K. M. Overcoming mTOR resistance mutations with a new-generation mTOR inhibitor. Nature 2016, 534, 272–276. 10.1038/nature17963. 27279227 PMC4902179

[ref51] Shokat K.; Okaniwa M. mTORCl inhibitors. WO2016040806, 2016; *Chem Abstr.* **2016**, *164*, 412316.

[ref52] Jessen K.; Wang S.; Kessler L.; Guo X.; Kucharski J.; Staunton J.; Lan L.; Elia M.; Stewart J.; Brown J.; Li L.; Chan K.; Martin M.; Ren P.; Rommel C.; Liu Y. INK128 is a potent and selective TORC1/2 inhibitor with broad oral antitumor activity. Mol. Cancer Ther. 2009, 8, B148 10.1158/1535-7163.TARG-09-B148.

[ref53] Ren P.; Liu Y.; Li L.; Chan K.; Wilson T. E. Benzoxazole kinase inhibitors and methods of use. WO2010051043, 2010; *Chem Abstr.* **2010**, *152*, 542024.

[ref54] Semko C.; Pitzen J.; Wang G.; Tibrewal N.; Aggen J. B.; Thottumkara A. P.; Burnett G. L.; Gliedt M. J. E.; Kiss G.; Won W.; Lee J. C.; Gill A. L. Rapamycin analogs as mTOR inhibitors. WO2018204416, 2018; *Chem Abstr.* **2018**, *169*, 525729.

[ref55] Pitzen J.; Gliedt M. J. E.; Burnett G. L.; Aggen J. B.; Kiss G.; Cregg J. J.; Semko C. M.; Won W.; Wang G.; Lee J. C-L.; Thottumkara A. P.; Gill A. L.; Mellem K. T. C40-, C28-, and C32-linked rapamycin analogs as mTOR inhibitors. WO2019212990, 2019; *Chem Abstr.* **2019**, *171*, 558403.

[ref56] Semko C. M.; Wang G.; Burnett G. L.; Aggen J. B.; Kiss G.; Cregg J. J.; Gliedt M. J. E.; Pitzen J.; Lee J. C-L.; Won W.; Thottumkara A. P.; Gill A. L. C26-linked rapamycin analogs as mTOR inhibitors. WO2019212991, 2019; *Chem Abstr.* **2019**, *171*, 551179.

[ref57] Lee B. J.; Mallya S.; Dinglasan N.; Fung A.; Nguyen T.; Herzog L.; Thao J.; Lorenzana E. G.; Wildes D.; Singh M.; Smith J. A. M.; Fruman D. A. Efficacy of a novel bi-steric mTORC1 inhibitor in models of B-cell acute lymphoblastic leukemia. Front. Oncol. 2021, 11, 673213 10.3389/fonc.2021.673213. 34408976 PMC8366290

[ref58] Lee B. J.; Boyer J. A.; Burnett G. L.; Thottumkara A. P.; Tibrewal N.; Wilson S. L.; Hsieh T.; Marquez A.; Lorenzana E. G.; Evans J. W.; Hulea L.; Kiss G.; Liu H.; Lee D.; Larsson O.; McLaughlan S.; Topisirovic I.; Wang Z.; Wang Z.; Zhao Y.; Wildes D.; Aggen J. B.; Singh M.; Gill A. L.; Smith J. A. M.; Rosen N. Selective inhibitors of mTORC1 activate 4EBP1 and suppress tumor growth. Nat. Chem. Biol. 2021, 17, 1065–1074. 10.1038/s41589-021-00813-7. 34168367 PMC9249104

[ref59] Mahauad-Fernandez W. D.; Yang Y. C.; Lai I.; Park J.; Yao L.; Evans J. W.; Burnett L. G.; Gill A.; Smith J. A. M.; Singh M.; Felsher D. W. Bi-steric mTORC1-selective Inhibitors activate 4EBP1 reversing MYC-induced tumorigenesis and synergize with immunotherapy; https://www.biorxiv.org/content/10.1101/2022.02.04.478208v1.

[ref60] Tibrewal N.; Aggen J. B.; Bassan A. I.; Burnett G. L.; Evans J.; Gliedt M. J.; Hsieh T.; Kiss G.; Lee B. J.; Lee D.; Lorenzana E.; Marquez A.; Thottumkara A.; Wang Z.; Wilson S.; Zhao F.; Goldsmith M. A.; Singh M.; Wildes D.; Gill A. L.; Smith J. A. M. 4EBP1 reactivation by potent and selective bi-steric inhibitors of mTORC1. Proceedings of the AACR Special Conference on Targeting PI3K/mTOR Signaling, 2018 Nov 30–Dec 8; Boston, MA; American Association for Cancer Research: Philadelphia, PA, 2018; Poster PR04.

[ref61] Lee B. J.; Dinglasan N.; Nguyen T.; Lorenzana E. G.; Wilson S. L.; Burnett L. G.; Aggen J. B.; Nichols R. J.; Singh M.; Wildes D.; Smith J. A. M. 4EBP3 mRNA as a biomarker of therapeutic response to treatment with mTORC1 inhibitors. Proceedings of the AACR-NCI-EORTC International Conference on Molecular Targets and Cancer Therapeutics, October 26–30, 2019, Boston, MA; American Association for Cancer Research: Philadelphia, PA, 2019; Poster B108.

[ref62] Yang Y. C.; Jiang J.; Schulze C.; Evans J. W.; Reyes D. F.; Choy T.; Nguyen T.; Wang Z.; Lee D.; Nichols R. J.; Wang Z.; Smith J. A. M.; Kelsey S. M.; Singh M. Positioning a selective, bi-steric inhibitor of mTORC1 as a combination partner in RAS-driven cancers. Proceedings of the Annual Meeting of the American Association for Cancer Research 2020, Virtual Meeting II, June 22–24, 2020; American Association for Cancer Research: Philadelphia, PA, 2020; Poster LB-113.

[ref63] Burnett G. L.; Pitzen J.; Gliedt M.; Semko C. M.; Jiang J.; Yang Y. C.; Schulze C. J.; Marquez A.; Evans J. W.; Wilson S. L.; Hsieh T.; Wang Z. C.; Lee B. J.; Choy T.; Reyes D. F.; Zhao Y.; Tao J. Y.; Du H.; Ozawa T.; Fan Q.; Luo K.; Kiss G.; Wildes D. P.; Raleigh D.; Wang Z. P.; Monga S. P.; Kwiatkowski D. J.; Weiss W. A.; Aggen J.; Singh M.; Smith J. A. M.; Gill A. Discovery of RMC-5552, a selective bi-steric inhibitor of mTORC1 that suppresses 4EBP1 phosphorylation, for the treatment of mTORC1-activated tumors including RAS pathway escape. Proceedings of the Annual Meeting of the American Association for Cancer Research 2021, Virtual Meeting I, Apr 10–15, 2021; American Association for Cancer Research: Philadelphia, PA, 2021; Presentation ND10.

[ref64] Mahauad-Fernandez W.; Yang Y. C.; Lai I.; Park J.; Evans J. W.; Singh M.; Smith J. A. M.; Felsher D. A bi-steric mTORC1 inhibitor that selectively reactivates 4EBP1 and induces regression of MYC-driven hepatocellular carcinoma. Proceedings of the Annual Meeting of the American Association for Cancer Research 2021, Virtual Meeting I, Apr 10–15, 2021; American Association for Cancer Research: Philadelphia, PA, 2021; Poster 1002.

[ref65] Du H.; Yang Y. C.; Singh M.; Liu H.; Kwiatkowski D. J. Bi-steric mTORC1-selective inhibitors demonstrate improved potency and efficacy in tumors with mTORC1 hyperactivation. Proceedings of the Annual Meeting of the American Association for Cancer Research 2021, Virtual Meeting I, Apr 10–15, 2021; American Association for Cancer Research: Philadelphia, PA, 2021; Poster 1026.

[ref66] Burris III H. A.; Ulahannan S. V.; Haura E. B.; Ou S.-H. I.; Capasso A.; Munster P. N.; Kitai H.; Wang Z.; Hayes J.; Tao L.; Wong S.; Yang Y. C.; Jiang J.; Bitman B.; Singh M.; Gustafson W. C.; Rosen N.; Schram A. M. The bi-steric mTORC1-selective inhibitor RMC-5552 in tumors with activation of mTOR signaling: preclinical activity in combination with RAS(ON) inhibitors in RAS-addicted tumors, and initial clinical findings from a single agent phase 1/1b study. J. Clin. Oncol. 2022, 40 (16), 3098 10.1200/JCO.2022.40.16_suppl.3098.36070625

[ref67] Chandarlapaty S.; Sawai A.; Scaltriti M.; Rodrik-Outmezguine V.; Grbovic-Huezo O.; Serra V.; Majumder P. K.; Baselga J.; Rosen N. AKT inhibition relieves feedback suppression of receptor tyrosine kinase expression and activity. Cancer Cell 2011, 19, 58–71. 10.1016/j.ccr.2010.10.031. 21215704 PMC3025058

[ref68] Muranen T.; Selfors L. M.; Worster D. T.; Iwanicki M. P.; Song L.; Morales F. C.; Gao S.; Mills G. B.; Brugge J. S. Inhibition of PI3K/mTOR leads to adaptive resistance in matrix-attached cancer cells. Cancer Cell 2012, 21, 227–239. 10.1016/j.ccr.2011.12.024. 22340595 PMC3297962

[ref69] Rodrik-Outmezguine V. S.; Chandarlapaty S.; Pagano N. C.; Poulikakos P. I.; Scaltriti M.; Moskatel E.; Baselga J.; Guichard S.; Rosen N. mTOR kinase inhibition causes feedback-dependent biphasic regulation of AKT signaling. Cancer Discovery 2011, 1, 248–259. 10.1158/2159-8290.CD-11-0085. 22140653 PMC3227125

[ref70] Serra V.; Scaltriti M.; Prudkin L.; Eichhorn P. J. A.; Ibrahim Y. H.; Chandarlapaty S.; Markman B.; Rodriguez O.; Guzman M.; Rodriguez S.; Gili M.; Russillo M.; Parra J. L.; Singh S.; Arribas J.; Rosen N.; Baselga J. PI3K inhibition results in enhanced HER signaling and acquired ERK dependency in HER2-overexpressing breast cancer. Oncogene 2011, 30, 2547–2557. 10.1038/onc.2010.626. 21278786 PMC3107390

[ref71] Dose escalation of RMC-5552 monotherapy in relapsed/refractory solid tumors; NCT04774952; https://clinicaltrials.gov/ct2/show/NCT04774952.

[ref72] Steffan R. J.; Kearney R. M.; Hu D. C.; Failli A. A.; Skotnicki J. S.; Schiksnis R. A.; Mattes J. F.; Chan K. W.; Caufield C. E. Base catalyzed degradations of rapamycin. Tetrahedron Lett. 1993, 34, 3699–3702. 10.1016/S0040-4039(00)79204-5.

[ref73] Cottens S.; Kallen J.; Schuler W.; Sedrani R. Derivation of rapamycin: adventures in natural product chemistry. Chimia 2019, 73, 581–590. 10.2533/chimia.2019.581. 31431218

[ref74] Il’ichev Y. V.; Alquier L.; Maryanoff C. A. Degradation of rapamycin and its ring-opened isomer: role of base catalysis. ARKIVOC 2007, 2007, 110–131. 10.3998/ark.5550190.0008.c09.

[ref75] Luengo J. I.; Konialian A. L.; Holt D. A. Studies on the chemistry of rapamycin: novel transformations under Lewis-acid catalysis. Tetrahedron Lett. 1993, 34, 991–994. 10.1016/S0040-4039(00)77473-9.

[ref76] Hughes P.; Musser J.; Conklin M.; Russo R. The isolation, synthesis and characterization of an isomeric form of rapamycin. Tetrahedron Lett. 1992, 33, 4739–4742. 10.1016/S0040-4039(00)61273-X.

[ref77] Cottens S.; Sedrani R. O-alkylated rapamycin derivatives and their use, particularly as immunosuppressants. WO9409010, 1994; *Chem Abstr.* **1994**, *122*, 9774.

[ref78] Sedrani R.; Cottens S.; Kallen J.; Schuler W. Chemical modification of rapamycin: the discovery of SDZ RAD. Transplant. Proc. 1998, 30, 2192–2194. 10.1016/S0041-1345(98)00587-9. 9723437

[ref79] Rostovtsev V. V.; Green L. G.; Fokin V. V.; Sharpless K. B. A stepwise Huisgen cycloaddition process: copper(I)-catalyzed regioselective “ligation” of azides and terminal alkynes. Angew. Chem., Int. Ed. 2002, 41, 2596–2599. 10.1002/1521-3773(20020715)41:14<2596::AID-ANIE2596>3.0.CO;2-4. 12203546

[ref80] Hu D. C. Preparation of rapamycin carbonate esters as immunosuppressant agents. U.S. Patent 5,260,300, 1993; *Chem Abstr.* **1993**, *120*, 269937.

[ref81] Luengo J. I.; Rozamus L. W.; Holt D. A. Studies on selective reductions of rapamycin. Tetrahedron Lett. 1994, 35, 6469–6472. 10.1016/S0040-4039(00)78248-7.

[ref82] Nelson F. C. 27-hydroxyrapamycin and derivatives thereof. U.S. Patent 5,256790, 1993; *Chem Abstr.* **1993**, *121*, 9034.

[ref83] Cottens S.; Sedrani R. Synthesis and immunosuppressant activity of rapamycin derivatives. WO9641807, 1996; *Chem Abstr.* **1996**, *126*, 157342.

[ref84] Wang C. P.; Chan K. W.; Schiksnis R. A.; Scatina J.; Sisenwine S. F. High performance liquid chromatographic isolation, spectroscopic characterization, and immunosuppressive activities of two rapamycin degradation products. J. Liq. Chromat. 1994, 17, 3383–3392. 10.1080/10826079408013519.

[ref85] Zhu T. Oxepane isomer of 42-O-(2-hydroxyethyl)rapamycin. U.S. Patent 7,241,771, 2007; *Chem Abstr.* **2006**, *145*, 292763.

[ref86] For clarity, the major structural isomer is shown throughout this publication.

[ref87] Yang H.; Jiang X.; Li B.; Yang H. J.; Miller M.; Yang A.; Dhar A.; Pavletich N. P. Mechanisms of mTORC1 activation by RHEB and inhibition by PRAS40. Nature 2017, 552, 368–373. 10.1038/nature25023. 29236692 PMC5750076

[ref88] Scaiola A.; Mangia F.; Imseng S.; Boehringer D.; Berneiser K.; Shimobayashi M.; Stuttfeld E.; Hall M. N.; Ban N.; Maier T. The 3.2-Å resolution structure of human mTORC2. Sci. Adv. 2020, 6, eabc1251 10.1126/sciadv.abc1251. 33158864 PMC7673708

[ref89] Paine M. F.; Leung L. Y.; Lim H. K.; Liao K.; Oganesian A.; Zhang M.-Y.; Thummel K. E.; Watkins P. B. Identification of a novel route of extraction of sirolimus in human small intestine: roles of metabolism and secretion. J. Pharmacol. Exp. Ther. 2002, 301, 174–186. 10.1124/jpet.301.1.174. 11907172

[ref90] Paine M. F.; Leung L. Y.; Watkins P. B. New insights into drug absorption studies with sirolimus. Ther. Drug Monit. 2004, 26, 463–467. 10.1097/00007691-200410000-00001. 15385826

[ref91] Wang C. P.; Lim H.-K.; Chan K. W.; Scatina J.; Sisenwine S. F. High performance liquid chromatographic isolation and spectroscopic characterization of metabolites from the bile of rats receiving rapamycin (sirolimus) intravenously. J. Liq. Chromat. Rel. Technol. 1997, 20, 1689–1701. 10.1080/10826079708006326.

[ref92] Wang C. P.; Lim H.-K.; Chan K. W.; Scatina J.; Sisenwine S. F. High performance liquid chromatographic isolation and spectroscopic characterization of three major metabolites from the plasma of rats receiving rapamycin (sirolimus) orally. J. Liq. Chromat. 1995, 18, 2559–2568. 10.1080/10826079508009308.

[ref93] Conciatori F.; Ciuffreda L.; Bazzichetto C.; Falcone I.; Pilotto S.; Bria E.; Cognetti F.; Milella M. mTOR cross-talk in cancer and potential for combination therapy. Cancers 2018, 10, 23 10.3390/cancers10010023. 29351204 PMC5789373

[ref94] Misale S.; Fatherree J. P.; Cortez E.; Li C.; Bilton S.; Timonina D.; Myers D. T.; Lee D.; Gomez-Caraballo M.; Greenberg M.; Nangia V.; Greninger P.; Egan R. K.; McClanaghan J.; Stein G. T.; Murchie E.; Zarrinkar P. P.; Janes M. R.; Li L.-S.; Liu Y.; Hata A. N.; Benes C. H. KRAS G12C NSCLC models are sensitive to direct targeting of KRAS in combination with PI3K inhibition. Clin. Cancer Res. 2019, 25, 796–807. 10.1158/1078-0432.CCR-18-0368. 30327306

[ref95] Hallin J.; Engstrom L. D.; Hargis L.; Calinisan A.; Aranda R.; Briere D. M.; Sudhakar N.; Bowcut V.; Baer B. R.; Ballard J. A.; Burkard M. R.; Fell J. B.; Fischer J. P.; Vigers G. P.; Xue Y.; Gatto S.; Fernandez-Banet J.; Pavlicek A.; Velastagui K.; Chao R. C.; Barton J.; Pierobon M.; Baldelli E.; Patricoin E. F. III; Cassidy D. P.; Marx M. A.; Rybkin I. I.; Johnson M. L.; Ou S. I.; Lito P.; Papadopoulos K. P.; Jänne P. A.; Olson P.; Christensen J. G. The KRASG12C inhibitor MRTX849 provides insight toward therapeutic susceptibility of KRAS-mutant cancers in mouse models and patients. Cancer Discovery 2020, 10, 54–71. 10.1158/2159-8290.CD-19-1167. 31658955 PMC6954325

[ref96] Brown W. S.; McDonald P. C.; Nemirovsky O.; Awrey S.; Chafe S. C.; Schaeffer D. F.; Li J.; Renouf D. J.; Stanger B. Z.; Dedhar S. Overcoming adaptive resistance to KRAS and MEK inhibitors by co-targeting mTORC1/2 complexes in pancreatic cancer. Cell Rep. Med. 2020, 1, 100131 10.1016/j.xcrm.2020.100131. 33294856 PMC7691443

[ref97] Blair H. A. Sotorasib: first approval. Drugs 2021, 81, 1573–1579. 10.1007/s40265-021-01574-2. 34357500 PMC8531079

[ref98] Zhang S. S.; Nagasaka M. Spotlight on Sotorasib (AMG 510) for KRASG12C positive non-small cell lung cancer. Lung Cancer: Targets Ther. 2021, 12, 115–122. 10.2147/LCTT.S334623. PMC8504654 34675734

[ref99] Skoulidis F.; Goldberg M. E.; Greenawalt D. M.; Hellmann M. D.; Awad M. M.; Gainor J. F.; Schrock A. B.; Hartmaier R. J.; Trabucco S. E.; Gay L.; Ali S. M.; Elvin J. A.; Singal G.; Ross J. S.; Fabrizio D.; Szabo P. M.; Chang H.; Sasson A.; Srinivasan S.; Kirov S.; Szustakowski J.; Vitazka P.; Edwards R.; Bufill J. A.; Sharma N.; Ou S. I.; Peled N.; Spigel D. R.; Rizvi H.; Aguilar E. J.; Carter B. W.; Erasmus J.; Halpenny D. F.; Plodkowski A. J.; Long N. M.; Nishino M.; Denning W. L.; Galan-Cobo A.; Hamdi H.; Hirz T.; Tong P.; Wang J.; Rodriguez-Canales J.; Villalobos P. A.; Parra E. R.; Kalhor N.; Sholl L. M.; Sauter J. L.; Jungbluth A. A.; Mino-Kenudson M.; Azimi R.; Elamin Y. Y.; Zhang J.; Leonardi G. C.; Jiang F.; Wong K.-K.; Lee J. J.; Papadimitrakopoulou V. A.; Wistuba I. I.; Miller V. A.; Frampton G. M.; Wolchok J. D.; Shaw A. T.; Jänne P. A.; Stephens P. J.; Rudin C. M.; Geese W. J.; Albacker L. A.; Heymach J. V. STK11/LKB1 Mutations and PD-1 Inhibitor Resistance in KRAS -Mutant Lung Adenocarcinoma. Cancer Discovery 2018, 8, 822–835. 10.1158/2159-8290.CD-18-0099. 29773717 PMC6030433

[ref100] Patricelli M. P.; Szardenings A. K.; Liyanage M.; Nomanbhoy T. K.; Wu M.; Weissig H.; Aban A.; Chun D.; Tanner S.; Kozarich J. W. Functional interrogation of the kinome using Nucleotide acyl phosphates. Biochemistry 2007, 46, 350–358. 10.1021/bi062142x. 17209545

[ref101] Bowes J.; Brown A. J.; Hamon J.; Jarolimek W.; Sridhar A.; Waldron G.; Whitebread S. Reducing safety-related drug attrition: the use of in vitro pharmacological profiling. Nat. Rev. Drug Discovery 2012, 11, 909–922. 10.1038/nrd3845. 23197038

