# Supplementary material for: Discovery of RMC-5552, a Selective Bi-Steric Inhibitor of mTORC1, for the Treatment of mTORC1-Activated Tumors
Source: J Med Chem. 2022 Dec 19;66(1):149–69. doi: 10.1021/acs.jmedchem.2c01658 (PMC9841523; doi:10.1021/acs.jmedchem.2c01658)
Supplement: Supplementary file 1 — jm2c01658_si_001.pdf [file jm2c01658_si_001.pdf]

**Supporting Information**

# Discovery of RMC-5552, a Selective Bi-Steric Inhibitor of mTORC1, for the Treatment of mTORC1-Activated Tumors

G. Leslie Burnett,* Yu C. Yang, James B. Aggen, Jennifer Pitzen, Micah K. Gliedt, Chris M. Semko, Abby Marquez, James W. Evans, Gang Wang, Walter S. Won, Aidan C. A. Tomlinson, Gert Kiss, Christos Tzitzilonis, Arun P. Thottumkara, James Cregg, Kevin T. Mellem, Jong S. Choi, Julie C. Lee, Yongyuan Zhao, Bianca J. Lee, Justin G. Meyerowitz, John E. Knox, Jingjing Jiang, Zhican Wang, David Wildes, Zhengping Wang, Mallika Singh, Jacqueline A. M. Smith, Adrian L. Gill*

Revolution Medicines, Incorporated, Redwood City, California, 94063 USA

Corresponding authors:
G. Leslie Burnett, les@revmed.com
Adrian L. Gill, adrian@revmed.com

## Table of Contents

**Experimental Methods** **S3**
**Compound Synthesis and Characterization** **S13**
**Experimental Procedures** **S15**
**NMR Characterization** **S51**
**Table S1.** NMR Analysis of **10** **S51**
**Table S2.** NMR Analysis of **16** **S53**
**Table S3.** NMR Analysis of **18** **S56**

**Table S4.** NMR Analysis of **12** **S59**

**Table S5.** NMR Analysis of RMC-5552 **38** **S61**

**Figure S1.** HPLC trace for RMC-5552 **38** **S63**

**Figure S2.** HPLC trace for RMC-6272 **40** **S64**

**Table S6.** Data collection, processing, and refinement statistics for **11** structure **S65**

**Table S7.** Data collection, processing, and refinement statistics for **12** structure **S65**

**Figure S3.** The unbiased omit maps (Fo – Fc) of **11** (A) and **12** (B) **S67**

**Figure S4.** Fourier shell correlation of independent half maps of mTORC1-RMC-5552-FKBP12 cryo-EM dataset **S68**

**Table S8.** Nanosyn Lipid Kinase Panel Data **S68**

**Table S9.** ActivX KiNativ Kinase Panel Data for RMC-5552 **38** and RMC-6272 **40** in MCF-7 Cells **S68**

**Table S10.** Eurofins Safety Screen[44] Data for RMC-5552 **38** **S77**

**References** **S78**

## Experimental Methods

**Cell culture and reagents.** Cells were obtained from ATCC, grown in appropriate medium supplemented with 10% fetal bovine serum and 1% penicillin/streptomycin and maintained at 37 °C in a humidified incubator at 5% $CO_2$.

**AlphaLISA and MesoScale Discovery (MSD) analysis of 4EBP1, S6K and AKT phosphorylation.** mTOR substrate phosphorylation in MDA-MB-468 cells was assayed using AlphaLISA SureFire Ultra kits for p4EBP1 Thr37/46, p-P70S6K Thr389 and p-AKT1/2/3 Ser473 (PerkinElmer), and MSD Multi-Array Assay Systems for Phospho-4EBP1 (Thr37/46) and Phospho-AKT (Ser473) (MSD). Cells were seeded at 25,000 cells per well and exposed the next day to serial three-fold dilutions of compounds in complete medium with 0.1% dimethylsulfoxide (DMSO). After indicated incubation times, cells were lysed and AlphaLISA or MSD performed according to the manufacturers' protocols. Samples were read using an EnVision Multilabel Plate Reader (PerkinElmer) using standard AlphaLISA settings or using an MSD plate reader. Data were plotted as a function of logM [compound] with a sigmoidal concentration response (variable slope) model fitted to the data to estimate the inhibitor half-maximum effective concentration ($EC_{50}$) in Prism 7 (GraphPad). Alternatively, results were analyzed as percentage of control in which 0% was set as signal from 1 μM Torin1 and 100% was set as the mean of DMSO wells on each plate, and inhibitor $EC_{50}$ was estimated as described above. Both analyses produced consistent $EC_{50}$ values. Cells exposed to mTORC1 bi-steric inhibitors exhibited biphasic curves of AKT phosphorylation: inhibitor $EC_{50}$ was estimated by fitting the response model after excluding normalized mean values >130%, and setting the hill slope >−5. Data are presented as mean values with 95% confidence intervals.

**Determination of FKBP12 binding affinity.** FKBP12 binding affinity was determined by Proteros biostructures GmbH, using a fluorescent probe displacement assay. An FKBP12-binding fluorescent probe consisting of Cy5 modified rapamycin was synthesized by Proteros, and its affinity for FKBP12 was determined to be 0.6 nM by fluorescence titration of 0.74 nM GST-FKBP12 bound to a Cy3-labeled anti-GST antibody in 20 mM MOPS pH 7.0, 1 mM DTT, 0.01% Tween20. FKBP12 binding affinity for unlabeled test articles was determined under the same conditions, with 6 nM fluorescent probe present. 50 nL compound in DMSO was added to 50 µL FKBP12 with probe in a 384 well black microtiter plate. 11 different concentrations of compound with a 3-fold dilution factor were tested. After a 6 h incubation at room temperature acceptor fluorescence was read on a plate reader. $IC_{50}$ values (corresponding to 50% probe displacement) were calculated using standard fitting algorithms. The reporter probe is used at a concentration reflecting the tenfold concentration of its own Kd (probe) value. Thus, according to the Cheng Prusoff equation, the Kd value can be calculated with Kd = $IC_{50}$ / (1 + (([10 x Kd probe]) / [Kd probe]) = $IC_{50}$ / 11.

**TR–FRET ternary complex assay.** Assay was performed by Thermo Fisher Scientific, using a modification of a previously described protocol.[1] A homogeneous assay mixture was prepared using 150 nM recombinant human FKBP12 fused to Emerald GFP (EmGFP), 2 nM GST-tagged human mTOR (residues 1360-2549) and 1 nM LanthaScreen Tb-anti-GST antibody (Rabbit, Thermo Fisher Scientific catalog no. A15112) in 50 mM HEPES pH 7.5, 0.01% polysorbate 20, 1 mM EGTA and 10 mM $MnCl_2$. Compounds were diluted in DMSO, and assay was initiated by mixing 160 nl of compound with 16 µL of assay mixture, resulting in a final DMSO concentration of 1%. Reactions were mixed for 30 s on an orbital shaker and incubated for 1 h at room temperature. The ratio of fluorescence emission at 520 and 495 nm was determined and all

signals were normalized to 1 μM rapamycin (100% complex formation) and no compound (0% complex formation) controls.

**Nanosyn Lipid Kinase screen.** Biochemical inhibition of lipid and related protein kinases was determined by Nanosyn (Santa Clara, CA). Activity of PI3Kα (p110a/p85a) (BPS, lot #110809), PI3Kβ (p110β/p85α) (Carna, lot # 12CBS-0359H), PI3Kδ (p110δ/p85α), (Carna, lot#09CBS-1198D) PI3Kγ (p120γ) (Thermo Fisher, lot#1716438D), and mTORC1 (BPS, lot # 150818-A) was determined by detection of fluorescent substrates using a microfluidic electrophoresis mobility shift assay with a LabChip3000 instrument (Caliper/PerkinElmer). Bodipy labeled phosphatidylinositol 4,5 phosphate (FL-PIP2) (Echelon Biosciences, Salt Lake City, Utah) with 1,2-dioctanoyl-sn-glycero-3-phospho-L-serine (PS) (Avanti Polar Lipids (Alabaster, AL) was used as a substrate for PI3K enzymes and unactive p70S6K was used for mTORC1. The activity of DNA-PK (Promega, Cat#V4106) on a peptide/dsDNA complex was assayed by detection of ADP formation using ADP-Glo (Promega). Kinase reactions were assembled in 384 well plates in a total volume of 20 μL as follows. For PI3K isoforms, the kinase protein was pre-diluted to 2x concentration in the assay buffer (50 mM HEPES, pH 7.5, 1 mM DTT, 5 mM $MgCl_2$, 0.01% CHAPS, 40 mM NaCl, and 10 μM FKBP12), pre-incubated for 30 min and dispensed into 384 well plate (10 μL per well). Compounds were serially pre-diluted in DMSO and added to the protein samples by acoustic dispensing (200 nL, Labcyte Echo). Concentration of DMSO was equalized to 1% in all samples. All test compounds were tested at 12 concentrations, 3x dilution intervals. The control samples (0%-inhibition in the absence of inhibitor, DMSO only) and 100%-inhibition (in the absence of enzyme) were assembled in replicates of four and were used to calculate %-inhibition in the presence of compounds. After 30 min of pre-incubation with compounds, the reactions were initiated by addition of 10 μL of FL-PIP2/PS substrate (10 μM

PS with 1 μM FL-PIP2) supplemented with ATP. Final concentrations of enzymes were: 1 nM (PI3Kα,β), 0.5 nM (PI3Kδ) and 10 nM (PI3Kγ). Final concentration of ATP was 100 μM. Reactions were allowed to proceed for 2.5 h at room temperature. Following incubation, the reactions were quenched by addition of 50 μL of termination buffer (100 mM HEPES, pH 7.5, 0.01% Triton X-100, 20 mM EDTA). Terminated plates were analyzed on a microfluidic electrophoresis instrument (Caliper LabChip® 3000, Caliper Life Sciences/Perkin Elmer). The mTORC1 assay was carried out similarly, but buffer was 100 mM HEPES, pH 7.5, 1 mM DTT, 5 mM $MgCl_2$, 5 mM $MnCl_2$, 0.01% Triton X-100, 0.1% BSA, and 10 μM FKBP12 and substrate consisted of 20 nM un-active p70S6, 2 nM p70S6 substrate peptide, FAM-AKRRRLSSRA and 100 μM ATP. Final concentration of mTORC1 was 0.5 nM and ATP was 33 μM. Activity in each test sample was determined as the product to sum ratio (PSR): P/(S+P), where P is the peak height of the product, and S is the peak height of the substrate. Percent inhibition (Pinh) was determined using the following equation: Pinh = (PSR 0%inh - PSR compound)/(PSR 0%inh - PSR 100%inh )*100 , in which PSR compound is the product/sum ratio in the presence of compound, PSR 0%inh is the product/sum ratio in the absence of compound and the PSR 100%inh is the product/sum ratio in the absence of the enzyme. To determine $IC_{50}$ of compounds the % inh cdata (P inh versus compound concentration) were fitted by a 4 parameter sigmoid concentration response model using XLfit software (IDBS). For DNA-PK, the assay was assembled according to instructions provided by manufacturer of the DNA PK detection kit (Promega, Cat#V4106), except that the assay was performed in the presence of 5 μM FKBP12 and DNA PK was pre-incubated with FKBP12 and compounds for 30 min prior to addition of ATP and peptide/DNA substrate.

**In vitro kinase selectivity panel.** Kinase selectivity and off-target profile were determined using the KiNativ platform and performed at ActivX Biosciences. Briefly, MCF7 cells were exposed to compounds (final concentration 1 μM) for 2 h, lysed, labeled with biotinylated acyl phosphates of ATP and ADP, and analyzed by liquid chromatography–tandem mass spectrometry (LC–MS/MS) using the standard KiNativ protocol as previously described.[2]

**Mouse Pharmacokinetic Studies.** Male Balb/C mice were non-fasted on the day of dosing. PK was evaluated following a single dose via IP administration (3 mg/kg) at Charles River, Worcester, MA, US. Formulation containing transcutol/solutol HS15/$H_2O$ (5/5/90, %v/w/v) was used, with a dose volume at 10 mL/kg. Serial blood samples were collected at 0.5, 1, 2, 4, 6, 8, and 24 h post-dose, and stored on wet ice until processed to plasma by centrifugation (3500 rpm at 5 °C) within 30 min of collection. All plasma samples were transferred into separate 96 well plates and stored at -80 °C until analysis. Quantification of **16** and its seco-product in mouse plasma were conducted using an AB Sciex Triple Quad 6500+ mass spectrometer coupled with a Waters UPLC system. Supernatants were collected by centrifugation after protein precipitation using MeCN containing internal standard (IS, D4-AEA). Supernatant was injected for LC-MS/MS analysis. Analytes were separated using a Waters UPLC BEH C18 (2.1 × 50 mm, 1.7 μm) column. The LC mobile phase (A: 5 mM $NH_4OAc$ in $H_2O$; B: MeCN/MeOH 50/50) gradient was initiated as follows: 0 – 0.10 min, 55% B; 1.40 – 2.50 min, 95% B; 2.55 – 4.00 min, 55% B. The flow rate was 0.6 mL/min. The MRM channels under positive mode were: **16**, m/z 891.6 -> 434.7; seco-product, m/z 900.8 -> 884.5, and D4-AEA (IS), 352.2 -> 66.1. The calibration curve range was 0.5 – 5000 ng/mL.

**Rat Pharmacokinetics Studies.** Male Sprague-Dawley rats were non-fasted on the day of dosing. PK was evaluated following a single dose via IV bolus administration (1 mg/kg) at

Charles River. Formulation containing transcutol/solutol HS15/$H_2O$ (5/5/90, %w/w/v) was used, with a dose volume at 5 mL/kg. Serial blood samples were collected at predose, 0.083, 0.25, 0.5, 1, 2, 4, 6, 8, and 24 h post-dose, and stored on wet ice until processed to plasma by centrifugation (3500 rpm at 5 °C) within 30 min of collection. All plasma samples were transferred into separate 96 well plates and stored at -80 °C until analysis. Quantification of **16** and its seco-product in rat plasma was conducted with the same protocol as described for mouse pharmacokinetics studies above. The calibration curve range was 1.0 – 5000 ng/mL.

**Methods for xenograft studies.** All procedures related to animal handling, care and treatment were performed according to the guidelines by the Institutional Animal Care and Use Committee (IACUC) following the guidance of Association for Assessment and Accreditation of Laboratory Animal Care. Animals were housed per institutional guidelines as determined by the affiliated IACUC, consisting of a typical 12/12 dark/light cycle, ambient temperatures of roughly 18–23 °C with 40–70% humidity.

**MCF-7 xenograft studies.** Female Balb/c nude mice (Shanghai Lingchang Biotechnology), 6–8 weeks of age, were inoculated subcutaneously in the right flank with MCF-7 (ECACC-86012803) cells in 0.2 mL of PBS supplemented with BD Matrigel (1:1) at $10 \times 10^6$ cells per inoculation. Three days prior to inoculation, mice were fed with drinking water containing β-estradiol (40 µg/mL). When mean tumor volume reached between 200–400 $mm^3$, mice were randomized into treatment groups (n = 12/group) for efficacy study, and tumor volume was measured twice weekly by caliper measurements for the duration of the study. RMC-5552 **38** and RMC-6272 **40** were formulated in v/w/v, 5/5/90 Transcutol/Solutol HS 15/$H_2O$, and both compounds were administered by ip injections at indicated doses, once weekly. For single-dose pharmacokinetic/pharmacodynamics (PK/PD) study, mice were randomized into treatment

groups when mean tumor volume reached 400–600 mm$^3$. Tumor and plasma samples were collected for PK and PD analysis at indicated time points after a single dose of indicated treatment. Sapanisertib (1 mg/kg) was administered by ip injection, and everolimus (5 mg/kg) by oral gavage. RMC-5552 **38** and RMC-6272 **40** were administered by ip injections at indicated doses. Everolimus was formulated in v/v/v/, 30/5/65 Propylene glycol/Tween 80/$H_2O$. Sapanisertib was formulated in v/v/v/v, 30/0.5/5/64.5 PEG400/Tween 80/Propylene glycol/$H_2O$.

**NCI-H2122 xenograft studies.** Female Balb/c nude mice (Beijing V.R Biotechnology Co. LTD), 6–8 weeks of age, were inoculated subcutaneously in the right flank with NCI-H2122 (ATCC-CRL-5985) cells in 0.2 ml of PBS supplemented with BD Matrigel (1:1) at $5 \times 10^6$ cells per inoculation. When mean tumor volume reached between 150–200 mm$^3$, mice were randomized into treatment groups (n=10/group) for efficacy study, and tumor volume was measured twice weekly by caliper measurements for the duration of the study. RMC-6272 **40** was administered by ip injections at 10 mg/kg weekly and formulated in v/w/v, 5/5/90 Transcutol/Solutol HS 15/$H_2O$. Sotorasib (AMG 510) was administered by oral gavage at 100 mg/kg daily and formulated in 2% (w/v) HPMC E-50, 0.5% Tween 80 in 50 mM sodium citrate buffer, pH 4.0 ± 0.1.

***In vivo* pharmacodynamics.** Snap frozen tumors collected were homogenized with MSD complete lysis buffer (as prepared according to Meso Scale Discovery kit protocol) using PreCellys Bead Shaker (Bertin Instruments) according to manufacturer's protocol. Phospho-4E-BP1(Thr37/46) Whole Cell Lysate Kit (Meso Scale Discovery) was used to quantify phosphorylation of 4E-BP1 in the tumors according to manufacturer's protocol.

**Expression and purification of FKBP12, FRB domain, and RHEB-GTPγS.** DNA pET28 plasmids containing either FKBP12 (residues 1-108), the FRB domain of mTOR (residues 2018-

2114), or RHEB (residues 1-169) were transformed into *E. coli* BL21 (DE3). The fusion protein was overexpressed and purified by either polyhistidine-affinity chromatography (FKBP12 and RHEB) or glutathione-affinity chromatography (FRB). Tags were removed via TEV protease and a second affinity column was run per sample to remove excess tag and protease. Nucleotide exchange was carried out for RHEB as follows: RHEB was diluted 2X in a buffer consisting of 40 mM Tris, 150 mM $(NH_4)_2SO_4$, 5 mM DTT, 4 μM $ZnCl_2$, at pH 8.0. 5X molar excess GTPγS was added along with 20U alkaline phosphatase per mg protein. This reaction mixture was incubated at 304 K for 4 h. $MgCl_2$ was added to a final concentration of 5 mM. RHEB protein, now loaded with GTPγS, was concentrated to a final volume of 1 mL using a 10 kD Millipore Amicon Ultra-15 centrifugal filter and exchanged into a buffer consisting of 10 mM HEPES, 150 mM NaCl, 1 mM $MgCl_2$, at pH 7.5 using a PD10 column according to manufacturer's instructions.

All proteins were then further purified by size-exclusion chromatography using a SuperDex-75 (Cytiva) column equilibrated with PBS (pH 7.4). Proteins were concentrated to 10 mg/mL.

**Expression and purification of mTORC1 complex.** mTORC1 complex (mTOR-mLST8-RAPTOR) was expressed and purified as previously described.[3] Briefly, an HEK293-F cell line was stably transfected with pcDNA3.1 vectors for FLAG tagged mTOR, mLST8, and RAPTOR. Complex was affinity purified from cell lysate via anti-FLAG agarose beads and the tag was removed by incubation with TEV protease. Additional nickel and anti-FLAG columns removed the protease and cleaved tags, respectively.

The complex was further purified by size-exclusion chromatography using a SuperDex-200 (Cytiva) column equilibrated with 20 mM Tris pH 8.0, 500 mM NaCl, 10 mM DTT, and 10% glycerol and then concentrated to 4.4 mg/mL.

**Crystallization, X-ray data collection, and crystal structure determination.** Crystals of the FKBP12-FRB-11 and FKBP12-FRB-12 ternary complexes were obtained using previously described methods.[4] FKBP12 was incubated with a 3X molar ratio of macrocyclic compound at 277 K overnight. The mixture was 0.22 μm filtered to remove any insoluble compound or precipitated protein. FRB was added up to a 1:1 molar ratio and the complex was incubated for 3 h. Crystals were grown by the hanging-drop method by mixing 1 μL protein and 1 μL of well solution containing 3.0-3.3 M sodium formate and 0.1 M HEPES pH 7.0-7.5. Crystallization plates were stored at 293 K and crystals were harvested after 2 weeks.

Crystals were cryoprotected with mother liquor supplemented with 20% glycerol and vitrified by plunging into liquid nitrogen. X-ray diffraction data have been collected at the SWISS LIGHT SOURCE (SLS, Villigen, Switzerland) at 100 K. The crystals belong to space group $P\ 6_4\ 2\ 2$. Data were processed using XDS and XSCALE.

The phase information necessary to determine and analyze the structure was obtained by molecular replacement using a previously obtained crystal structure. Subsequent model building and refinement was performed according to standard protocols with the software packages CCP4 (REFMAC5 and COOT). The ligand parameterization and generation of the corresponding library files were carried out with CORINA. Statistics of the final structure and the refinement process are listed in **Table S6** and **Table S7**.

**Cryo-EM sample and grid preparation.** Cryo-EM samples were prepared using a crosslinking procedure previously described.[5] Purified mTORC1, RHEB–GTPγS, GTPγS, NaCl, and FKBP-RMC-5552 **38** were mixed to final concentrations of 0.21 μM, 21 μM, 200 μM, 100 mM and

0.42 μM respectively. Crosslinking was carried out with 0.24 mM BS3 for 45 minutes on ice, followed by additional crosslinking in the presence of 0.18% glutaraldehyde in 20 mM bicine, pH 8.0, 260 mM NaCl, 10% glycerol, 5 mM $MgCl_2$, and 0.5 mM TCEP. The reaction was quenched with 100 mM Tris-HCl, pH 8.0, and the mixture was purified by size-exclusion chromatography (Superdex 200) in 20 mM Tris-HCl, pH 8.0, 260 mM NaCl, 5 mM $MgCl_2$, and 0.1 mM TCEP. Peak fractions were concentrated by ultra filtration to 1 mg/mL and were supplemented with 17.5 μM RHEB–GTPγS, 0.2 mM GTPγS, and 0.21 μM FKBP-RMC-5552 **38**.

Cryo grids were prepared with an FEI Vitrobot Mark IV (Thermo Fisher Scientific). A 3 μL sample was applied to glow-discharged UltraAuFoil R1.2/1.3 300 mesh grids (Quantifoil), blotted for 5 s at 8 °C, 100% humidity and plunge-frozen in liquid ethane precooled by liquid nitrogen. The grids were screened on a 200kV Glacios electron microscope (Thermo Fisher Scientific) at 1300x/92000x magnification. Grids showing adequate ice and particle concentration were saved for Krios data collection.

**Cryo-EM data collection, processing, and structure refinement.** Cryo-EM data were collected on a 300kV Titan Krios G3i microscope equipped with a K2 Summit direct electron detector (Gatan) and GIF Quantum energy filter (20 eV slit width) using SerialEM 3.8.6.[6] 12,808 movies were collected with a pixel size of 0.55 Å in super resolution mode at the specimen level, defocus range of -1.0 to -3.0 μm and a total dose of 48 electrons/Å2 fractionated to 40 frames.

Data processing was carried out with Relion3.1.[7] Motion correction was performed using Motioncor2[8] with a binning factor of 2, generating micrographs at a pixel size of 1.1 Å. Contrast transfer function (CTF) parameters were estimated by Kai Zhang's GCTF.[9] 10,649 adequate micrographs without severe ice contaminations were used for subsequent data processing.

1,757,905 particles were semi-automatically picked using a Laplacian-of-Gaussian filter and subject to multiple rounds of 2D/3D classifications to remove poor quality particles. After screening, 805,027 particles were retained for 3D refinement without imposed symmetry, on which CTF refinement and Bayesian particle polishing were performed. 3D refinement with polished particles and C2 symmetry yielded a 3.1 Å reconstruction according to the FSC=0.143 criterion after postprocessing with a soft mask. To further improve the resolution of monomeric form, we computationally split each particle to two monomers by subtracting their signal with a monomer mask, resulting in 1,610,054 pseudo-monomer particles. 3D refinement with these pseudo-monomer particles yielded a 2.9 Å map after postprocessing. Local resolution was estimated by Relion's built-in implementation. The final map was sharpened anisotropically using Phenix LocalAnisoSharpen.

Initial protein models were docked into their corresponding locations using a previously obtained structure in ChimeraX.[5,10] The ligand parameterization and generation of the corresponding library files were carried out with Acedrg.[11] Iterative rounds of model building and refinement were performed by conventional methods using COOT and phenix.refine.[12]

Molecular graphics of maps and models were generated using ChimeraX 1.3[10] and Pymol 2.4.[13]

**Compound Synthesis and Characterization**

All solvents and commercially available reagents were used as received. All reactions were followed by TLC analysis or LCMS. Column chromatography was performed on prepacked silica gel columns (Biotage SNAP KP-Sil) using a Biotage Isolera One system. Reverse phase preparative chromatography was performed on a Uptisphere Strategy C18-HQ 5 µm 150 mm × 7 mm column using an Interchim PuriFlash system. The column was eluted with MeCN/$H_2O$ with

0.1% formic acid. All key compounds were >95% purity by HPLC. The purity for compounds and low resolution mass spectra were determined using liquid chromatography mass spectrometry (LCMS) on a Shimadzu LC-20 instrument using electrospray ionization. LCMS conditions were as follows: Uptisphere Strategy C18-HQ 5 µm 150 × 4.6 mm, 55%→100% MeCN (0.1% TFA) in $H_2O$ (0.1% TFA), 20 min run, oven temperature 60 °C, flow rate 0.5 mL/min, UV detection (λ = 280 nm). HRMS were performed on a Thermo Fisher LTQ Orbitrap using high-performance liquid chromatography with electrospray ionization Orbitrap mass spectrometry (HPLC ESI Orbitrap/MS). Liquid-state $^{1}H$ NMR experiments for intermediates were recorded on 400, 500, or 600 MHz Bruker Avance III NMR spectrometers. Liquid-state $^{1}H$, COSY, $^{13}C$, and HMBC spectra were recorded on a 600 MHz Bruker Avance III NMR spectrometer (600 MHz for $^{1}H$, 151 MHz for $^{13}C$) using a triple-resonance $^{1}H$,$^{15}N$,$^{13}C$ CP-TCI 5 mm cryoprobe (Bruker Biospin, Germany). All of the experiments used for the resonance assignment procedure and the elucidation of the final products structures (1D $^{1}H$, 1D $^{13}C$, 2D $^{1}H$-$^{1}H$-COSY, 2D $^{1}H$-$^{1}H$-TOCSY, 2D $^{1}H$-$^{1}H$-ROESY, 2D $^{1}H$-$^{13}C$-HSQC, 2D $^{1}H$-$^{13}C$-HMBC) were recorded at 300 K. $^{1}H$ chemical shifts are reported in δ (ppm) as s (singlet), d (doublet), t (triplet), q (quartet), dd (double doublet), m (multiplet), br s (broad singlet), and o (overlay) and are referenced to TMS as an internal standard.

**Experimental Procedures**

**(3*S*,6*R*,7*E*,9*R*,10*R*,12*R*,14*S*,15*E*,17*E*,19*E*,21*S*,23*S*,26*R*,27*R*,34a*S*)-3-((*R*)-1-((1*S*,3*R*,4*R*)-4-(hex-5-yn-1-yloxy)-3-methoxycyclohexyl)propan-2-yl)-9,27-dihydroxy-10,21-dimethoxy-6,8,12,14,20,26-hexamethyl-9,10,12,13,14,21,22,23,24,25,26,27,32,33,34,34a-hexadecahydro-3*H*-23,27-epoxypyrido[2,1-*c*][1]oxa[4]azacyclohentriacontine-1,5,11,28,29(4*H*,6*H*,31*H*)-pentaone (10)**

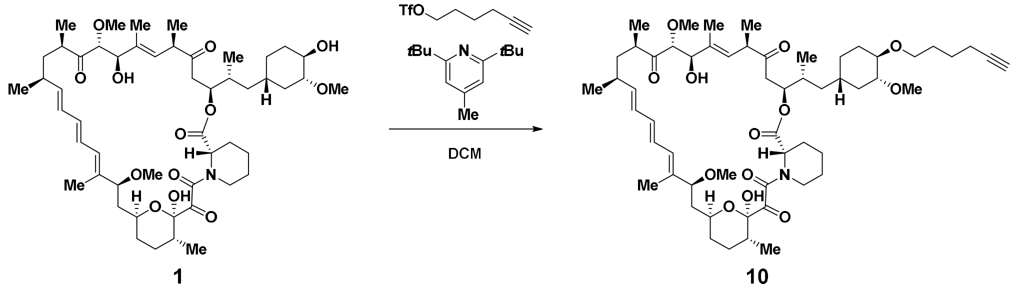


To a solution of freshly purified hex-5-yn-1-yl trifluoromethanesulfonate (5.09 g, 22.1 mmol, 4.0 equiv) in DCM (25 mL) at 0 °C was added 2,6-di-*tert*-butyl-4-methylpyridine (2.26 g, 11.0 mmol, 2.0 equiv) in one portion. The mixture was stirred for 5 min and then rapamycin **1** (5.00 g, 5.47 mmol, 1.0 equiv) was added. The reaction mixture was stirred at 0 °C for 1 h and was then warmed to room temperature and stirred overnight. The reaction mixture was diluted with DCM (100 mL) and quenched with diethylamine (2.75 mL, 26.6 mmol, 4.9 equiv). The solution was washed with sat. aqueous $NaHCO_3$ (100 mL) and brine (100 mL). The aqueous layers were each extracted with DCM (10 mL) and the combined organic layers were dried over $MgSO_4$, filtered, and concentrated under reduced pressure. Purification by silica gel chromatography (45% EtOAc/hexanes) followed by a second purification by silica gel chromatography (0→35% EtOAc/hexanes) afforded the desired product (1.67 g, 30% yield) as a

colorless amorphous solid. LCMS (ESI) *m/z*: [M + Na] calcd for $C_{57}H_{87}NO_{13}$: 1016.61; found 1016.5

**(3*S*,5*R*,6*R*,7*E*,9*R*,10*R*,12*R*,14*S*,15*E*,17*E*,19*E*,21*S*,23*S*,26*R*,27*R*,34a*S*)-5,9,27-trihydroxy-3-((*R*)-1-((1*S*,3*R*,4*R*)-4-hydroxy-3-methoxycyclohexyl)propan-2-yl)-10,21-dimethoxy-6,8,12,14,20,26-hexamethyl-5,6,9,10,12,13,14,21,22,23,24,25,26,27,32,33,34,34a-octadecahydro-3*H*-23,27-epoxypyrido[2,1-*c*][1]oxa[4]azacyclohentriacontine-1,11,28,29(4*H*,31*H*)-tetraone (11)**

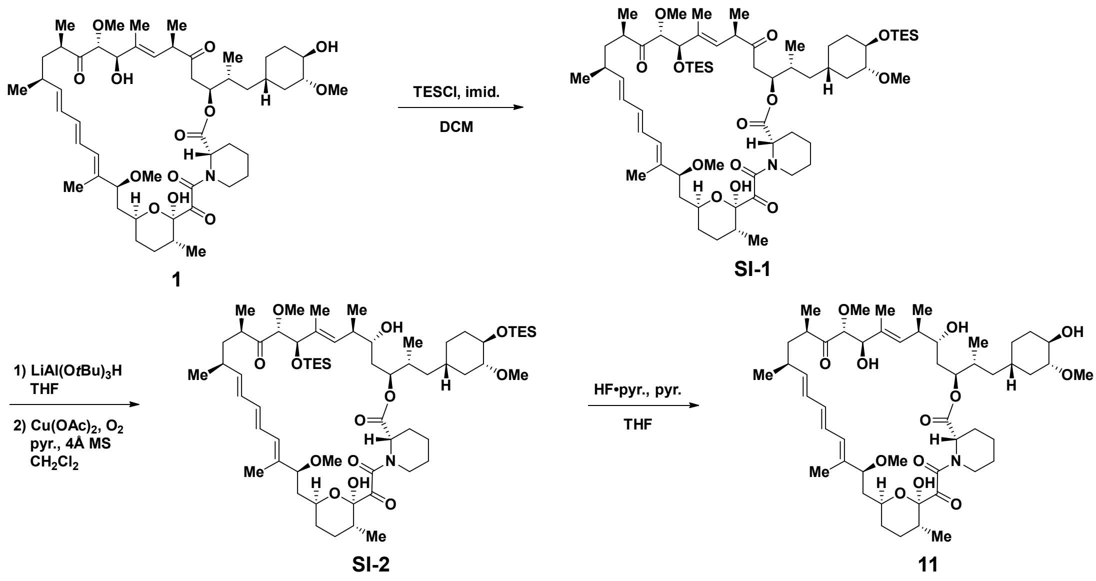


***Step 1*: Synthesis of (3*S*,6*R*,7*E*,9*R*,10*R*,12*R*,14*S*,15*E*,17*E*,19*E*,21*S*,23*S*,26*R*,27*R*,34a*S*)-27-hydroxy-10,21-dimethoxy-3-((*R*)-1-((1*S*,3*R*,4*R*)-3-methoxy-4-((triethylsilyl)oxy)cyclohexyl)propan-2-yl)-6,8,12,14,20,26-hexamethyl-9-((triethylsilyl)oxy)-9,10,12,13,14,21,22,23,24,25,26,27,32,33,34,34a-hexadecahydro-3*H*-23,27-epoxypyrido[2,1-*c*][1]oxa[4]azacyclohentriacontine-1,5,11,28,29(4*H*,6*H*,31*H*)-pentaone (SI-1)**

To a solution of rapamycin **1** (124.4 g, 136.1 mmol, 1 equiv) in DCM (1.87 L) at 0 °C was added imidazole (28.0 g, 411 mmol, 3.0 equiv) followed by chlorotriethylsilane (64 mL, 381 mmol, 2.8 equiv), dropwise. The reaction mixture was stirred at 0 °C for 30 min and was then warmed to room temperature. After an additional 30 min the reaction mixture was filtered through celite and washed with DCM (900 mL). The filtrate was washed with $H_2O$ (930 mL), sat. $NaHCO_3$ (930 mL), dried over $MgSO_4$, and concentrated under reduced pressure to afford the crude desired product (165 g, 79% yield) as a colorless amorphous solid.

***Step 2*****: Synthesis of (3*****S*****,5*****R*****,6*****R*****,7*****E*****,9*****R*****,10*****R*****,12*****R*****,14*****S*****,15*****E*****,17*****E*****,19*****E*****,21*****S*****,23*****S*****,26*****R*****,27*****R*****,34a*****S*****)-5,27-dihydroxy-10,21-dimethoxy-3-((*****R*****)-1-((1*****S*****,3*****R*****,4*****R*****)-3-methoxy-4-((triethylsilyl)oxy)cyclohexyl)propan-2-yl)-6,8,12,14,20,26-hexamethyl-9-((triethylsilyl)oxy)-5,6,9,10,12,13,14,21,22,23,24,25,26,27,32,33,34,34a-octadecahydro-3*****H*****-23,27-epoxypyrido[2,1-*****c*****][1]oxa[4]azacyclohentriacontine-1,11,28,29(4*****H*****,31*****H*****)-tetraone (SI-2)**

To a solution of **SI-1** (109.6 g, 85.36 mmol, 1.0 equiv) in THF (1.42 L) at -30 °C was added 1.1 M of lithium tri-*tert*-butoxyaluminum hydride in THF (194.0 mL, 213.4 mmol, 2.5 equiv). The reaction mixture was then warmed to 0 °C and was stirred for 4 h. The reaction mixture was then poured into EtOAc (710 mL) and a solution of citric acid in $H_2O$ (2M, 710 mL) at 0 °C. The mixture was stirred for 5 min and then the layers were separated. The aqueous layer was extracted with EtOAc (2 x 250 mL), and the combined organic layers were neutralized with sat. $NaHCO_3$. The organic layer was washed with brine (250 mL), dried over $MgSO_4$, and concentrated under reduced pressure. The residue was dissolved in DCM (560 mL) at room temperature and freshly dried 4Å molecular sieves were added followed by pyridine (11.4 mL, 141 mmol, 1.7 equiv) and cupric acetate (12.8 g, 70.3 mmol, 0.8 equiv). Oxygen was bubbled into the reaction mixture from a balloon and after 1 h the mixture was filtered through celite and

washed with DCM (2 x 250 mL). The filtrate was washed with $H_2O$ (200 mL), brine (200 mL), dried over $MgSO_4$, and concentrated under reduced pressure. Purification of the crude material by silica gel chromatography (5→20% EtOAc/hexanes) afforded the desired product (53.1 g, 54% yield) as a colorless amorphous solid. LCMS (ESI) *m/z*: [M – TES + Na] calcd for $C_{63}H_{109}NO_{13}Si_2$: 1052.65; found 1053.0.

***Step 3*: Synthesis of (3*S*,5*R*,6*R*,7*E*,9*R*,10*R*,12*R*,14*S*,15*E*,17*E*,19*E*,21*S*,23*S*,26*R*,27*R*,34a*S*)-5,9,27-trihydroxy-3-((*R*)-1-((1*S*,3*R*,4*R*)-4-hydroxy-3-methoxycyclohexyl)propan-2-yl)-10,21-dimethoxy-6,8,12,14,20,26-hexamethyl-5,6,9,10,12,13,14,21,22,23,24,25,26,27,32,33,34,34a-octadecahydro-3*H*-23,27-epoxypyrido[2,1-*c*][1]oxa[4]azacyclohentriacontine-1,11,28,29(4*H*,31*H*)-tetraone (11)**

To a solution of **SI-2** (3.64 g, 3.18 mmol, 1 equiv) in THF (41.8 mL) was added pyridine (20.8 mL, 258 mmol, 81 equiv) and the reaction mixture was cooled to 0 °C. The solution was treated dropwise with 70% HF-pyridine (4.60 mL, 159 mmol, 50 equiv) and the reaction mixture was stirred at 0 °C for 20 min followed by warming to room temperature. After 5 h, the reaction mixture was cooled to 0 °C and carefully added to an ice cold sat. $NaHCO_3$ solution (400 mL). The mixture was extracted with EtOAc (2 x 100 mL), and the organic phases were washed with $H_2O$ (75 mL), sat. $NaHCO_3$ (75 mL), and brine (75 mL). The organic solution was dried over $Na_2SO_4$, filtered, and concentrated under reduced pressure. The crude material was purified by silica gel chromatography (20→40% acetone/hexanes) to afford the desired product (1.66 g, 57% yield) as a colorless amorphous solid. LCMS (ESI) *m/z*: [M + Na] calcd for $C_{51}H_{81}NO_{13}$: 938.56; found 938.7; *m/z*: [M - H] calcd for $C_{51}H_{81}NO_{13}$: 914.56; found 914.7.

**(3*S*,5*R*,6*R*,7*E*,9*R*,10*R*,12*R*,14*S*,15*E*,17*E*,19*E*,21*S*,23*S*,26*R*,27*R*,34a*S*)-9,27-dihydroxy-3-((*R*)-1-((1*S*,3*R*,4*R*)-4-hydroxy-3-methoxycyclohexyl)propan-2-yl)-5,10,21-trimethoxy-6,8,1214,20,26-hexamethyl-5,6,9,10,12,13,14,21,22,23,24,25,26,27,32,33,34,34a-octadecahydro-3*H*-23,27-epoxypyrido[2,1-*c*][1]oxa[4]azacyclohentriacontine-1,11,28,29(4*H*,31*H*)-tetraone (12)**

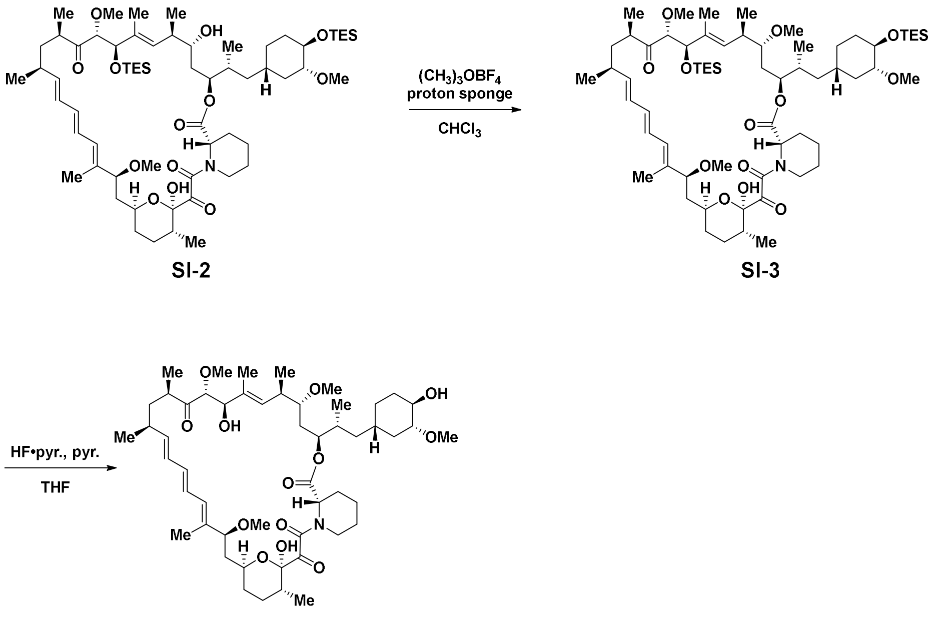


12

***Step 1*: Synthesis of (3*S*,5*R*,6*R*,7*E*,9*R*,10*R*,12*R*,14*S*,15*E*,17*E*,19*E*,21*S*,23*S*,26*R*,27*R*,34a*S*)-27-hydroxy-5,10,21-trimethoxy-3-((*R*)-1-((1*S*,3*R*,4*R*)-3-methoxy-4-((triethylsilyl)oxy)cyclohexyl)propan-2-yl)-6,8,12,14,20,26-hexamethyl-9-((triethylsilyl)oxy)-5,6,9,10,12,13,14,21,22,23,24,25,26,27,32,33,34,34a-octadecahydro-3*H*-23,27-epoxypyrido[2,1-*c*][1]oxa[4]azacyclohentriacontine-1,11,28,29(4*H*,31*H*)-tetraone (SI-3)**

To a stirred solution of **SI-2** (3.83 g, 3.34 mmol, 1.0 equiv) in $CHCl_3$ (95.8 mL) was added Proton Sponge® (7.17 g, 33.5 mmol, 10.0 equiv) along with freshly dried 4 Å molecular sieves (4 g). The solution was stirred for 1 h prior to the addition of trimethyloxonium

tetrafluoroborate (4.95 g, 33.5 mmol, 10.0 equiv, dried by heating under reduced pressure at 50 °C for 1 h before use) at room temperature. The reaction mixture was stirred for 18 h, and was then diluted with DCM and filtered through Celite. The filtrate was washed with 1 M HCl (2x) and sat. $NaHCO_3$ and was then concentrated under reduced pressure. The residue was purified by silica gel chromatography (10→20% EtOAc/hexanes) and the resulting residue was then taken up in MTBE and washed with 1 M HCl, sat. $NaHCO_3$, dried over $Na_2SO_4$, filtered, and concentrated under reduced pressure to afford the desired product (3.15 g, 81% yield) as a colorless amorphous solid. LCMS (ESI) *m/z*: [M – TES + $H_2O$] calcd for $C_{64}H_{111}NO_{13}Si_2$: 1061.68; found 1061.9.

***Step 2*: Synthesis of (3*S*,5*R*,6*R*,7*E*,9*R*,10*R*,12*R*,14*S*,15*E*,17*E*,19*E*,21*S*,23*S*,26*R*,27*R*,34a*S*)-9,27-dihydroxy-3-((*R*)-1-((1*S*,3*R*,4*R*)-4-hydroxy-3-methoxycyclohexyl)propan-2-yl)-5,10,21-trimethoxy-6,8,1214,20,26-hexamethyl-5,6,9,10,12,13,14,21,22,23,24,25,26,27,32,33,34,34a-octadecahydro-3*H*-23,27-epoxypyrido[2,1-*c*][1]oxa[4]azacyclohentriacontine-1,11,28,29(4*H*,31*H*)-tetraone (12)**

To a stirred solution of **SI-3** (1.11 g, 0.958 mmol, 1.0 equiv) in THF (12.6 mL) and pyridine (6.30 mL) at 0 °C in a plastic vial was added 70% HF-pyridine (2.22 mL, 76.6 mmol, 80.0 equiv), dropwise. The reaction mixture was stirred at 0 °C for 20 min before being warmed to room temperature for 3 h. The reaction mixture was cooled to 0 °C and poured slowly into ice cold sat. $NaHCO_3$ (50 mL). The aqueous layer was extracted with EtOAc (3x), and the combined organics were washed with sat. $NaHCO_3$, brine, dried, filtered, and concentrated under reduced pressure. The residue was dissolved in MeOH (5 mL) and added dropwise to $H_2O$ (50 mL) to produce a precipitate. After stirring for 15 min the slurry was filtered and the cake washed with $H_2O$ (2x). The crude product was then dissolved in MeCN (50 mL) and lyophilized overnight to

afford the desired product (780 mg, 87% yield) as a colorless amorphous solid. LCMS (ESI) *m/z*: [M + Na] calcd for $C_{52}H_{83}NO_{13}$: 952.58; found 952.4.

**(1*R*,2*R*,4*S*)-4-((*R*)-2-((3*S*,6*R*,7*E*,9*R*,10*R*,12*R*,14*S*,15*E*,17*E*,19*E*,21*S*,23*S*,26*R*,27*R*,34a*S*)-9,27-dihydroxy-10,21-dimethoxy-6,8,12,14,20,26-hexamethyl-1,5,11,28,29-pentaoxo-1,4,5,6,9,10,11,12,13,14,21,22,23,24,25,26,27,28,29,31,32,33,34,34a-tetracosahydro-3*H*-23,27-epoxypyrido[2,1-*c*][1]oxa[4]azacyclohentriacontin-3-yl)propyl)-2-methoxycyclohexyl (4-nitrophenyl) carbonate (13)**

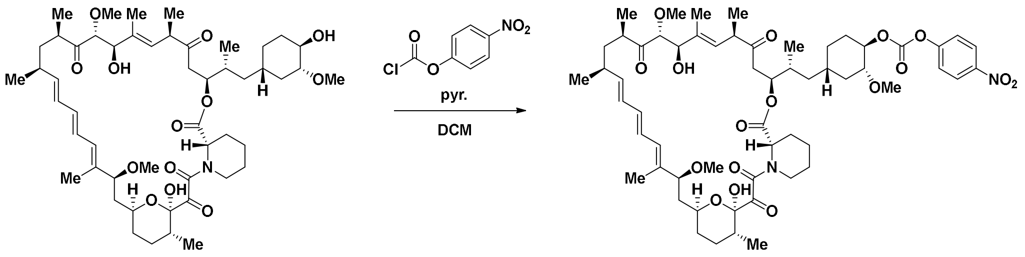


1 13

To a solution of rapamycin **1** (30.10 g, 32.92 mmol, 1.0 equiv) in DCM (148.9 mL) was added pyridine (29.6 mL, 367 mmol, 11.1 equiv). The solution was cooled to -78 °C and then *p*-nitrophenyl chloroformate (12.48 g, 61.92 mmol, 1.9 equiv) was added. The reaction was stirred at -78 °C for 2 h. To the reaction mixture was then added DCM and the solution was then poured into $H_2O$. The aqueous layer was extracted with DCM and the combined organic layers were dried over $MgSO_4$ and concentrated under reduced pressure. The crude material was purified by silica gel chromatography (0→50% EtOAc/hexanes) to afford the desired product (23.1 g, 59% yield) as a colorless amorphous solid. LCMS (ESI) *m/z*: [M + Na] calcd for $C_{58}H_{82}N_2O_{17}$: 1101.55; found 1101.6.

**(1*R*,2*R*,4*S*)-2-methoxy-4-((*R*)-2-((3*S*,5*R*,6*R*,7*E*,9*R*,10*R*,12*R*,14*S*,15*E*,17*E*,19*E*,21*S*,23*S*,26*R*,27*R*,34a*S*)-5,9,27-trihydroxy-10,21-dimethoxy-6,8,12,14,20,26-hexamethyl-1,11,28,29-tetraoxo-1,4,5,6,9,10,11,12,13,14,21,22,23,24,25,26,27,28,29,31,32,33,34,34a-tetracosahydro-3*H*-23,27-epoxypyrido[2,1-*c*][1]oxa[4]azacyclohentriacontin-3-yl)propyl)cyclohexyl (4-nitrophenyl) carbonate (14)**

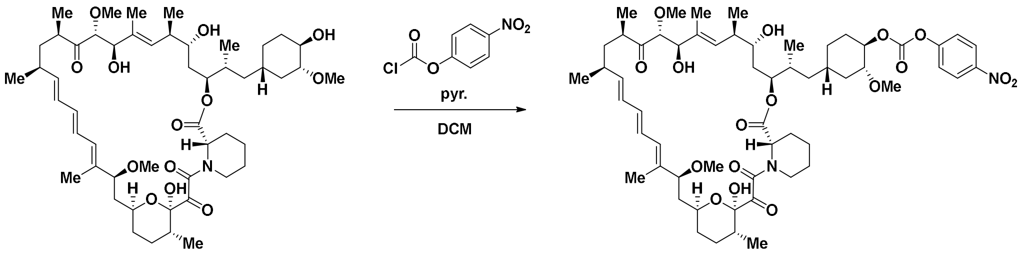


11 14

To a suspension of powdered 4Å molecular sieves (6.0 g) in DCM (130 mL) was added **11** (6.00 g, 6.55 mmol, 1.0 equiv). After stirring at room temperature for 45 min, pyridine (5.99 mL, 74.0 mmol, 11.3 equiv) was added. The suspension was cooled to -15 °C and then 4-nitrophenylchloroformate (1.78 g, 8.84 mmol, 1.4 equiv) was then added. The reaction mixture was stirred at -10 °C for 2 h and then filtered, and the filter pad washed with DCM (140 mL). The filtrate was washed with sat. $NaHCO_3$ (130 mL), $H_2O$ (130 mL) and brine (130 mL), dried over $Na_2SO_4$, filtered, and concentrated under reduced pressure. The crude material was purified by silica gel chromatography (20→50% EtOAc/hexanes) to afford the desired product (4.44 g, 63% yield) as an off-white stiff foam. LCMS (ESI) *m/z*: [M + Na] calcd for $C_{58}H_{84}N_2O_{17}$: 1103.57; found 1103.5.

**(1*R*,2*R*,4*S*)-4-((*R*)-2-((3*S*,5*R*,6*R*,7*E*,9*R*,10*R*,12*R*,14*S*,15*E*,17*E*,19*E*,21*S*,23*S*,26*R*,27*R*,34a*S*)-9,27-dihydroxy-5,10,21-trimethoxy-6,8,12,14,20,26-hexamethyl-1,11,28,29-tetraoxo-1,4,5,6,9,10,11,12,13,14,21,22,23,24,25,26,27,28,29,31,32,33,34,34a-tetracosahydro-3*H*-23,27-epoxypyrido[2,1-*c*][1]oxa[4]azacyclohentriacontin-3-yl)propyl)-2-methoxycyclohexyl (4-nitrophenyl) carbonate (15)**

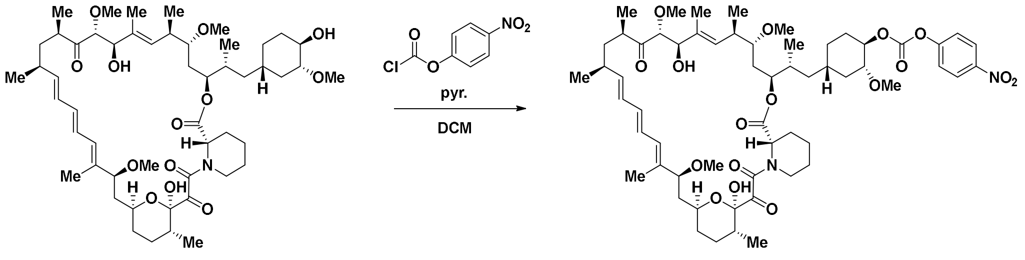


To a solution of **12** (4.50 g, 4.84 mmol, 1.0 equiv) in DCM (180 mL) was added powdered 4Å molecular sieves (6.0 g). The mixture was stirred at room temperature for 1 h and then pyridine (3.91 mL, 48.4 mmol, 10 equiv) was added. The mixture was cooled to -10 °C and 4-nitrophenylchloroformate (0.990 g, 4.91 mmol, 1.0 equiv) was added in one portion. The reaction was allowed to slowly warm to room temperature and after 3 h the reaction mixture was cooled to 0 °C and 4-nitrophenylchloroformate (250 mg, 1.24 mmol, 0.3 equiv) was added. The mixture was warmed to room temperature and after 1 h the reaction mixture was filtered through a pad of celite and the pad was washed with DCM (140 mL). The filtrate was washed with $H_2O$ (120 mL) and sat $NaHCO_3$ (2 x 120 mL). The organic phase was dried over $Na_2SO_4$, filtered, and concentrated under reduced pressure. The crude material was purified by flash chromatography (20→50% EtOAc/hex) to yield a white stiff foam. The material was taken up in MeCN during which time a white solid formed. The solid was filtered, washed with additional MeCN and allowed to air dry to afford the desired product (4.51 g, 85% yield) as an off-white stiff foam. LCMS (ESI) *m/z* [M + Na] calcd for $C_{59}H_{86}N_2O_{17}$: 1117.58; found 1117.6.

**5-(4-amino-1-(4-aminobutyl)-1*H*-pyrazolo[3,4-*d*]pyrimidin-3-yl)benzo[*d*]oxazol-2-amine (SI-6)**

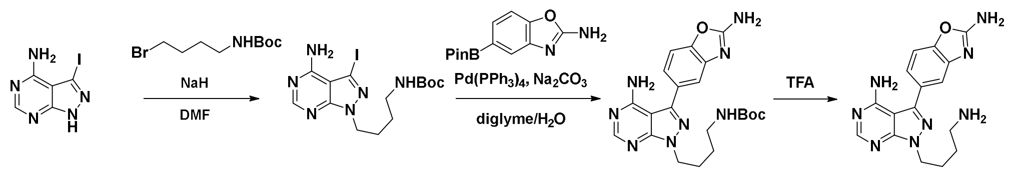


SI-4 SI-5 SI-6

***Step 1*: *tert*-butyl (4-(4-amino-3-iodo-1*H*-pyrazolo[3,4-*d*]pyrimidin-1-yl)butyl)carbamate (SI-4)**

To a suspension of 3-iodo-1*H*-pyrazolo[3,4-*d*]pyrimidin-4-amine (15.0 g, 57.5 mmol, 1.0 equiv) in DMF (50 mL) was added NaH (4.6 g, 115 mmol, 60 wt.%, 2.0 equiv) at 0 °C. The mixture was stirred at 0 °C for 30 min and then a solution of *tert*-butyl *N*-(4-bromobutyl)carbamate (14.14 mL, 69.76 mmol, 1.2 equiv) in DMF (10 mL) was added. The mixture was stirred at room temperature for 14 h. The mixture was then added to $H_2O$ (130 mL), cooled to 0 °C and stirred for 30 min. The resulting precipitate was collected by filtration to give the crude product. Purification by silica gel chromatography (0→20% EtOAc/pet. ether) afforded the desired product (9.35 g, 38% yield) as white solid. LCMS (ESI) *m/z*: [M + H] calcd for $C_{14}H_{21}IN_6O_2$: 433.08; found 433.0. $^1$H NMR (400 MHz, DMSO-$d_6$) δ 8.19 (s, 1H), 6.81 (br t, $J$ = 5.4 Hz, 1H), 4.25 (br t, $J$ = 6.8 Hz, 2H), 2.96-2.77 (m, 2H), 1.80-1.68 (m, 2H), 1.39-1.21 (m, 11H).

***Step 2*: *tert*-butyl (4-(4-amino-3-(2-aminobenzo[*d*]oxazol-5-yl)-1*H*-pyrazolo[3,4-*d*]pyrimidin-1-yl)butyl)carbamate (SI-5)**

To a bi-phasic suspension of **SI-4** (8.80 g, 20.4 mmol, 1.0 equiv), 5-(4,4,5,5-tetramethyl-1,3,2-dioxaborolan-2-yl)benzo[*d*]oxazol-2-amine (7.94 g, 30.5 mmol, 1.5 equiv), and $Na_2CO_3$ (10.79 g, 101 mmol, 5.0 equiv) in 1-methoxy-2-(2-methoxyethoxy)ethane (180 mL) and $H_2O$ (90 mL) was added $Pd(PPh_3)_4$ (2.35 g, 2.03 mmol, 0.1 equiv). The mixture was stirred at 110 °C for 3 h. The reaction mixture was then cooled to room temperature and partitioned between EtOAc (1000 mL) and $H_2O$ (600 mL). The aqueous layer was extracted with EtOAc and the combined organic layers were washed with brine, dried, and concentrated under reduced pressure. Purification by silica gel column chromatography (50% EtOAc/hexanes then 20% MeOH/EtOAc) afforded the desired product (7.30 g, 82% yield) as a light brown solid. LCMS (ESI) *m/z*: [M + H] calcd for $C_{21}H_{26}N_8O_3$: 439.22; found 439.2. $^1$H NMR (400 MHz, DMSO-$d_6$) δ 8.23 (s, 1H), 7.53 (s, 2H), 7.46 (d, *J* = 8.2 Hz, 1H), 7.41 (s, 1H), 7.24 (br d, *J* = 8.0 Hz, 1H), 6.79 (br s, 1H), 4.32 (br t, *J* = 6.6 Hz, 2H), 2.93 (br d, *J* = 5.6 Hz, 2H), 1.88-1.76 (m, 2H), 1.35 (m, 11H).

***Step 3*: 5-(4-amino-1-(4-aminobutyl)-1*H*-pyrazolo[3,4-*d*]pyrimidin-3-yl)benzo[*d*]oxazol-2-amine (SI-6)**

To TFA (78.2 mL, 1.02 mol, 61.1 equiv) was added **SI-5** (7.30 g, 16.7 mmol, 1.0 equiv) at 0 °C. The mixture was warmed to room temperature and stirred for 1 h. The solution was then concentrated under reduced pressure. The oily residue was triturated with MTBE (50 mL) and the precipitate was collected by filtration to afford the desired product (13.65 g, 181% crude yield, xTFA) as light brown solid. LCMS (ESI) *m/z*: [M + H] calcd for $C_{16}H_{18}N_8O$: 339.17; found 339.1. $^1$H NMR (400 MHz, DMSO-$d_6$) δ 8.54 (s, 1H), 8.11 (br s, 1H), 7.75 (br s, 3H), 7.54 (d, *J* = 8.2 Hz, 1H), 7.47 (s, 1H), 7.29 (br d, *J* = 8.4 Hz, 1H), 4.42 (m, *J* = 6.4 Hz, 2H), 2.89-2.71 (m, 2H), 1.98-1.85 (m, 2H), 1.53 (m, *J* = 7.6 Hz, 2H).

**$N$-(4-(4-amino-3-(2-aminobenzo[$d$]oxazol-5-yl)-1$H$-pyrazolo[3,4-$d$]pyrimidin-1-yl)butyl)-1-azido-3,6,9,12,15,18,21,24-octaoxaheptacosan-27-amide (SI-7)**

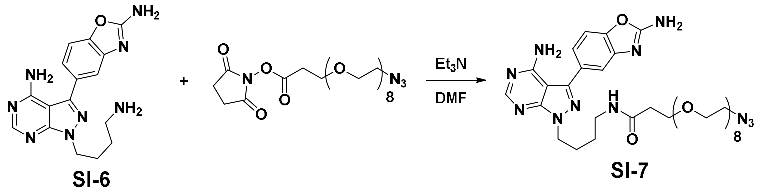


To a solution of 2,5-dioxopyrrolidin-1-yl 1-azido-3,6,9,12,15,18,21,24-octaoxaheptacosan-27-oate (1.86 g, 3.29 mmol, 1.2 equiv) in DMF (66.2 mL) was added **SI-6** (1.20 g, 2.65 mmol, 1.0 equiv) and $NEt_3$ (1.29 mL, 9.26 mmol, 3.5 equiv). The reaction mixture was stirred at room temperature overnight and was then concentrated under reduced pressure. Purification by silica gel chromatography (0→23% MeOH/DCM) afforded the desired product (2.0 g, 96% yield) as light brown residue. LCMS (ESI) *m/z*: [M + H] calcd for $C_{35}H_{53}N_{11}O_{10}$: 788.41; found 788.3. $^1$H NMR (500 MHz, $CDCl_3$) δ 8.35 (s, 1H), 7.61 (s, 1H), 7.42 – 7.35 (m, 2H), 6.74 (br s, 1H), 5.81 (br s, 2H), 4.47 (t, $J$ = 6.9 Hz, 2H), 3.72 – 3.55 (m, 32H), 3.40 (t, $J$ = 5.1 Hz, 2H), 3.30 (q, $J$ = 6.6 Hz, 2H), 2.44 (t, $J$ = 5.8 Hz, 2H), 2.01 (q, $J$ = 7.2 Hz, 2H), 1.58 (q, $J$ = 7.3 Hz, 2H).

**$N$-(4-(4-amino-3-(2-aminobenzo[$d$]oxazol-5-yl)-1$H$-pyrazolo[3,4-$d$]pyrimidin-1-yl)butyl)-1-azido-3,6,9,12,15,18-hexaoxahenicosan-21-amide (SI-8)**

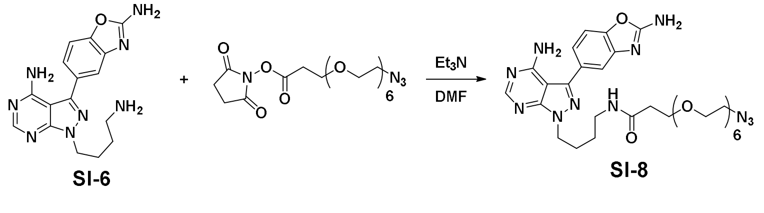

To a solution of 2,5-dioxopyrrolidin-1-yl 1-azido-3,6,9,12,15,18-hexaoxahenicosan-21-oate (263 mg, 552 µmol, 1.3 equiv) in DMA (2.4 mL) was added $NEt_3$ (0.21 mL, 1.51 mmol, 3.4 equiv) followed by a solution of **SI-6** (200 mg, 442 µmol, 1.0 equiv) in DMA (2 mL). The reaction mixture was stirred at room temperature for 3 h. The reaction mixture was then purified by reverse phase chromatography (10→100% MeCN/$H_2O$) to afford the desired product (209 mg, 68% yield) as a colorless oil. LCMS (ESI) *m/z*: [M + H] calcd for $C_{31}H_{45}N_{11}O_8$: 700.35; found 700.3.

**N-(4-(4-amino-3-(2-aminobenzo[*d*]oxazol-5-yl)-1*H*-pyrazolo[3,4-*d*]pyrimidin-1-yl)butyl)-1-azido-3,6,9,12,15-pentaoxaoctadecan-18-amide (SI-9)**

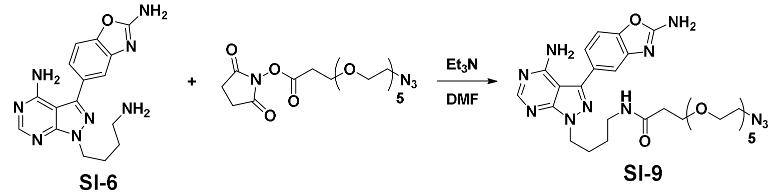


SI-6 SI-9

To a solution of **SI-6** (4.16 g, 9.20 mmol, 1.0 equiv) in DMA (240 mL) was added $NEt_3$ (4.46 mL, 32.1 mmol, 3.5 equiv) followed by a solution of 2,5-dioxopyrrolidin-1-yl 1-azido-3,6,9,12,15-pentaoxaoctadecan-18-oate (5 g, 11.5 mmol, 1.3 equiv) in DMA (15 mL). The reaction mixture was stirred at room temperature for 4.5 h and was then concentrated under reduced pressure. Purification by silica gel chromatography (0→30% MeOH/DCM) to afford the desired product (4.3 g, 71% yield) as a colorless oil. LCMS (ESI) *m/z*: [M + H] calcd for $C_{29}H_{41}N_{11}O_7$: 656.33; found 656.3.

**2-(4-amino-1-(4-aminobutyl)-1*H*-pyrazolo[3,4-*d*]pyrimidin-3-yl)-1*H*-indol-5-ol (SI-12)**

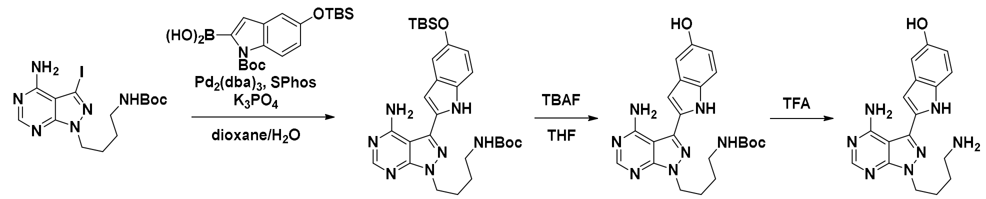


***Step 1*: *tert*-butyl (4-(4-amino-3-(5-((*tert*-butyldimethylsilyl)oxy)-1*H*-indol-2-yl)-1*H*-pyrazolo[3,4-*d*]pyrimidin-1-yl)butyl)carbamate (SI-10)**

To a solution of **SI-4** (1.0 g, 2.3 mmol, 1.0 equiv) in dioxane (10.5 mL) and $H_2O$ (3.5 mL) was added (1-(*tert*-butoxycarbonyl)-5-((*tert*-butyldimethylsilyl)oxy)-1*H*-indol-2-yl)boronic acid (1.54 g, 3.94 mmol, 1.7 equiv), $K_3PO_4$ (1.47 g, 6.93 mmol, 3.0 equiv), $Pd_2(dba)_3$ (211.84 mg, 231.3 μmol, 0.1 equiv), and SPhos (189.95 mg, 462.7 μmol, 0.2 equiv). The sealed tube was heated to 150 °C for 20 min in a microwave. This was repeated for 9 additional batches. The 10 batches were combined, and the reaction mixtures were partitioned between EtOAc (60 mL) and $H_2O$ (80 mL). The aqueous layer was extracted with EtOAc and the combined organic layers were washed with brine, dried, filtered, and concentrated under reduced pressure. Purification by silica gel chromatography (1→75% EtOAc/pet. ether) afforded the desired product (10 g, 78% yield) as a light yellow solid. $^1$H NMR (400 MHz, DMSO-$d_6$) δ 11.30 (s, 1H), 8.08 (s, 1H), 7.14 (d, *J* = 8.4, 3H), 6.86 (d, *J* = 2 Hz, 1H), 6.64 (m, 1H), 6.57 - 6.53 (m, 2H), 4.17 (br t, *J* = 6.8 Hz, 2H), 2.78 - 2.73 (m, 2H), 1.70 - 1.63 (m, 2H), 1.16 (s, 11H), 0.79 (s, 9H), 0.0 (s, 6H).

***Step 2*: *tert*-butyl (4-(4-amino-3-(5-hydroxy-1*H*-indol-2-yl)-1*H*-pyrazolo[3,4-*d*]pyrimidin-1-yl)butyl)carbamate (SI-11)**

To a mixture of **SI-10** (10 g, 18 mmol, 1.0 equiv) in THF (100 mL) was added TBAF•$3H_2O$ (17.2 g, 54.4 mmol, 3.0 equiv) in one portion. The mixture was stirred for 1 h and then $H_2O$ (100 mL) was added to the reaction mixture. The aqueous layer was extracted with

EtOAc and the combined organic layers were washed with brine, dried, filtered, and concentrated under reduced pressure. Purification by silica gel chromatography (1→67% EtOAc/pet. ether) afforded the desired product (7.0 g, 88% yield) as a light pink solid. $^{1}$H NMR (400MHz, DMSO-$d_6$) δ 11.33 (s, 1H), 8.77 (s, 1H), 8.26 (s, 1H), 7.25 (d, $J$ = 8.8 Hz, 1H), 6.93 (d, $J$ = 2.0 Hz, 1H), 6.82 (br t, $J$ = 5.6 Hz, 1H), 6.73 - 6.63 (m, 2H), 4.35 (br t, $J$ = 6.6 Hz, 2H), 2.94 (q, $J$ = 6.6 Hz, 2H), 1.90 - 1.77 (m, 2H), 1.42 - 1.26 (m, 11H).

***Step 3*: 2-(4-amino-1-(4-aminobutyl)-1*H*-pyrazolo[3,4-*d*]pyrimidin-3-yl)-1*H*-indol-5-ol (SI-12)**

To TFA (50.0 mL, 653 mmol, 37.5 equiv) was added **SI-11** (7.60 g, 17.4 mmol, 1.0 equiv) at room temperature. The mixture was stirred for 40 min and was then concentrated under reduced pressure. The oily residue was triturated with MeCN (20 mL), then added dropwise into MTBE (300 mL). The supernatant was removed and then the precipitate was collected by filtration to afford the desired product (7.79 g, 99% yield, TFA) as light yellow solid. LCMS (ESI) *m/z*: [M + H] calcd for $C_{17}H_{19}N_7O$: 338.17; found 338.2. $^{1}$H NMR (400 MHz, DMSO-$d_6$) δ 11.38 (s, 1H), 8.36 (s, 1H), 7.68 (br s, 3H), 7.26 (d, $J$ = 8.6 Hz, 1H), 6.93 (d, $J$ = 2.0 Hz, 1H), 6.75 - 6.66 (m, 2H), 4.41 (br t, $J$ = 6.4 Hz, 2H), 2.87 - 2.74 (m, 2H), 1.99 - 1.87 (m, 2H), 1.51 (q, $J$ = 7.8 Hz, 2H).

***N*-(4-(4-amino-3-(5-hydroxy-1*H*-indol-2-yl)-1*H*-pyrazolo[3,4-*d*]pyrimidin-1-yl)butyl)-1-azido-3,6,9,12,15,18,21,24-octaoxaheptacosan-27-amide (SI-13)**

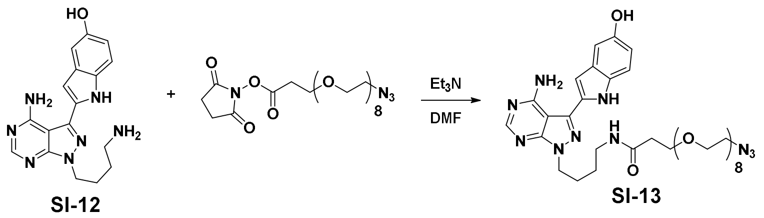

To a solution of 2,5-dioxopyrrolidin-1-yl 1-azido-3,6,9,12,15,18,21,24-octaoxaheptacosan-27-oate (993 mg, 1.76 mmol, 1.5 equiv) in DMA (14.7 mL) was added **SI-12** (0.55 g, 1.2 mmol, 1.0 equiv) and $NEt_3$ (716 µL, 5.14 mmol, 4.3 equiv). The reaction mixture was stirred at room temperature for 4 h and was then concentrated under reduced pressure. Purification by silica gel chromatography (0→20% MeOH/DCM) afforded the desired product (622 mg, 65% yield) as a colorless oil. LCMS (ESI) *m/z*: [M + H] calcd for $C_{36}H_{54}N_{10}O_{10}$: 787.41; found 787.8.

**_N_-(4-(4-amino-3-(5-hydroxy-1*H*-indol-2-yl)-1*H*-pyrazolo[3,4-*d*]pyrimidin-1-yl)butyl)-1-azido-3,6,9,12,15,18-hexaoxahenicosan-21-amide (SI-14)**

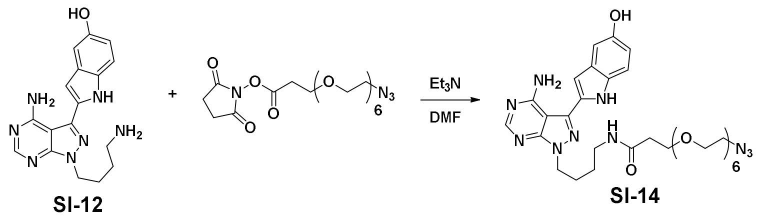

To a solution of **SI-12** (251 mg, 674 µmol, 1.1 equiv) in DMA (2.2 mL) was added $NEt_3$ (311 µL, 2.24 mmol, 3.5 equiv) followed by a solution of 2,5-dioxopyrrolidin-1-yl 1-azido-3,6,9,12,15,18-hexaoxahenicosan-21-oate (306.2 mg, 642 µmol, 1.0 equiv) in DMA (1.0 mL). The reaction mixture was stirred at room temperature for 16 h and was then concentrated under reduced pressure. Purification by silica gel chromatography (0→30% MeOH/DCM) to afford the

desired product (342 mg, 76% yield) as a colorless oil. LCMS (ESI) *m/z*: [M + H] calcd for $C_{32}H_{46}N_{10}O_8$: 699.36; found 699.2.

**1-(4-(4-(1-azido-3,6,9,12,15,18,21,24-octaoxaheptacosan-27-oyl)piperazin-1-yl)-3-(trifluoromethyl)phenyl)-8-(6-methoxypyridin-3-yl)-3-methyl-1,3-dihydro-2*H*-imidazo[4,5-*c*]quinolin-2-one (SI-15)**

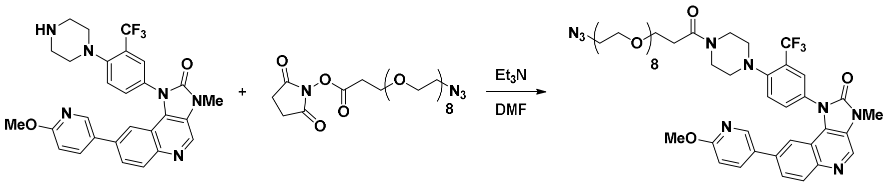


To a solution of BGT226 **19** (50 mg, 93.5 μmol 1.0 equiv) in DMF (2.7 mL) was added 2,5-dioxopyrrolidin-1-yl 1-azido-3,6,9,12,15,18,21,24-octaoxaheptacosan-27-oate (65.4 mg, 116 μmol, 1.3 equiv) followed by slow addition of $NEt_3$ (45.4 μL, 327 μmol, 3.5 equiv). The reaction was stirred for 3 h and then concentrated under reduced pressure. Purification by silica gel chromatography (0→5% MeOH/DCM) afforded the desired product (58.3 mg, 63% yield) as a colorless oil. LCMS (ESI) *m/z*: [M + H] calcd for $C_{47}H_{61}F_3N_9O_{11}$: 984.44; found 984.5. $^1$H NMR (500 MHz, $CDCl_3$) δ 8.74 (s, 1H), 8.13 (d, *J* = 8.9 Hz, 1H), 8.05 (d, *J* = 2.6 Hz, 1H), 7.83 (d, *J* = 2.5 Hz, 1H), 7.71 (m, 2H), 7.56 (d, *J* = 8.5 Hz, 1H), 7.48 (dd, *J* = 8.6, 2.6 Hz, 1H), 7.05 (s, 1H), 6.66 (d, *J* = 8.6 Hz, 1H), 3.90 (m, 4H), 3.80 (t, *J* = 6.7 Hz, 2H), 3.69 – 3.49 (m, 36H), 3.34 (t, *J* = 5.1 Hz, 2H), 3.02 (m, 4H), 2.67 (m, 2H).

**1-(4-(4-(1-azido-3,6,9,12,15,18-hexaoxahenicosan-21-oyl)piperazin-1-yl)-3-(trifluoromethyl)phenyl)-8-(6-methoxypyridin-3-yl)-3-methyl-1,3-dihydro-2*H*-imidazo[4,5-*c*]quinolin-2-one (SI-16)**

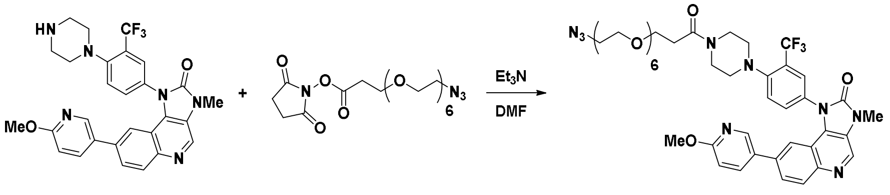


To a solution of BGT226 **19** (225 mg, 420 µmol 1.0 equiv) in DMA (2.0 mL) was added a solution of 2,5-dioxopyrrolidin-1-yl 1-azido-3,6,9,12,15,18-hexaoxahenicosan-21-oate (250 mg, 524 µmol, 1.3 equiv) in DMA (2.2 mL) followed by slow addition of $NEt_3$ (203 µL, 1.47 mmol, 3.5 equiv). The reaction was stirred for 6 h and then concentrated under reduced pressure. Purification by reverse phase chromatography (10→100% MeCN/$H_2O$) afforded the desired product (260.3 mg, 69% yield) as a colorless oil. LCMS (ESI) *m/z*: [M + H] calcd for $C_{43}H_{52}F_3N_9O_9$: 896.39; found 896.5. $_1$H NMR (500 MHz, $CDCl_3$) δ 8.79 (s, 1H), 8.18 (d, *J* = 8.9, Hz, 1H), 8.09 (dd, *J* = 2.6, 0.8Hz, 1H), 7.85 (d, *J* = 2.5 Hz, 1H), 7.74 (ddd, *J* = 8.4, 3.5, 2.2 Hz, 2H), 7.59 (d, *J* = 8.4 Hz, 1H), 7.51 (dd, *J* = 8.6, 2.6 Hz, 1H), 7.09 (dd, *J* = 2.1, 0.6 Hz, 1H), 6.70 (dd, *J* = 8.6, 0.7 Hz, 1H), 3.93 (m, 4H), 3.83 (t, *J* = 6.7Hz, 2H), 3.69 – 3.58 (m, 28H), 3.37 (t, *J* = 5.1 Hz, 2H), 3.08 – 2.97 (m, 4H), 2.70 (td, *J* = 6.7, 2.7 Hz, 2H).

**1-(4-aminobutyl)-3-(1*H*-pyrrolo[2,3-*b*]pyridin-5-yl)pyrazolo[3,4-*d*]pyrimidin-4-amine (SI-18)**

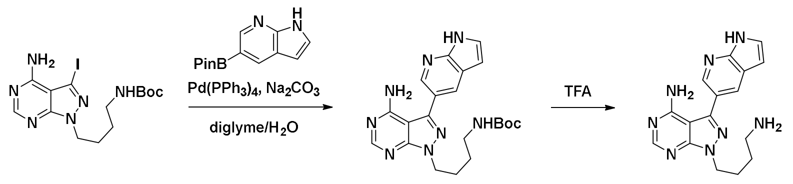


SI-4 SI-17 SI-18

***Step 1*****: Synthesis of *****tert*****-butyl *****N*****-[4-[4-amino-3-(1*****H*****-indol-5-yl)pyrazolo[3,4-*****d*****]pyrimidin-1-yl]butyl]carbamate (SI-17)**

To a bi-phasic suspension of **SI-4** (8 g, 18.51 mmol, 1 equiv), 5-(4,4,5,5-tetramethyl-1,3,2-dioxaborolan-2-yl)-1*H*-pyrrolo[2,3-*b*]pyridine (5.42 g, 22.21 mmol, 1.2 equiv), and $Na_2CO_3$ (9.81 g, 92.54 mmol, 5 equiv) in diglyme (160 mL) and $H_2O$ (80 mL) was added $Pd(PPh_3)_4$ (2.14 g, 1.85 mmol, 0.1 equiv). The mixture was stirred at 110 °C for 3 h. The reaction mixture was cooled to room temperature, filtered, and the filtrate was partitioned between EtOAc (500 mL) and $H_2O$ (500 mL). The aqueous layer was separated and extracted with EtOAc (3 x 300 mL). The combined organic layers were washed with brine (20 mL), dried over $Na_2SO_4$, filtered, and concentrated under reduced pressure. The residue was purified by silica gel chromatography (0→100% EtOAc/pet. ether then 20% MeOH/EtOAc) to afford the desired product (6.6 g, 85% yield) as a yellow solid. LCMS (ESI) *m/z*: [M + H] calcd for $C_{22}H_{27}N_7O_2$: 422.22; found 423.3. $^1$H NMR (400 MHz, MeOD) δ ppm 8.52 (d, $J$ = 2 Hz, 1H), 8.30 (d, $J$ = 2 Hz, 1H), 8.27 (s, 1H), 7.52 (d, $J$ = 3.6 Hz, 1H), 6.63 (d, $J$ = 3.6 Hz, 1H), 4.55 (t, $J$ = 7.0 Hz, 2H), 3.01 (m, 2H), 1.99 – 1.94 (m, 2H), 1.54 – 1.47 (m, 2H), 1.40 (s, 9H).

***Step 2*****: Synthesis of 1-(4-aminobutyl)-3-(1*****H*****-pyrrolo[2,3-*****b*****]pyridin-5-yl)pyrazolo[3,4-*****d*****]pyrimidin-4-amine (SI-18)**

To **SI-17** (6.6 g, 15.66 mmol, 1 equiv) was added TFA (66 mL), which was then stirred at room temperature for 30 min. The reaction solution was concentrated under reduced pressure and

then MTBE (400 mL) was added to the residue. The suspension was stirred for 15 min, then the solid was filtered, and the solid cake dried under reduced pressure to afford the desired product (10.2 g, 97% yield) as a yellow solid. LCMS (ESI) *m/z*: [M + H] calcd for $C_{16}H_{18}N_8$: 323.17; found 323.1. $^1$H NMR (400 MHz, MeOD) δ ppm 8.54 (d, *J* = 1.98 Hz, 1H), 8.43 (s, 1H), 8.37 (d, *J* = 1.98 Hz, 1H), 7.57 (d, *J* = 3.53 Hz, 1H), 6.68 (d, *J* = 3.31 Hz, 1H), 4.58 (t, *J* = 6.73 Hz, 2H), 3.00 (t, *J* = 7.72 Hz, 2H), 2.16 - 2.04 (m, 2H), 1.72 (q, *J*=7.77 Hz, 2H).

**_N_-(4-(4-amino-3-(1_H_-pyrrolo[2,3-_b_]pyridin-5-yl)-1_H_-pyrazolo[3,4-_d_]pyrimidin-1-yl)butyl)-1-azido-3,6,9,12,15,18,21,24-octaoxaheptacosan-27-amide (SI-19)**

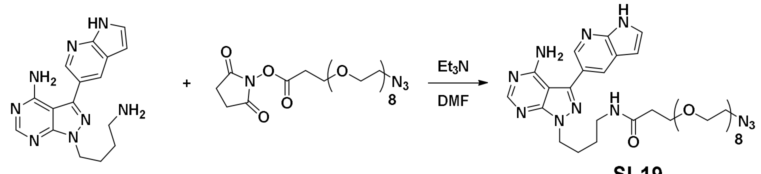


**SI-18** **SI-19**

To a solution of 2,5-dioxopyrrolidin-1-yl 1-azido-3,6,9,12,15,18,21,24-octaoxaheptacosan-27-oate (193.4 mg, 341 µmol, 1.3 equiv) in DMA (1 mL) was added $NEt_3$ (132 µL, 954 µmol, 3.5 equiv) followed by a solution of **SI-18** (118 mg, 272 µmol, 1.0 equiv) in DMA (1.7 mL). The reaction mixture was stirred at room temperature for 5 h. The reaction mixture was then purified by reverse phase chromatography (40→100% MeCN/$H_2O$) to afford the desired product (115.5 mg, 55% yield) as a colorless oil. LCMS (ESI) *m/z*: [M + H] calcd for $C_{35}H_{53}N_{11}O_9$: 772.41; found 772.3. $^1$H NMR (500 MHz, $CDCl_3$) δ 10.32 (s, 1H), 8.58 (s, 1H), 8.36 (s, 1H), 8.22 (d, *J* = 1.9 Hz, 1H), 7.46 (d, *J* = 3.6 Hz, 1H), 6.76 (m, 1H), 6.59 (d, *J* = 3.5 Hz, 1H), 4.48 (t, *J* = 7.0 Hz, 2H), 3.73 – 3.49 (m, 32H), 3.37 (t, *J* = 5.1 Hz, 2H), 3.30 (q, *J* = 6.6 Hz, 2H), 2.43 (t, *J* = 5.8 Hz, 2H), 2.02 (p, *J* = 7.1 Hz, 2H), 1.57 (p, *J* = 7.2 Hz, 2H).

**$N$-(4-(4-amino-3-(1$H$-pyrrolo[2,3-$b$]pyridin-5-yl)-1$H$-pyrazolo[3,4-$d$]pyrimidin-1-yl)butyl)-1-azido-3,6,9,12,15,18-hexaoxahenicosan-21-amide (SI-20)**

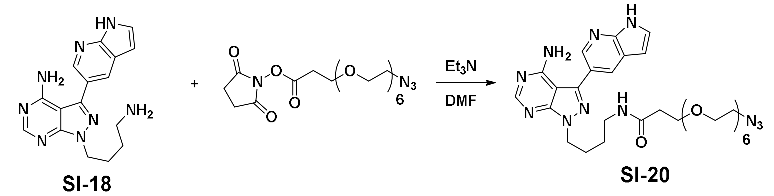


To a solution of 2,5-dioxopyrrolidin-1-yl 1-azido-3,6,9,12,15,18-hexaoxahenicosan-21-oate (250 mg, 524 μmol, 1.3 equiv) in DMA (2 mL) was added $NEt_3$ (202 μL, 1.46 mmol, 3.5 equiv) followed by a solution of **SI-18** (182 mg, 419 μmol, 1.0 equiv) in DMA (2.2 mL). The reaction mixture was stirred at room temperature for 5 h. The reaction mixture was then purified by reverse phase chromatography (40→100% MeCN/$H_2O$) to afford the desired product (149.5 mg, 52% yield) as a colorless oil. LCMS (ESI) *m/z*: [M + H] calcd for $C_{31}H_{45}N_{11}O_7$: 684.36; found 684.3. $^1$H NMR (500 MHz, $CDCl_3$) δ 10.39 (s, 1H), 8.57 (s, 1H), 8.34 (s, 1H), 8.22 (d, $J$ = 2.0 Hz, 1H), 7.46 (d, $J$ = 3.5 Hz, 1H), 6.77 (m, 1H), 6.58 (d, $J$ = 3.5 Hz, 1H), 4.48 (t, $J$ = 7.0 Hz, 2H), 3.73 – 3.48 (m, 27H), 3.36 (t, $J$ = 5.1 Hz, 2H), 3.29 (t, $J$ = 6.5 Hz, 2H), 2.44 (t, $J$ = 5.8 Hz, 2H), 2.01 (p, $J$ = 7.1 Hz, 2H), 1.57 (p, $J$ = 7.2 Hz, 2H).

**5-(4-amino-1-((1,2,3,4-tetrahydroisoquinolin-6-yl)methyl)-1$H$-pyrazolo[3,4-$d$]pyrimidin-3-yl)benzo[$d$]oxazol-2-amine trifluoroacetic acid salt (SI-23)**

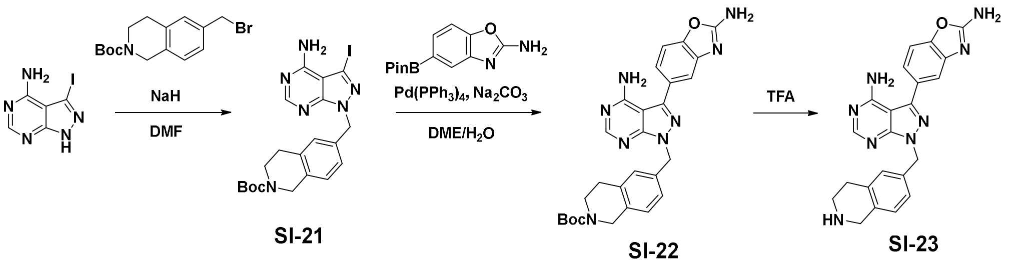


***Step 1*: Synthesis of *tert*-butyl 6-((4-amino-3-iodo-1*H*-pyrazolo[3,4-*d*]pyrimidin-1-yl)methyl)-3,4-dihydroisoquinoline-2(1*H*)-carboxylate (SI-21)**

To a suspension of 3-iodo-1*H*-pyrazolo[3,4-*d*]pyrimidin-4-amine (5 g, 19.16 mmol, 1.0 equiv) in DMF (50 mL) at 0 °C was added NaH (766.22 mg, 19.16 mmol, 60 wt.%, 1.0 equiv). The mixture was stirred at 0 °C for 30 min. To the reaction mixture was added a solution of *tert*-butyl 6-(bromomethyl)-3,4-dihydroisoquinoline-2(1*H*)-carboxylate (6.87 g, 21.07 mmol, 1.1 equiv) in DMF (30 mL) at 0 °C. The mixture was stirred at room temperature for 2 h. The mixture was then cooled to 0 °C and $H_2O$ (400 mL) was added. The mixture was stirred for 30 min and the resulting precipitate was collected by filtration to afford the desired product (9.7 g, 76% yield) as a light yellow solid, which was used directly in the next step. $^1$H NMR (400 MHz, DMSO-$d_6$) δ 8.24 (s, 1H), 7.12 – 7.10 (m, 1H), 7.05 – 7.03 (m, 2H), 5.41 (s, 2H), 4.44 (s, 2H), 3.50 (t, $J$ = 6 Hz, 2H), 2.71 (t, $J$ = 6 Hz, 2H), 1.41 (s, 9H).

***Step 2*: Synthesis of *tert*-butyl 6-((4-amino-3-(2-aminobenzo[*d*]oxazol-5-yl)-1*H*-pyrazolo[3,4-*d*]pyrimidin-1-yl)methyl)-3,4-dihydroisoquinoline-2(1*H*)-carboxylate (SI-22)**

To a bi-phasic suspension of **SI-21** (9.7 g, 14.63 mmol, 1.0 equiv), 5-(4,4,5,5-tetramethyl-1,3,2-dioxaborolan-2-yl)benzo[*d*]oxazol-2-amine (4.57 g, 17.55 mmol, 1.2 equiv), and $Na_2CO_3$ (7.75 g, 73.14 mmol, 5.0 equiv) in DME (120 mL) and $H_2O$ (60 mL) at room temperature was added $Pd(PPh_3)_4$ (1.69 g, 1.46 mmol, 0.1 equiv). The mixture was stirred at 110

°C for 3 h. The reaction mixture was then cooled to room temperature and partitioned between EtOAc (100 mL) and $H_2O$ (100 mL). The aqueous layer was separated and extracted with EtOAc (2 x 60 mL) and the organic layers were combined, washed with brine (80 mL), dried over $Na_2SO_4$, filtered, and concentrated under reduced pressure. The residue was purified by silica gel chromatography (1→100% EtOAc/pet. ether then 20→50% MeOH/EtOAc) to afford the desired product (4.5 g, 58% yield) as a light yellow solid. $^1$H NMR (400 MHz, DMSO-$d_6$) δ 8.27 (s, 1H), 7.53 (s, 2H), 7.45 (d, *J* = 8.0 Hz, 1H), 7.39 (d, *J* = 1.6 Hz, 1H), 7.22 (dd, *J* = 1.8, 8.0 Hz, 1H), 7.11 (s, 3H), 5.49 (s, 2H), 4.43 (br s, 2H), 3.50 (t, *J* = 5.8 Hz, 2H), 2.72 (t, *J* = 5.8 Hz, 2H), 1.40 (s, 9H).

***Step 3*: Synthesis of 5-(4-amino-1-((1,2,3,4-tetrahydroisoquinolin-6-yl)methyl)-1*H*-pyrazolo[3,4-*d*]pyramidin-3-yl)benzo[*d*]oxazol-2-amine (SI-23)**

To TFA (32.5 mL, 438.97 mmol, 50.0 equiv) was added **SI-22** (4.5 g, 8.78 mmol, 1.0 equiv) at room temperature. The mixture was stirred for 30 min and then concentrated under reduced pressure. The oily residue was triturated with MeCN (8 mL) and then added to MTBE (350 mL) over 10 min. The supernatant was removed and then the precipitate was collected by filtration under $N_2$ to afford the desired product (5.72 g, over 100% yield, TFA) as a light pink solid. LCMS (ESI) *m/z*: [M + H] calcd for $C_{22}H_{20}N_8O$: 413.18; found 413.2. $^1$H NMR (400 MHz, MeOD) δ 8.45 (s, 1H), 7.62 - 7.55 (m, 2H), 7.51 - 7.46 (m, 1H), 7.33 - 7.28 (m, 2H), 7.20 (d, *J* = 8.4 Hz, 1H), 5.65 (s, 2H), 4.33 (s, 2H), 3.47 (t, *J* = 6.2 Hz, 2H), 3.09 (br t, *J* = 6.2 Hz, 2H).

**27-(6-((4-amino-3-(2-aminobenzo[*d*]oxazol-5-yl)-1*H*-pyrazolo[3,4-*d*]pyrimidin-1-yl)methyl)-3,4-dihydroisoquinolin-2(1*H*)-yl)-1-azido-3,6,9,12,15,18,21,24-octaoxaheptacosan-27-one (SI-24)**

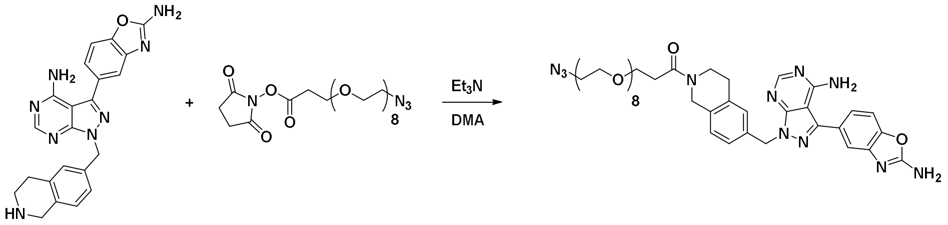


SI-23 SI-24

To a solution of 2,5-dioxopyrrolidin-1-yl 1-azido-3,6,9,12,15,18,21,24-octaoxaheptacosan-27-oate (976 mg, 1.73 mmol, 1.2 equiv) in DMA (5 mL) was added $NEt_3$ (706 µL, 5.07 mmol, 3.5 equiv) followed by a solution of **SI-23** (600 mg, 1.45 mmol, 1.0 equiv) in DMA (2.2 mL). The reaction mixture was stirred at room temperature for 4 h. The reaction mixture was then purified by silica gel chromatography (0→20% MeOH/DCM) to afford the desired product (570 mg, 46% yield) as a colorless oil. LCMS (ESI) *m/z*: [M + H] calcd for $C_{41}H_{55}N_{11}O_{10}$: 862.42; found 862.3.

***N*-(2-((4-(7-(6-aminopyridin-3-yl)-2,3,4,5-tetrahydrobenzo[*f*][1,4]oxazepine-4-carbonyl)-2-fluoro-3-methylphenyl)sulfonyl)ethyl)-1-(4-(4-(((1*R*,2*R*,4*S*)-4-((*R*)-2-((3*S*,6*R*,7*E*,9*R*,10*R*,12*R*,14*S*,15*E*,17*E*,19*E*,21*S*,23*S*,26*R*,27*R*,34a*S*)-9,27-dihydroxy-10,21-dimethoxy-6,8,12,14,20,26-hexamethyl-1,5,11,28,29-pentaoxo-1,4,5,6,9,10,11,12,13,14,21,22,23,24,25,26,27,28,29,31,32,33,34,34a-tetracosahydro-3*H*-**

**5-(4-amino-1-(piperidin-4-ylmethyl)-1*H*-pyrazolo[3,4-*d*]pyrimidin-3-yl)benzo[*d*]oxazol-2-amine trifluoroacetic acid salt (SI-27)**

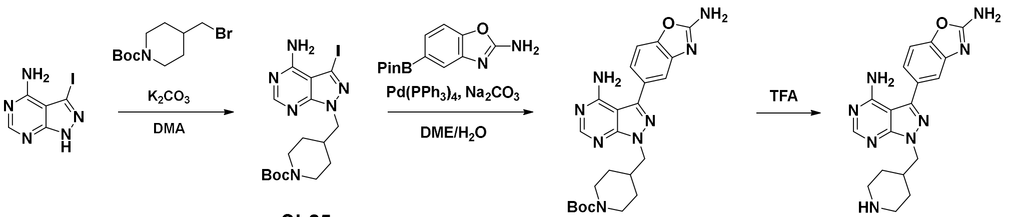


***Step 1*: Synthesis of *tert*-butyl 4-((4-amino-3-iodo-1*H*-pyrazolo[3,4-*d*]pyrimidin-1-yl)methyl)piperidine-1-carboxylate (SI-25)**

To a solution of 3-iodo-1*H*-pyrazolo[3,4-*d*]pyrimidin-4-amine (3 g, 11.49 mmol, 1.0 equiv) in DMA (30 mL) was added *tert*-butyl 4-(bromomethyl)piperidine-1-carboxylate (3.36 g, 12.07 mmol, 1.05 equiv) and $K_2CO_3$ (4.77 g, 34.48 mmol, 3.0 equiv), then the reaction was stirred at 80 °C for 3 h. The reaction mixture was filtered and the filtrate was poured into $H_2O$ (200 mL). A solid precipitated, which was then filtered to afford the desired product (3 g, 57% yield) as a light yellow solid. LCMS (ESI) *m/z*: [M + H] calcd for $C_{16}H_{23}IN_6O_2$: 459.10; found 459.1.

***Step 2*: Synthesis of *tert*-butyl 4-((4-amino-3-(2-aminobenzo[*d*]oxazol-5-yl)-1*H*-pyrazolo[3,4-*d*]pyrimidin-1-yl)methyl)piperidine-1-carboxylate (SI-26)**

Two batches were run in parallel. To a bi-phasic suspension of **SI-25** (3 g, 6.55 mmol, 1.0 equiv), 5-(4,4,5,5-tetramethyl-1,3,2-dioxaborolan-2-yl)benzo[*d*]oxazol-2-amine (2.04 g, 7.86 mmol, 1.2 equiv), and $Na_2CO_3$ (3.47 g, 32.73 mmol, 5.0 equiv) in DME (60 mL) and $H_2O$ (30 mL) at room temperature was added $Pd(PPh_3)_4$ (756.43 mg, 654.60 µmol, 0.1 equiv). The mixture was stirred at 110 °C for 3 h and the two batches were combined. The reaction mixture was cooled and partitioned between EtOAc (500 mL) and $H_2O$ (500 mL). The aqueous layer was separated and extracted with EtOAc (3 x 300 mL). The organic layers were combined, washed

with brine (20 mL), dried over $Na_2SO_4$, filtered, and the filtrate was concentrated under reduced pressure to afford the desired product (4.5 g, 74% yield) as a yellow solid. LCMS (ESI) *m/z*: [M + H] calcd for $C_{23}H_{28}N_8O_3$: 465.24; found 465.2.

***Step 3*: Synthesis of 5-(4-amino-1-(piperidin-4-ylmethyl)-1*H*-pyrazolo[3,4-*d*]pyrimidin-3-yl)benzo[*d*]oxazol-2-amine (SI-27)**

A solution of **SI-26** (2.5 g, 5.38 mmol, 1.0 equiv) in TFA (25 mL) was stirred at room temperature for 30 min. The reaction solution was then concentrated under reduced pressure and the residue was added to MTBE (400 mL). A solid precipitated, which was then filtered to afford the desired product (2.7 g, over 100 % yield, TFA) as a yellow solid. LCMS (ESI) *m/z*: [M + H] calcd for $C_{18}H_{20}N_8O$: 365.18; found 365.1. $^1H$ NMR (400 MHz, MeOD) δ 8.38 (s, 1H), 7.58 - 7. 45 (m, 2H), 7.40 (d, *J* = 8.16 Hz, 1H), 4.86 (s, 2H), 4.45 (d, *J* = 6.62 Hz, 2H), 3.41 (br d, *J* = 12.35 Hz, 2H), 3.10 - 2.88 (m, 2H), 2.42 (br s, 1H), 1.92 (br d, *J* = 13.89 Hz, 2H), 1.71 - 1.46 (m, 2H).

**27-(4-((4-amino-3-(2-aminobenzo[*d*]oxazol-5-yl)-1*H*-pyrazolo[3,4-*d*]pyrimidin-1-yl)methyl)piperidin-1-yl)-1-azido-3,6,9,12,15,18,21,24-octaoxaheptacosan-27-one (SI-28)**

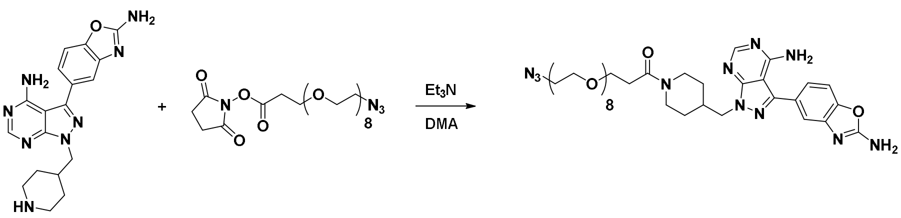


SI-27 SI-28

To a solution of 2,5-dioxopyrrolidin-1-yl 1-azido-3,6,9,12,15,18,21,24-octaoxaheptacosan-27-oate (294 mg, 522 μmol, 1.3 equiv) in DMA (2 mL) was added $NEt_3$ (202

μL, 1.46 mmol, 3.5 equiv) followed by a solution of **SI-27** (200 mg, 418 μmol, 1.0 equiv) in DMA (2.2 mL). The reaction mixture was stirred at room temperature for 3 h. The reaction mixture was then purified by reverse phase chromatography (10→100% MeCN/$H_2O$) to afford the desired product (181.0 mg, 53% yield) as a colorless oil. LCMS (ESI) *m/z*: [M + H] calcd for $C_{37}H_{55}N_{11}O_{10}$: 814.42; found 814.3.

**5-(1-(4-aminobutyl)-4-(dimethylamino)-1*H*-pyrazolo[3,4-*d*]pyrimidin-3-yl)benzo[*d*]oxazol-2-amine (SI-31)**

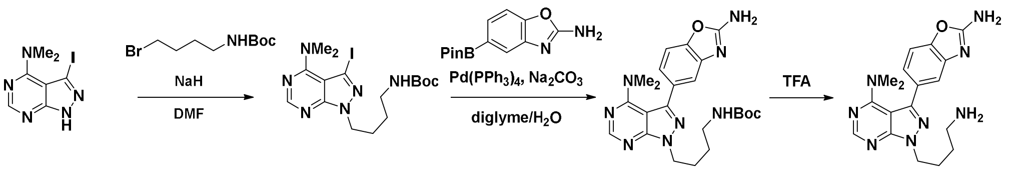


**SI-29** **SI-30** **SI-31**

***Step 1*: Synthesis of *tert*-butyl (4-(4-(dimethylamino)-3-iodo-1*H*-pyrazolo[3,4-*d*]pyrimidin-1-yl)butyl)carbamate (SI-29)**

To a suspension of 3-iodo-*N*,*N*-dimethyl-1*H*-pyrazolo[3,4-*d*]pyrimidin-4-amine (1.7 g, 5.88 mmol, 1.0 equiv) in DMF (20 mL) at 0 °C was added NaH (247 mg, 6.17 mmol, 60 wt.%, 1.05 equiv). The mixture was stirred at 0 °C for 30 min. To the reaction mixture was then added a solution of *tert*-butyl (4-bromobutyl)carbamate (1.81 mL, 8.82 mmol, 1.5 equiv) in DMF (10 mL) at 0 °C. The mixture was stirred at room temperature for 2 h and then $H_2O$ (100 mL) at 0 °C. The mixture was stirred for an additional 30 min at 0 °C and the resulting precipitate was collected by filtration. The residue was purified by silica gel chromatography (0→75% EtOAc/pet. ether) to afford the desired product (2.0 g, 56% yield) as a white solid. $^1$H NMR

(400MHz, DMSO-$d_6$) δ 8.24 (s, 1H), 6.79 (br t, *J* = 5.4 Hz, 1H), 4.29 (t, *J* = 6.8 Hz, 2H), 3.34 (s, 6H), 2.96 - 2.83 (m, 2H), 1.75 (q, *J* = 7.2 Hz, 2H), 1.41 - 1.23 (m, 11H).

***Step 2*: Synthesis of *tert*-butyl (4-(3-(2-aminobenzo[*d*]oxazol-5-yl)-4-(dimethylamino)-1*H*-pyrazolo[3,4-*d*]pyrimidin-1-yl)butyl)carbamate (SI-30)**

To a bi-phasic suspension of **SI-29** (4.0 g, 8.69 mmol, 1.0 equiv), 5-(4,4,5,5-tetramethyl-1,3,2-dioxaborolan-2-yl)benzo[*d*]oxazol-2-amine (3.4 g, 13.03 mmol, 1.5 equiv), and $Na_2CO_3$ (4.6 g, 43.45 mmol, 5.0 equiv) in DME (80 mL) and $H_2O$ (40 mL) was added $Pd(PPh_3)_4$ (1.0 g, 868.98 µmol, 0.1 equiv). The mixture was stirred at 110 °C for 3 h and then the reaction mixture was then cooled and partitioned between EtOAc (300 mL) and $H_2O$ (600 mL). The aqueous layer was separated and extracted with EtOAc (2 x 100 mL) and the combined organic layers were washed with brine (2 x 60 mL), dried over $Na_2SO_4$, filtered, and concentrated under reduced pressure. The crude material was purified by silica gel column chromatography (50% EtOAc/hexanes followed by 20% MeOH/EtOAc) to afford the desired product (3.2 g, 78.9% yield) as a light brown solid. $^1$H NMR (400 MHz, DMSO-$d_6$) δ 8.30 (s, 1H), 7.50 (s, 2H), 7.41 (d, *J* = 8.2 Hz, 1H), 7.35 (d, *J* = 1.6 Hz, 1H), 7.14 (dd, *J* = 1.6, 8.2 Hz, 1H), 6.78 (br t, *J* = 5.0 Hz, 1H), 4.35 (br t, *J* = 6.8 Hz, 2H), 2.93 (m, *J* = 6.4 Hz, 2H), 2.84 (s, 6H), 1.82 (m, *J* = 7.2 Hz, 2H), 1.41-1.26 (m, 11H).

***Step 3*: Synthesis of 5-(1-(4-aminobutyl)-4-(dimethylamino)-1*H*-pyrazolo[3,4-*d*]pyrimidin-3-yl)benzo[*d*]oxazol-2-amine (SI-31)**

To TFA (20.8 mL, 281.3 mmol, 36.5 equiv) was added **SI-30** (3.6 g, 7.72 mmol, 1.0 equiv) at room temperature. The mixture was stirred for 30 min, at which point the mixture was concentrated under reduced pressure. The oily residue was triturated with MeCN (8 mL) and

MTBE (60 mL) for 10 min. The supernatant was removed and then the precipitate was collected by filtration under $N_2$ to afford the crude product (4.0 g, TFA) as a light brown solid.

To a 1 M NaOH (107.2 mL, 14.7 equiv) solution was added the crude product (3.5 g, TFA) at room temperature. The mixture was stirred for 10 min and then the aqueous phase was extracted with DCM (3 x 50 mL). The combined organic layers were washed with brine (50 mL), dried with $Na_2SO_4$, filtered, and concentrated under reduced pressure. TFA (0.539 mL, 7.28 mmol) was added and concentrated under reduced pressure. MeCN (10 mL) was then added, followed by MTBE (150 mL). The resulting precipitate was collected by filtration to afford the desired product (1.3 g, 37% yield, TFA) as a light brown solid. LCMS (ESI) *m/z*: [M + H] calcd for $C_{18}H_{22}N_8O$: 367.19; found 367.1. $^1$H NMR (400 MHz, DMSO-$d_6$) δ 8.33 (s, 1H), 7.64 (br s, 4H), 7.44 (d, $J$ = 8.2 Hz, 1H), 7.38 (s, 1H), 7.16 (br d, $J$ = 8.1 Hz, 1H), 4.39 (br t, $J$ = 6.6 Hz, 2H), 2.86 (s, 6H), 2.82 (m, 2H), 1.97-1.85 (m, 2H), 1.53 (m, 2H).

**_N_-(4-(3-(2-aminobenzo[_d_]oxazol-5-yl)-4-(dimethylamino)-1_H_-pyrazolo[3,4-_d_]pyrimidin-1-yl)butyl)-1-azido-3,6,10,13,16,19,22,25-octaoxaoctacosan-28-amide (SI-32)**

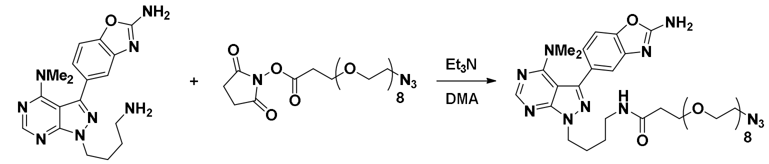


**SI-31** **SI-32**

To a solution of **SI-31** (0.5 g, 1.36 mmol, 1.0 equiv) and 2,5-dioxopyrrolidin-1-yl 1-azido-3,6,9,12,15,18,21,24-octaoxaheptacosan-27-oate (959 mg, 1.70 mmol, 1.25 equiv) in DMA (34 mL) was added $NEt_3$ (662 µL, 3.5 mmol). The reaction mixture was stirred at room temperature for 18 h and was then concentrated under reduced pressure. Purification by silica gel

chromatography (0→20% MeOH/DCM) afforded the desired product (1.0 g, 91% yield) as a colorless oil. LCMS (ESI) *m/z*: [M + H] calcd for $C_{37}H_{57}N_{11}O_{10}$: 816.44; found 816.4.

**1-amino-*N*-(4-(4-amino-3-(2-aminobenzo[*d*]oxazol-5-yl)-1*H*-pyrazolo[3,4-*d*]pyrimidin-1-yl)butyl)-3,6,10,13,16,19,22,25-octaoxaoctacosan-28-amide (SI-34)**

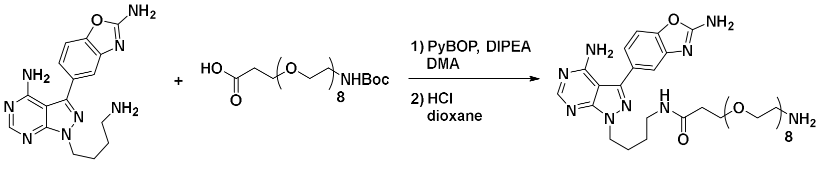


**SI-6** **SI-34**

***Step 1*: Synthesis of *tert*-butyl (33-(4-amino-3-(2-aminobenzo[*d*]oxazol-5-yl)-1*H*-pyrazolo[3,4-*d*]pyrimidin-1-yl)-28-oxo-3,6,10,13,16,19,22,25-octaoxa-29-azatritriacontyl)carbamate (SI-33)**

To a solution of **SI-6** (57.4 mg, 127 µmol, 1.0 equiv), 2,2-dimethyl-4-oxo-3,8,11,15,18,21,24,27,30-nonaoxa-5-azatritriacontan-33-oic acid (125 mg, 230 µmol, 1.8 equiv) in DMA (1.3 mL) was added DIPEA (88.9 µL, 511 µmol, 4.0 equiv) followed by PyBOP (86.3 mg, 166 µmol, 1.3 equiv). The reaction was stirred at room temperature for 2.5 h and then the reaction mixture was concentrated under reduced pressure and the crude residue was purified by reverse phase chromatography (10→100% MeCN/$H_2O$) to afford the desired product (54.3 mg, 50% yield) as a colorless oil. $^1H$ NMR (500 MHz, $CDCl_3$) δ 8.34 (s, 1H), 7.58 (s, 1H), 7.41 – 7.30 (m, 2H), 6.82 (s, 1H), 6.24 (s, 2H), 5.40 (s, 1H), 4.45 (t, *J* = 6.8 Hz, 2H), 3.80 – 3.45 (m, 35H), 3.30 (dq, *J* = 12.9, 5.9 Hz, 4H), 2.43 (t, *J* = 5.8 Hz, 2H), 2.05 – 1.94 (m, 2H), 1.55 (p, *J* = 7.1 Hz, 2H), 1.42 (s, 9H).

***Step 2*: Synthesis of 1-amino-*N*-(4-(4-amino-3-(2-aminobenzo[*d*]oxazol-5-yl)-1*H*-pyrazolo[3,4-*d*]pyrimidin-1-yl)butyl)-3,6,10,13,16,19,22,25-octaoxaoctacosan-28-amide (SI-34)**

To a solution of **SI-33** (54 mg, 62.6 µmol, 1.0 equiv) in DCM (2 mL) at 0 °C was added TFA (400 µL, 62.6 µmol, 1.0 equiv). The reaction solution was stirred at room temperature for 1 h. The reaction solution was then concentrated under reduced pressure to afford the crude desired product (44.4 mg, 81% yield) as a colorless oil, which was used directly in the next step.

**1-amino-27-(6-((4-amino-3-(2-aminobenzo[*d*]oxazol-5-yl)-1*H*-pyrazolo[3,4-*d*]pyrimidin-1-yl)methyl)-3,4-dihydroisoquinolin-2(1*H*)-yl)-3,6,9,12,15,18,21,24-octaoxaheptacosan-27-one (SI-36)**

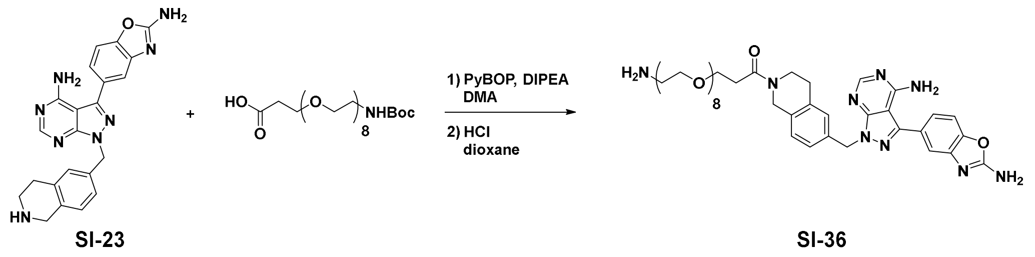


***Step 1*: Synthesis of *tert*-butyl (27-(6-((4-amino-3-(2-aminobenzo[*d*]oxazol-5-yl)-1*H*-pyrazolo[3,4-*d*]pyrimidin-1-yl)methyl)-3,4-dihydroisoquinolin-2(1*H*)-yl)-27-oxo-3,6,9,12,15,18,21,24-octaoxaheptacosyl)carbamate (SI-35)**

To a solution of **SI-23** (1.0 g, 2.42 mmol, 1.0 equiv), 1-{[(*tert*-butoxy)carbonyl]amino}-3,6,9,12,15,18,21,24-octaoxaheptacosan-27-oic acid (725 mg, 1.34 mmol, 1.0 equiv) in DMA (13.4 mL) was added DIPEA (0.7 mL, 4.0 mmol, 3.0 equiv) followed by PyBOP (905 mg, 1.74 mmol, 1.3 equiv). The reaction was stirred at room temperature for 2 h and then the reaction

mixture was concentrated under reduced pressure and the crude residue was purified by silica gel chromatography (0→20% MeOH/DCM) to afford the desired product (867 mg, 69% yield) as a colorless oil. LCMS (ESI) *m/z*: [M + H] calcd for $C_{46}H_{65}N_9O_{12}$: 936.49; found 936.3.

***Step 2*: Synthesis of 1-amino-27-(6-((4-amino-3-(2-aminobenzo[*d*]oxazol-5-yl)-1*H*-pyrazolo[3,4-*d*]pyrimidin-1-yl)methyl)-3,4-dihydroisoquinolin-2(1*H*)-yl)-3,6,9,12,15,18,21,24-octaoxaheptacosan-27-one (SI-36)**

To a solution of **SI-35** (0.9 g, 0.96 mmol, 1.0 equiv) in dioxane (3.2 mL) was added HCl (4 M in dioxane, 2.4 mL, 9.6 mmol, 10 equiv). The reaction stirred for 2 h and then was concentrated under reduced pressure to an oil. The oil was azeotroped with DCM (3 x 15 mL) to afford the crude desired product (881 mg, 105% yield, HCl) as a tan solid, which was used directly in the next step. LCMS (ESI) *m/z*: [M + H] calcd for $C_{41}H_{57}N_9O_{10}$: 836.43; found 836.3.

**(4-((2-aminoethyl)sulfonyl)-3-fluoro-2-methylphenyl)(7- (6-aminopyridin-3-yl)-2,3-dihydrobenzo[*f*][1,4]oxazepin-4(5*H*)-yl)methanone 2,2,2-trifluoroacetate (SI-42)**

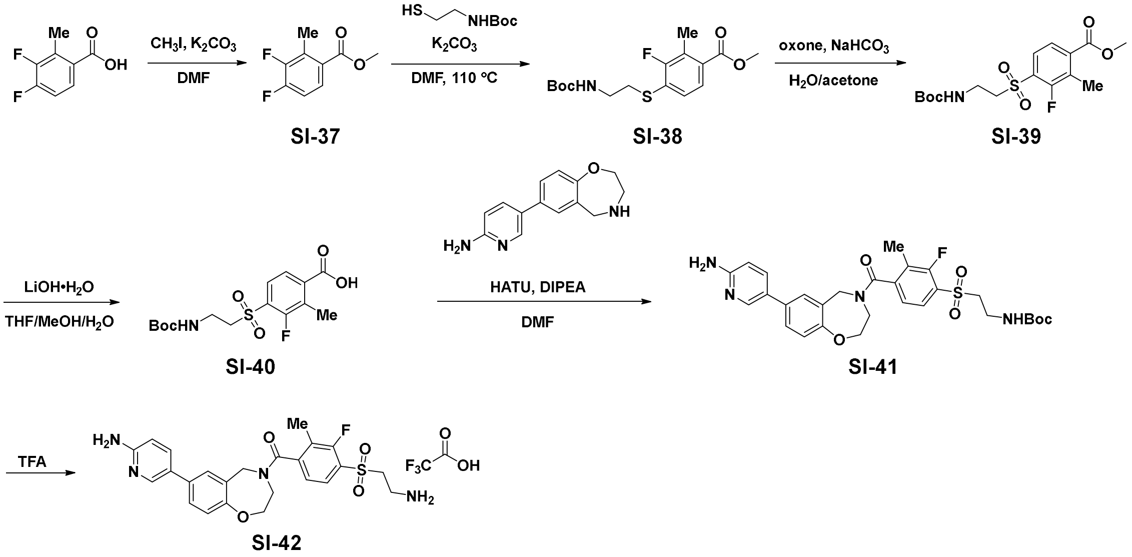

***Step 1*: Synthesis of methyl 3,4-difluoro-2-methylbenzoate (SI-37)**

To a solution of 3,4-difluoro-2-methylbenzoic acid (2 g, 11.62 mmol, 1.0 equiv) in DMF (20 mL) was added $K_2CO_3$ (4.82g, 34.86 mmol, 3.0 equiv) and iodomethane (3.26 mL, 52.29 mmol, 4.5 equiv) at room temperature. The mixture was stirred at room temperature for 3 h. The solution of methyl 3,4-difluoro-2-methylbenzoate in DMF (20 mL) was used directly in the next step.

***Step 2*: Synthesis of methyl 4-((2-((*tert*-butoxycarbonyl)amino)ethyl)thio)-3-fluoro-2-methylbenzoate (SI-38)**

To a solution of methyl 3,4-difluoro-2-methylbenzoate **SI-37** (2.16 g, 11.28 mmol, 1.0 equiv) in DMF (20 mL) was added *tert*-butyl (2-mercaptoethyl)carbamate (2.0 g, 11.28 mmol, 1 equiv) and $K_2CO_3$ (3.12 g, 22.56 mmol, 2.0 equiv) at room temperature. The reaction was stirred at 110 °C for 12 h, at which point the mixture was added to $H_2O$ (50 mL). The aqueous solution was then extracted with EtOAc (3 x 30 mL) and the organic phase was combined and concentrated under reduced pressure. The residue was purified by silica gel chromatography (0→25% EtOAc/pet. ether) to afford the desired product (3.0 g, 76% yield) as light yellow solid. $^1$H NMR (400 MHz, $CDCl_3$) δ 7.67 (d, *J* = 8.4 Hz, 1H), 7.23 (br t, *J* = 7.8 Hz, 1H), 4.92 (br s, 1H), 3.89 (s, 3H), 3.41 - 3.31 (m, 2H), 3.10 (br t, *J* = 6.4 Hz, 2H), 2.51 (d, *J* = 2.6 Hz, 3H), 1.45 (s, 9H).

***Step 3*: Synthesis of methyl 4-((2-((*tert*-butoxycarbonyl)amino)ethyl)sulfonyl)-3-fluoro-2-methylbenzoate (SI-39)**

To a solution of **SI-38** (3.3 g, 9.61 mmol, 1.0 equiv), NaOH (2 M, 4.80 mL, 1.0 equiv), and $NaHCO_3$ (2.42 g, 28.83 mmol, 3.0 equiv) in acetone (30 mL) was added potassium

peroxymonosulfate (12.35 g, 20.08 mmol, 2.1 equiv). The mixture was stirred for 12 h at room temperature and then the mixture was acidified to pH 5 by addition of 1N HCl. The aqueous layer was extracted with EtOAc (3 x 30 mL) and the combined organic phase was washed with brine (20 mL), dried with $Na_2SO_4$, filtered and concentrated under reduced pressure. The residue was purified by silica gel chromatography (0→25% EtOAc/pet. ether) to afford the desired product (2.1 g, 58% yield) as a yellow solid. LCMS (ESI) *m/z*: [M-56 + H] calcd for $C_{16}H_{22}FNO_6S$: 320.12; found 320.1. $^1$H NMR (400 MHz, $CDCl_3$) δ 7.83-7.80 (m, 2H), 5.10 (br s, 1H), 3.96 (s, 3H), 3.60 (br t, *J* = 5.6 Hz, 2H), 3.57-3.52 (m, 2H), 2.55 (d, *J* = 2.8 Hz, 3H), 1.41 (s, 9H).

***Step 4*: Synthesis of 4-((2-((*tert*-butoxycarbonyl)amino)ethyl)sulfonyl)-3-fluoro-2-methylbenzoic acid (SI-40)**

To a solution of **SI-39** (2.1 g, 5.59 mmol, 1.0 equiv) in THF (20 mL), MeOH (10 mL) and $H_2O$ (10 mL) was added LiOH•$H_2O$ (704.16 mg, 16.78 mmol, 3.0 equiv) at room temperature. The reaction mixture was stirred at 40 °C for 4 h. The mixture was then concentrated under reduced pressure to remove THF and MeOH. The aqueous phase was neutralized with 0.5N HCl and was then extracted with EtOAc (5 x 20 mL). The combined organic phase was washed with brine (2 x 20 mL), dried with $Na_2SO_4$, filtered and concentrated under reduced pressure to afford the desired product (2.01 g, 97% yield) as a white solid. LCMS (ESI) *m/z*: [M-100 + H] calcd for $C_{15}H_{20}FNO_6S$: 262.11; found 262.1. $^1$H NMR (400 MHz, DMSO-$d_6$) δ 7.79-7.70 (m, 2H), 3.58 (br t, *J* = 6.4 Hz, 2H), 3.26 (br d, *J* = 6.2 Hz, 2H), 2.44 (s, 3H), 1.27 (s, 9H).

***Step 5*: Synthesis of *tert*-butyl (2-((4-(7-(6-aminopyridin-3-yl)-2,3,4,5-tetrahydrobenzo[*f*][1,4]oxazepine-4-carbonyl)-2-fluoro-3-methylphenyl)sulfonyl)ethyl)carbamate (SI-41)**

To a solution of **SI-40** (690.08 mg, 1.91 mmol, 1.0 equiv) in DMF (10 mL) was added HATU (1.09 g, 2.86 mmol, 1.5 equiv) and DIPEA (1.66 mL, 9.55 mmol, 5 equiv). The reaction was stirred at room temperature for 30 min and then 5-(2,3,4,5-tetrahydrobenzo[*f*][1,4]oxazepin-7-yl)pyridin-2-amine (0.6 g, 1.91 mmol, 1.0 equiv, 2HCl) was added. The mixture was stirred for 2 h, at which point $H_2O$ (40 mL) was added. The mixture was stirred for 5 min and the resulting precipitate was collected by filtration to give the crude product. The residue was purified by silica gel chromatography (0→25% EtOAc/pet. ether) to afford the desired product (538 mg, 47% yield) as a light yellow solid. LCMS (ESI) *m/z*: [M + H] calcd for $C_{29}H_{33}FN_4O_6S$: 585.22; found 585.3.

***Step 6*: Synthesis of (4-((2-aminoethyl)sulfonyl)-3-fluoro-2-methylphenyl)(7-(6-aminopyridin-3-yl)-2,3-dihydrobenzo[*f*][1,4]oxazepin-4(5*H*)-yl)methanone 2,2,2-trifluoroacetate (SI-42)**

A solution **SI-41** (538 mg, 920.20 µmol, 1.0 equiv) in TFA (10.35 mL, 139.74 mmol, 151.85 equiv) was stirred at room temperature for 2 h. The solution was then concentrated under reduced pressure. The oily residue was triturated with MeCN (1 mL) and then dripped into MTBE (30 mL) for 10 min. The supernatant was removed and then the precipitate was collected by filtration under $N_2$ to afford the desired product (500 mg, 87% yield) as light brown solid. LCMS (ESI) *m/z*: [M + H] calcd for $C_{24}H_{25}FN_4O_4S$: 485.17; found 485.1.

**1-amino-*N*-(2-((4-(7-(6-aminopyridin-3-yl)-2,3,4,5-tetrahydrobenzo[*f*][1,4]oxazepine-4-carbonyl)-2-fluoro-3-methylphenyl)sulfonyl)ethyl)-3,6,9,12,15,18,21,24-octaoxaheptacosan-27-amide (SI-44)**

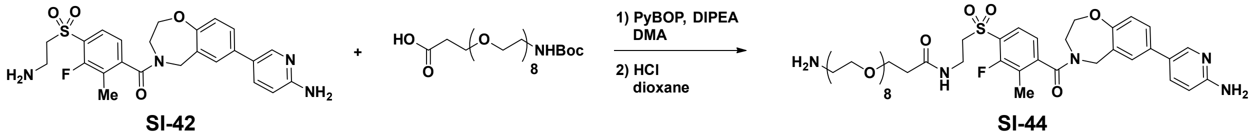


***Step 1*: Synthesis of *tert*-butyl (30-((4-(7-(6-aminopyridin-3-yl)-2,3,4,5-tetrahydrobenzo[*f*][1,4]oxazepine-4-carbonyl)-2-fluoro-3-methylphenyl)sulfonyl)-27-oxo-3,6,9,12,15,18,21,24-octaoxa-28-azatriacontyl)carbamate (SI-43)**

To a solution of **SI-42** (905 mg, 1.51 mmol) and 1-{[(*tert*-butoxy)carbonyl]amino}-3,6,9,12,15,18,21,24-octaoxaheptacosan-27-oic acid (834 mg, 1.54 mmol, 1.0 equiv) in DMA (3.8 mL) at 0 °C was added *N*-methylmorpholine (364 µL, 3.32 mmol, 2.2 equiv), HOBt (10.2 mg, 0.0755 mmol, 0.05 equiv), and ECDI (318 mg, 1.66 mmol, 1.1 equiv). The reaction mixture was warmed to room temperature and stirred for 3 h. The reaction mixture was then diluted with DCM (50 mL) and washed with 1:1 brine/$H_2O$ (100 mL), sat $NaHCO_3$ (100 mL), and then brine (100 mL). The organic layer was dried over $MgSO_4$, filtered, and concentrated under reduced pressure. Purification by silica gel chromatography (0→8% MeOH/DCM) afforded the desired product (673mg, 44% yield) as a colorless oil.

***Step 2:* Synthesis of 1-amino-*N*-(2-((4-(7-(6-aminopyridin-3-yl)-2,3,4,5-tetrahydrobenzo[*f*][1,4]oxazepine-4-carbonyl)-2-fluoro-3-methylphenyl)sulfonyl)ethyl)-3,6,9,12,15,18,21,24-octaoxaheptacosan-27-amide (SI-44)**

To a solution of **SI-43** (673 mg, 668 µmol, 1.0 equiv) in dioxane (2 mL) was added HCl (4 M in dioxane, 5 mL, 20 mmol, 30 equiv). The reaction mixture was stirred for 3 h and was

then concentrated under reduced pressure to afford the crude desired product (630 mg, 100% yield, HCl) as an oil, which was used directly in the next step. LCMS (ESI) *m/z*: [M + H] calcd for $C_{43}H_{62}FN_5O_{13}S$: 908.41; found 908.3.

**NMR Characterization**

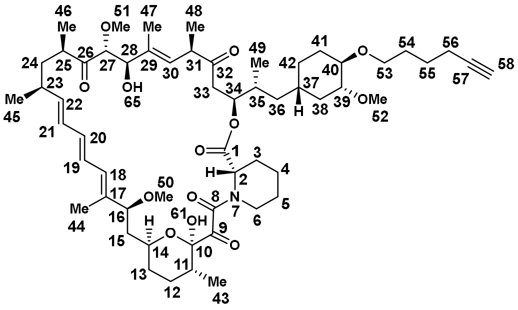

**Table S1.** NMR Analysis of **10**

| Atom | Atom Type | δ $^{1}$H Major (5:1) | δ $^{13}$C Major (5:1) | C to H HMBC | $^{1}$H-$^{1}$H COSY |
|---|---|---|---|---|---|
| 1 | *C*=O | N/A | 169.1 | 2 | N/A |
| 2 | *CH* | 4.94 (br d, 5.7 Hz) | 50.7 | n.d. | 3a,b |
| 3 | *CH2* | a: 2.10 (m)<br>b: 1.58 (m) | 26.3 | 2 | 3b, 4b, 2<br>3a, 2 |
| 4 | *CH2* | a: 1.66 (m)<br>b: 1.40 (m) | 20.3 | 2 | 4b<br>3a |
| 5 | *CH2* | a: 1.57 (m)<br>b: 1.29 (m) | 24.4 | n.d. | 5b, 6a<br>5a, 6b |
| 6 | *CH2* | a: 3.43(m)<br>b: 3.17 (m) | 43.4 | 2 | 6b, 5a<br>6a, 5b |
| 8 | *C*=O | N/A | 166.9 | 2 | N/A |
| 9 | *C*=O | N/A | 198.8 | 61 | N/A |
| 10 | O-*C*-OH | N/A | 98.9 | 43, 61 | N/A |
| 11 | *CH* | 2.03 (m) | 34.7 | 11, 43, 61 | 12 |
| 12 | *CH2* | 1.52 (2H, m) | 26.1 | 43 | 11 |
| 13 | *CH2* | a: 1.83 (m)<br>b: 1.18 (m) | 29.5 | 15b | 12 |
| 14 | *CH*-OC | 4.00 (m) | 66.1 | 15b | 13a,b, 15a,b |
| 15 | *CH2* | a: 1.85 (m)<br>b: 1.25 (m) | 40.0 | 16 | 15b, 16, 14<br>15a, 16, 14 |
| 16 | *CH*-OCH3 | 3.62 (dd, 12.2, 2.2) | 82.2 | 18, 44, 50 | 15a, 15b |

| | | | | | |
|---|---|---|---|---|---|
| 17 | -*C*= | N/A | 137.8 | 19, 44 | N/A |
| 18 | *CH*=C | 6.11 (d, 9.6 Hz) | 126.9 | 20, 44 | 19 |
| 19 | *CH*=C | 6.40 (dd, 14.7, 11.2 Hz) | 126.9 | 21 | 18, 20 |
| 20 | *CH*=C | 6.22 (dd, 14.7, 10.7 Hz) | 132.2 | 18, 22 | 19, 21 |
| 21 | *CH*=C | 6.13 (m) | 130.3 | 19 | 20, 22 |
| 22 | *CH*=C | 5.46 (dd, 15.0, 9.7 Hz) | 139.2 | 20, 24a, 45 | 21, 23 |
| 23 | *CH* | 2.22 (m) | 35.1 | 21, 25 | 24a,b, 45 |
| 24 | *CH2* | a: 1.40 (m)<br>b: 1.04 (m) | 39.4 | 45 | 23 |
| 25 | *CH* | 2.42 (m) | 39.3 | 46 | 24b, 46 |
| 26 | *C*=O | N/A | 210.4 | 24a, 25, 28, 46 | N/A |
| 27 | *CH*-OCH3 | 3.93 (d, 4.7 Hz) | 85.4 | 51, 65 | 28 |
| 28 | *CH*-OH | 4.01 (m) | 75.7 | 47 | 27, 65 |
| 29 | *C*=C | N/A | 137.1 | 47, 31 | N/A |
| 30 | *CH*=C | 5.09 (d, 10.1Hz) | 124.8 | 28, 31 ,47, 48 | 31 |
| 31 | *CH* | 3.27 (m) | 45.1 | 30, 48 | 48 |
| 32 | *C*=O | N/A | 207.4 | 31, 33b, 48 | N/A |
| 33 | *CH2* | a: 2.73 (m)<br>b: 2.38 (m) | 39.6 | n.d. | 34 |
| 34 | *CH*-OCO | 4.98 (m) | 73.5 | 36, 49 | 33a,b, 35 |
| 35 | *CH* | 1.68 (m) | 33.3 | 49 | 49 |
| 36 | *CH2* | a: 1.05 (m)<br>b: 0.97 (m) | 38.2 | 49 | 35 |
| 37 | *CH* | 1.27 (m) | 32.2 | 38b, 42b | n.d. |
| 38 | *CH2* | a: 1.89 (m)<br>b: 0.66 (m) | 36.0 | 36b | 38b<br>38a, 37 |
| 39 | *CH*-OCH3 | 2.42 (m) | 82.3 | 52 | 38a, b |
| 40 | *CH*-OR | 3.00 (m) | 82.0 | 53 | 41a, b |
| 41 | *CH2* | a: 1.94 (m)<br>b: 1.10 (m) | 29.5 | n.d. | 40, 41b<br>40, 41a, 42b |
| 42 | *CH2* | a: 1.56 (m)<br>b: 0.85 (m) | 30.8 | 36a | 42b<br>42a |
| 43 | 11-*CH3* | 0.73 (3H, d, 6.75 Hz) | 15.5 | 11 | 11 |
| 44 | 17-*CH3* | 1.63 (3H, s) | 10.4 | 16, 18 | N/A |
| 45 | 23-*CH3* | 0.98 (3H, d, 6.5 Hz) | 21.6 | 22 | 23 |
| 46 | 25-*CH3* | 0.83 (3H, d, 6.5 Hz) | 13.3 | 24a | 25 |
| 47 | 29-*CH3* | 1.74 (3H, s) | 13.3 | 30 | N/A |
| 48 | 31-*CH3* | 0.87 (3H, d, 6.5 Hz) | 15.5 | 30, 31 | 31 |
| 49 | 35-*CH3* | 0.78 (3H, d, 6.8 Hz) | 14.6 | 36a | 35 |
| 50 | 16-O*CH3* | 3.05 (3H, s) | 55.4 | 16 | N/A |
| 51 | 27-O*CH3* | 3.16 (3H, s) | 56.9 | 27 | N/A |
| 52 | 39-O*CH3* | 3.33 (3H, s) | 57.0 | 39 | N/A |
| 53 | *CH2*-O | a: 3.52 (m) | 68.2 | 40, 55 | 54 |

| | | | | | |
|---|---|---|---|---|---|
| | | b: 3.44 (m) | | | |
| 54 | *CH2* | 1.55 (2H, m) | 28.8 | 56 | 53, 55 |
| 55 | *CH2* | 1.49 (2H, m) | 24.8 | 53 | 54, 56 |
| 56 | *CH2*-C≡ | 2.16 (2H, m) | 17.4 | 54 | 55, 58 |
| 57 | -*C*≡CH | N/A | 85.4 | 55, 56 | N/A |
| 58 | ≡*CH* | 2.74 (m) | 71.0 | 56 | 56 |
| 61 | 10-O*H* | 6.44 (d, 1.3 Hz) | N/A | N/A | 11 |
| 65 | 28-O*H* | 5.25 (d, 4.4 Hz) | N/A | N/A | 28 |

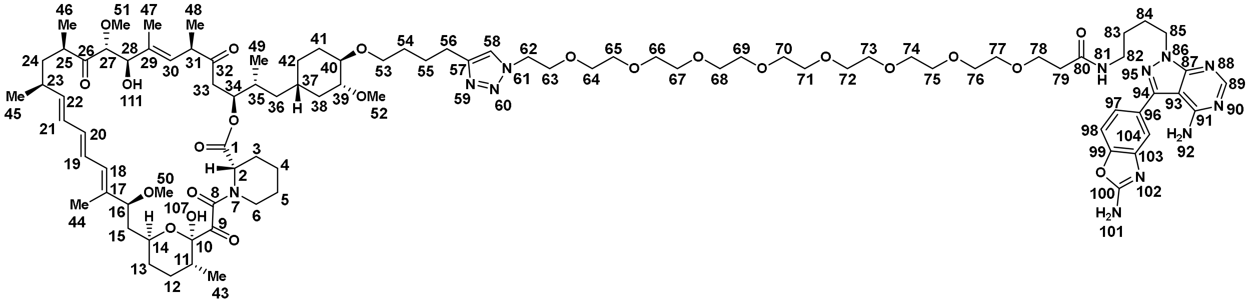

**Table S2.** NMR Analysis of **16**

| Atom | Atom Type | δ $^{1}$H Major (17:3) | δ $^{13}$C Major (17:3) | C to H HMBC | $^{1}$H-$^{1}$H COSY |
|---|---|---|---|---|---|
| 1 | *C*=O | N/A | 169.1 | 2 | N/A |
| 2 | *CH* | 4.94 (br d, 5.7 Hz) | 50.7 | 6a | 3a,b |
| 3 | *CH2* | a: 2.10 (br d, 12.8 Hz)<br>b: 1.66 (m) | 26.3 | 2 | 3b, 2<br>3a, 2, 4b |
| 4 | *CH2* | a: 1.67 (m)<br>b: 1.40 (m) | 20.3 | 2, 6a | 4b, 3a,b, 5b<br>4a, 3a,b |
| 5 | *CH2* | a: 1.57 (m)<br>b: 1.29 (m) | 24.4 | n.d. | 5b, 6a<br>4a, 5a, 6a,b |
| 6 | *CH2* | a: 3.43 (m)<br>b: 3.17 (m) | 43.4 | 2 | 5a,b, 6b<br>6a, 5b |
| 8 | *C*=O | N/A | 166.9 | 2 | N/A |
| 9 | *C*=O | N/A | 198.8 | 107 | N/A |
| 10 | O-*C*-OH | N/A | 98.9 | 107, 43, 11 | N/A |
| 11 | *CH* | 2.03 (m) | 34.7 | 107, 43 | 12, 43 |
| 12 | *CH2* | 1.51 (2H, m) | 26. 1 | 11, 13a, 43 | 11, 13a,b |
| 13 | *CH2* | a: 1.83 (m)<br>b: 1.18 (m) | 29.5 | 43, 15b | 13b, 12, 14<br>13a, 12, 14 |
| 14 | *CH*-OC | 4.00 (m) | 66.1 | 15b | 13a,b, 15a,b |
| 15 | *CH2* | a: 1.85 (m)<br>b: 1.25 (m) | 39.9 | 16 | 15b, 16, 14<br>15a, 16, 14 |

| | | | | | |
|---|---|---|---|---|---|
| 16 | *CH*-OCH3 | 3.62 (m) | 82.4 | 44, 50, 18 | 15a,b |
| 17 | -*C*= | N/A | 137.8 | 19, 44 | N/A |
| 18 | *CH*=C | 6.10 (d, 11.0 Hz) | 126.9 | 19, 44, 16, 20 | 19, 44 |
| 19 | *CH*=C | 6.40 (dd, 14.8, 11.0 Hz) | 126.9 | 18, 21 | 18, 20 |
| 20 | *CH*=C | 6.22 (dd, 14.8, 11.0 Hz) | 132.3 | 18, 22 | 19, 21 |
| 21 | *CH*=C | 6.13 (dd, 14.8, 11.0 Hz) | 130.3 | 19, 20 | 20, 22 |
| 22 | *CH*=C | 5.46 (dd, 14.8, 9.5 Hz) | 139.2 | 20, 45, 24a | 21, 23 |
| 23 | *CH* | 2.21 (m) | 35.1 | 21, 25, 45, 22, 24a | 22, 24b |
| 24 | *CH2* | a: 1.40 (m)<br>b: 1.04 (m) | 39.4 | 46, 45, 25, 22 | 24b, 25<br>23, 24a |
| 25 | *CH* | 2.41 (m) | 39.3 | 23, 46 | 24a, 46 |
| 26 | *C*=O | N/A | 210.4 | 27, 25, 46, 28, 24a | N/A |
| 27 | *CH*-OCH3 | 3.93 (d, 4.6 Hz) | 85.5 | 111, 51 | 28 |
| 28 | *CH*-OH | 4.00 (m) | 75.7 | 111, 47, 27, 30 | 27, 111 |
| 29 | *C*=C | N/A | 137.1 | 27, 31, 47, 28, 111 | N/A |
| 30 | *CH*=C | 5.09 (br d, 10.3 Hz) | 124.9 | 28, 47, 48, 31 | 31 |
| 31 | *CH* | 3.26 (m) | 45.1 | 48, 30 | 30, 48 |
| 32 | *C*=O | N/A | 207.4 | 30, 33a,b, 48, 31, 47 | N/A |
| 33 | *CH2* | a: 2.73 (br dd, 12.5, 2.3 Hz)<br>b: 2.38 (m) | 39.7 | n.d. | 34 |
| 34 | *CH*-OCO | 4.98 (dt, 7.7, 3.9, 3.9 Hz) | 73.5 | 33b, 36b, 49 | 35, 33 |
| 35 | *CH* | 1.67 (m) | 33.3 | 36a,b, 49, 33a | 34, 36a,b, 49 |
| 36 | *CH2* | a: 1.04 (m)<br>b: 0.96 (m) | 38.2 | 38b, 49 | 36b, 35,37<br>36a, 35, 37 |
| 37 | *CH* | 1.25 (m) | 32.2 | 36a,b, 42b, 49 | 36a,b, 38b, 42b |
| 38 | *CH2* | a: 1.89 (m)<br>b: 0.64 (q, 11.7, 11.7, 11.7 Hz) | 36.0 | 36a,b | 38b, 39<br>38a, 39 |
| 39 | *CH*-OCH3 | 2.95 (m) | 82.2 | 52, 38b | 38a,b, 40 |
| 40 | *CH*O- | 3.00 (m) | 82.0 | 53, 39 | 39, 41a,b |
| 41 | *CH2* | a: 1.94 (m)<br>b: 1.10 (m) | 29.5 | 37 | 40, 41b<br>40, 41a, 42b |
| 42 | *CH2* | a: 1.56 (m)<br>b: 0.84 (m) | 30.8 | 36a,b, 38b | 42b<br>42a, 41b, 37 |
| 43 | 11-*CH3* | 0.73 (3H, d, 6.8 Hz) | 15.5 | 11 | 11 |
| 44 | 17-*CH3* | 1.63 (3H, s) | 10.4 | 18, 16 | 18 |
| 45 | 23-*CH3* | 0.98 (3H, d, 6.6 Hz) | 21.6 | 22 | 23 |
| 46 | 25-*CH3* | 0.83 (3H, d, 6.5 Hz) | 13.3 | 25, 24a,b | 25 |
| 47 | 29-*CH3* | 1.73 (3H, s) | 13.3 | 30, 28 | 30 |

| | | | | | |
|---|---|---|---|---|---|
| 48 | 31-*CH3* | 0.87 (3H, d, 6.6 Hz) | 15.5 | 30, 31 | 31 |
| 49 | 35-*CH3* | 0.77 (3H, d, 6.8 Hz) | 14.6 | 36a,b | 35 |
| 50 | 16-O*CH3* | 3.05 (3H, s) | 55.4 | 16 | N/A |
| 51 | 27-O*CH3* | 3.15 (3H, s) | 56.9 | 27 | N/A |
| 52 | 39-O*CH3* | 3.31 (3H, s) | 57.0 | 39 | N/A |
| 53 | O*CH2* | 3.51 (2H, m) | 68.5 | 55, 54, 39 | 54 |
| 54 | *CH2* | 1.51 (2H, m) | 29.3 | 55, 56, 53 | 53 |
| 55 | *CH2* | 1.61 (2H, m) | 25.7 | 53, 56, 54 | N/A |
| 56 | *CH2*-triazole | 2.60 (2H, t, 6.5 Hz) | 24.7 | 55, 54 | N/A |
| 57 | N*C*=C | N/A | 146.6 | 58, 56, 55 | N/A |
| 58 | *CH*=N | 7.78 (s) | 122.0 | 62, 56 | N/A |
| 62 | *CH2*-triazole | 4.45 (2H, t, 5.3 Hz) | 49.1 | 58, 63 | 63 |
| 63 | *CH2*N | 3.78 (2H, t, 5.3 Hz) | 68.7 | 64, 62 | 62 |
| 64-77 | O-(*CH2CH2*O)$_7$ | 3.48 (28H, m) | 69.7 | multi | multi |
| 78 | O*CH2* | 3.56 (2H, t, 6.5 Hz) | 66.8 | 77, 79 | 79 |
| 79 | *CH2*C=O | 2.26 (2H, t, 6.5 Hz) | 36.1 | 78 | 78 |
| 80 | *C*=O | N/A | 169.8 | 81, 78, 82, 79 | N/A |
| 81 | N*H*C=O | 7.79 (m) | N/A | N/A | 82 |
| 82 | *CH2* | 3.06 (2H, m) | 37.8 | 81, 84, 83 | 81, 83 |
| 83 | *CH2* | 1.38 (2H, m) | 26.2 | 85, 84, 81, 82 | 82, 84 |
| 84 | *CH2* | 1.84 (2H, m) | 26.5 | 85, 82, 83 | 85, 83 |
| 85 | *CH2*N | 4.33 (2H, t, 5.3 Hz) | 45.8 | 84, 83 | 84 |
| 87 | PP-*C* | N/A | 153.9 | 89, 85 | N/A |
| 89 | PP-*CH* | 8.24 | 155.5 | n.d. | N/A |
| 91 | PP-*C* | N/A | 158.0 | 89 | N/A |
| 92 | PP-N*H2* | 6.74 (2H, br s) | N/A | N/A | N/A |
| 93 | PP-*C* | N/A | 97.1 | 89 | N/A |
| 94 | PP-*C* | N/A | 143.9 | 104, 97 | N/A |
| 96 | BO-*C* | N/A | 128.5 | 98 | N/A |
| 97 | BO-*CH* | 7.24 (dd, 8.1, 1.6 Hz) | 120.3 | 104 | 98, 104 |
| 98 | BO-*CH* | 7.46 (d, 8.1 Hz) | 108.7 | 97, 104 | 97 |
| 99 | BO-*C* | N/A | 148.2 | 97, 98, 104 | N/A |
| 100 | BO-*C*-NH2 | N/A | 163.3 | 101 | N/A |
| 101 | BO-N*H2* | 7.51 (2H, s) | N/A | N/A | N/A |
| 103 | BO-*C*- | N/A | 144.3 | 98 | N/A |
| 104 | BO-*CH* | 7.41 (d, 1.6 Hz) | 114.9 | 97, 98 | 97 |
| 107 | 10-O*H* | 6.44 (br s) | N/A | N/A | N/A |
| 111 | 28-O*H* | 5.25 (d, 5.1 Hz) | N/A | N/A | 28 |

PP stands for pyrazolo[3,4-d]pyrimidine

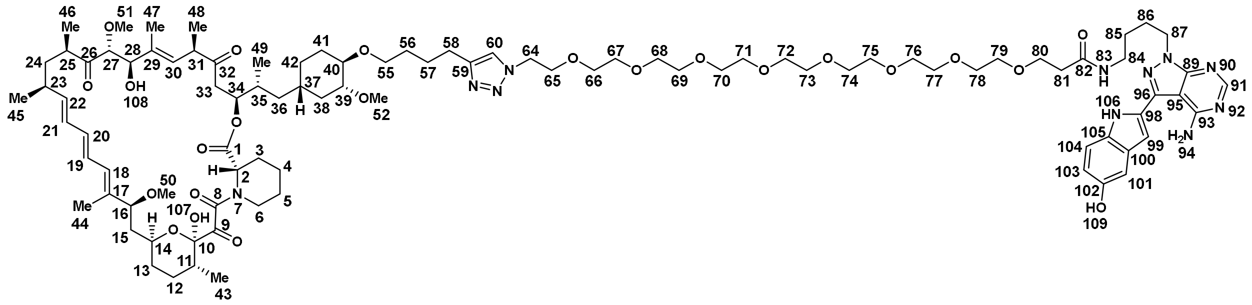

**Table S3.** NMR Analysis of **18**

| Atom | Atom Type | δ $^1$H Major (5:1) | δ $^{13}$C Major (5:1) | C to H HMBC | $^1$H-$^1$H COSY |
|---|---|---|---|---|---|
| 1 | *C*=O | N/A | 169.2 | 2 | N/A |
| 2 | *CH* | 4.94 (d, 5.6 Hz) | 50.7 | n.d. | 3a,b |
| 3 | *CH2* | a: 2.08 (m)<br>b: 1.66 (m) | 26.3 | 2 | 2, 4a |
| 4 | *CH2* | a: 1.66 (m)<br>b: 1.40 (m) | 20.3 | 2 | 3a, 5b |
| 5 | *CH2* | a: 1.58 (m)<br>b: 1.29 (m) | 24.4 | 3 | 4a, 6a,b |
| 6 | *CH2* | a: 3.43 (m)<br>b: 3.18 (m) | 43.4 | 2 | 5a,b |
| 8 | *C*=O | N/A | 166.9 | 2 | N/A |
| 9 | *C*=O | N/A | 198.8 | 107 | N/A |
| 10 | O-*C*-OH | N/A | 98.9 | 11, 43, 107 | N/A |
| 11 | *CH* | 2.03 (m) | 34.7 | 10, 107 | 12, 43 |
| 12 | *CH2* | 1.51 (2H, m) | 26.1 | 11, 13a | 11, 13a,b |
| 13 | *CH2* | a: 1.85(m)<br>b: 1.17 (m) | 29.5 | 43, 15a,b | 12, 14 |
| 14 | *CH*-OC | 4.00 (m) | 66.1 | 12, 15a,b | 15b |
| 15 | *CH2* | a: 1.86 (m)<br>b: 1.26 (m) | 39.4 | n.d. | 14 |
| 16 | *CH*-OCH3 | 3.62 (m) | 82.2 | 18, 44, 50 | 15a |
| 17 | -*C*= | N/A | 137.8 | 19, 44 | N/A |
| 18 | *CH*=C | 6.10 (d, 9.6 Hz) | 126.9 | 16, 19, 20, 44 | 19 |
| 19 | *CH*=C | 6.40 (dd, 14.5, 11.3 Hz) | 126.9 | 18, 20, 21 | 18, 2 |
| 20 | *CH*=C | 6.22 (m) | 132.3 | 18, 22 | 19, 21 |
| 21 | *CH*=C | 6.13 (m) | 130.3 | 19 | 20, 22 |
| 22 | *CH*=C | 5.46 (dd, 15.0, 9.7 Hz) | 139.2 | 20 | 21, 23 |
| 23 | *CH* | 2,22 (m) | 35.1 | 21, 22, 24a, 25, 45 | 24a, 45 |
| 24 | *CH2* | a: 1.40 (m)<br>b: 1.04 (m) | 39.4 | 22, 25, 45, 46 | 25 |

| | | | | | |
|---|---|---|---|---|---|
| 25 | *CH* | 2.41 (m) | 39.4 | 24a, 46 | 24b, 46 |
| 26 | *C*=O | N/A | 210.4 | 24a, 25, 28, 46 | N/A |
| 27 | *CH*-OCH3 | 3.93 (2H, d, 4.6 Hz) | 85.4 | 28, 51 | 28 |
| 28 | *CH*-OH | 4.00 (m) | 75.7 | 27, 30, 47, 108 | 27 |
| 29 | *C*=C | N/A | 137.0 | 27, 28, 31, 47 | N/A |
| 30 | *CH*=C | 5.09 (d, 10.0 Hz) | 124.8 | 28, 31, 47, 48 | 31, 47 |
| 31 | *CH* | 3.26 (m) | 45.1 | 30, 48 | 30, 48 |
| 32 | *C*=O | N/A | 207.4 | 30, 31, 33a,b, 48 | N/A |
| 33 | *CH2* | a: 2.72 (m)<br>b: 2.36 (m) | 39.4 | n.d. | 34 |
| 34 | *CH*-OCO | 4.98 (m) | 73.5 | 33b, 36a, 49 | 33a,b, 35 |
| 35 | *CH* | 1.67 (m) | 33.3 | 36a,b, 49 | 34, 36b, 49 |
| 36 | *CH2* | a: 1.04 (m)<br>b: 0.96 (m) | 38.2 | 38a,b, 49 | 35, 37 |
| 37 | *CH* | 1.25 (m) | 32.2 | 36a,b, 38b, 42b | 36a, 38b, 42b |
| 38 | *CH2* | a: 1.89 (m)<br>b: 0.65 (m) | 36.0 | 36a,b | 39 |
| 39 | *CH*-OCH3 | 2.95 (m) | 82.3 | 38b, 52 | 38b |
| 40 | *CH*-OR | 3.00 (m) | 82.0 | 38b, 41b, 52, 55a,b | 41a,b |
| 41 | *CH2* | a: 1.92 (m)<br>b: 1.10 (m) | 29.5 | 37 | 40, 42b |
| 42 | *CH2* | a: 1.54 (m)<br>b: 0.83 (m) | 30.8 | 36a, 38b | 37, 41a,b |
| 43 | 11-*CH3* | 0.74 (3H, d, 6.6 Hz) | 15.5 | 11 | 11 |
| 44 | 17-*CH3* | 1.63 (3H, s) | 10.4 | 16, 18 | N/A |
| 45 | 23-*CH3* | 0.98 (3H, d, 6.5 Hz) | 21.6 | 22 | 23 |
| 46 | 25-*CH3* | 0.83 (3H, d, 6.3 Hz) | 13.3 | 24, 25 | 25 |
| 47 | 29-*CH3* | 1.73 (3H, s) | 13.3 | 28, 30 | N/A |
| 48 | 31-*CH3* | 0.87 (3H, d, 6.5 Hz) | 15.5 | 30, 31, 47 | 31 |
| 49 | 35-*CH3* | 0.77 (3H, d, 6.6 Hz) | 14.6 | 34, 35, 36a | 35 |
| 50 | 16-O*CH3* | 3.05 (3H, s) | 55.4 | 16 | N/A |
| 51 | 27-O*CH3* | 3.15 (3H, s) | 56.9 | 27 | N/A |
| 52 | 39-O*CH3* | 3.31 (3H, s) | 57.0 | 39, 40 | N/A |
| 55 | -*CH2*-O | a: 3.50 (m)<br>b: 3.45 (m) | 68.5 | 40, 56, 57 | 56 |
| 56 | *CH2* | 1.50 (2H, m) | 29.3 | 55, 57,58 | 55, 57 |
| 57 | *CH2* | 1.64 (2H, m) | 25.7 | 55, 56, 58 | 56, 58 |
| 58 | *CH2*-triazole | 2.60 (2H, t, 7.4 Hz) | 24.7 | 57 | 57 |
| 59 | N-*C*-C= | N/A | 146.5 | 57,58,60 | N/A |
| 60 | *CH*=C | 7.78 (s) | 122.0 | 58, 64 | N/A |
| 64 | *CH2*-triazole | 4.45 (2H, t, 5.4 Hz) | 49.1 | 65 | 65 |
| 65 | *CH2*-O | 3.78 (2H, t, 5.2 Hz) | 68.7 | 64 | 64 |

| | | | | | |
|---|---|---|---|---|---|
| 66-79 | O-(*CH2CH2*O)$_7$ | 3.42-3.48 (28H, m) | 69.6 | multi | multi |
| 80 | -*CH2*-O | 3.56 (2H, t, 6.6 Hz) | 66.7 | 79, 81 | 81 |
| 81 | -*CH2*-C=O | 2.26 (2H, t, 6.4 Hz) | 36.1 | 80 | 80 |
| 82 | N-*C*=O | N/A | 169.8 | 80, 81, 83, 84 | N/A |
| 83 | N*H* | 7.79 (t, 5.6 Hz) | N/A | N/A | 84 |
| 84 | *CH2*-NH-CO | 3.08 (2H, m) | 37.8 | 83, 85, 86 | 83, 85 |
| 85 | *CH2* | 1.37 (2H, m) | 26.2 | 84, 86, 87 | 84, 86 |
| 86 | *CH2* | 1.87 (2H, m) | 26.5 | 84, 85, 87 | 85, 87 |
| 87 | *CH2*-N | 4.36 (2H, t, 6.8 Hz) | 45.9 | 85, 86 | 86 |
| 89 | PP-*C* | N/A | 153.9 | 87, 91 | N/A |
| 91 | PP-*CH* | 8.26 (s) | 155.6 | n.d. | N/A |
| 93 | PP-*C* | N/A | 157.9 | 91 | N/A |
| 94 | PP-N*H2* | 7.09 (2H, bs) | N/A | N/A | N/A |
| 95 | PP-*C* | N/A | 97.2 | 91 | N/A |
| 96 | PP-*C* | N/A | 136.9 | 99 | N/A |
| 98 | Indolol-*C* | N/A | 130.4 | 99, 106 | N/A |
| 99 | Indolol-*CH* | 6.67 (1H, s) | 101.2 | 101, 106 | N/A |
| 100 | Indolol-*C* | N/A | 131.4 | 99, 101, 106 | N/A |
| 101 | Indolol-*CH* | 6.92 (d, 2.2 Hz) | 104.0 | 99, 103, 104, 109 | 103 |
| 102 | Indolol-*C* | 6.68 (dd, 8.6 ,2.2 Hz) | 151.0 | 101, 103, 104, 109 | N/A |
| 103 | Indolol-*CH* | 6.68 (dd, 8.6 ,2.2 Hz) | 112.7 | 101, 109 | 101, 104 |
| 104 | Indolol-*CH* | 7.25 (d, 8.6 Hz) | 112.0 | n.d. | 103 |
| 105 | Indolol-*C* | N/A | 129.0 | N/A | N/A |
| 106 | Indolol-N*H* | 11.31 (s) | N/A | N/A | 99 |
| 107 | 10-O*H* | 6.43 (s) | N/A | N/A | N/A |
| 108 | 28-O*H* | 5.25 (s) | N/A | N/A | 28 |
| 109 | Indolol-O*H* | 8.72 (s) | N/A | N/A | N/A |

PP stands for pyrazolo[3,4-d]pyrimidine

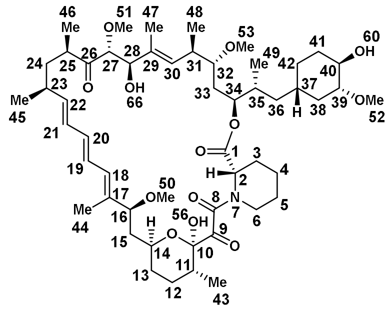


**Table S4.** NMR Analysis of **12**

| Atom | Atom Type | δ [1]H Major (5:1) | δ [13]C Major (5:1) | C to H HMBC | $^{1}$H-$^{1}$H COSY |
|---|---|---|---|---|---|
| 1 | *C*=O | N/A | 169.4 | 2, 3b, 34 | N/A |
| 2 | *CH* | 5.02 (d, 6.2 Hz) | 49.6 | 6b | 3b |
| 3 | *CH2* | a: 1.94 (m)<br>b: 1.67 (m) | 26.7 | 2 | n.d. |
| 4 | *CH2* | a: 1.69 (m)<br>b: 1.38 (m) | 20.2 | 2, 3b, 6b | n.d. |
| 5 | *CH2* | a: 1.62 (m)<br>b: 1.30 (m) | 24.4 | 6a,b | n.d. |
| 6 | *CH2* | a: 3.59 (m)<br>b: 3.42 (m) | 43.0 | 2 | 5a,b |
| 8 | *C*=O | N/A | 166.8 | 2, 6b | N/A |
| 9 | *C*=O | N/A | 200.2 | 56 | N/A |
| 10 | O-*C*-OH | N/A | 98.9 | 11, 43, 56 | N/A |
| 11 | *CH* | 2.03 (m) | 35.1 | 43, 56 | 12, 43 |
| 12 | *CH2* | 1.49 (2H, m) | 25.9 | 11, 43 | 11, 13b |
| 13 | *CH2* | a: 1.76 (m)<br>b: 1.06 (m) | 29.4 | 15a,b, 12 | n.d. |
| 14 | *CH*-OC | 3.91 (m) | 66.1 | 13b, 15a,b, 16 | 13b, 15a,b |
| 15 | *CH2* | a: 1.94 (m)<br>b: 1.07 (m) | 40.2 | 16 | 14, 16 |
| 16 | *CH*-OCH3 | 3.55 (m) | 82.5 | 18, 44, 50 | 15a,b |
| 17 | -*C*= | N/A | 138.1 | 19, 44 | N/A |
| 18 | *CH*=C | 6.04 (d, 11.0 Hz) | 127,0 | 16, 20 | 19 |
| 19 | *CH*=C | 6.44 (m) | 126.8 | 18 | 18, 20 |
| 20 | *CH*=C | 6.22 (dd, 14.7, 10.6 Hz) | 132.3 | 12, 22 | 19, 21 |
| 21 | *CH*=C | 6.14 (dd, 14.7, 10.4 Hz) | 130.4 | 19 | 20, 22 |
| 22 | *CH*=C | 5.48 (dd, 14.7, 9.7 Hz) | 139.7 | 20, 24a,b, 45 | 21, 23 |
| 23 | *CH* | 2.20 (m) | 35.5 | 21, 22, 24a, 45 | 24a, 45 |
| 24 | *CH2* | a: 1.42 (m)<br>b: 1.07 (m) | 40,0 | 25, 45, 46 | 24b, 25 |

| | | | | | |
|---|---|---|---|---|---|
| 25 | *CH* | 2.64 (m) | 37.3 | 27, 46 | 24b, 46 |
| 26 | *C*=O | N/A | 211.8 | 24a, 25, 27, 28, 46 | N/A |
| 27 | *CH*-OCH3 | 3.56 (m) | 86.7 | 28, 60, 51 | 28 |
| 28 | *CH*-OH | 3.94 (m) | 75.8 | 27, 47, 60 | 27, 60 |
| 29 | *C*=C | N/A | 134.3 | 27, 28, 31, 47, 60 | N/A |
| 30 | *CH*=C | 5.04 (m) | 129.7 | 28, 31, 32, 47, 48 | 31, 47 |
| 31 | *CH* | 2.49 (m) | 35,0 | 30, 48 | 30, 32, 48 |
| 32 | *CH*-OCH3 | 2.86 (m) | 84.0 | 31, 33b, 48, 53 | 31, 33a,b |
| 33 | *CH2* | a: 1.57 (m)<br>b: 1.49 (m) | 30.4 | 31 | n.d. |
| 34 | *CH*-OCO | 4.73 (m) | 78.1 | 32, 33b, 49 | 33a,b, 35 |
| 35 | *CH* | 1.65 (m) | 33.9 | 49 | 36b |
| 36 | *CH2* | a: 1.10 (m)<br>b: 1.02 (m) | 38.1 | 49 | 35 |
| 37 | *CH* | 1.29 (m) | 32.6 | 38b | 38b |
| 38 | *CH2* | a: 1.94 (m)<br>b: 0.62 (q, 12.0 Hz) | 35.3 | n.d. | 37, 39 |
| 39 | *CH*-OCH3 | 2.87 (m) | 83.7 | 38b, 52 | 38a,b, 40 |
| 40 | *CH*-OH | 3.22 (m) | 73.2 | 38b, 60 | 39, 41a,b, 66 |
| 41 | *CH2* | a: 1.77 (m)<br>b: 1.19 (m) | 33,0 | 60 | n.d. |
| 42 | *CH2* | a: 1.53 (m)<br>b: 0.90 (m) | 31.4 | 38b | 41a,b |
| 43 | 11-*CH3* | 0.72 (3H, d, 6.8 Hz) | 15.2 | 11 | 11 |
| 44 | 17-*CH3* | 1.7 (3H, s) | 10.3 | 16, 18 | N/A |
| 45 | 23-*CH3* | 0.97 (3H, d, 6.5 Hz) | 21.8 | 22 | 23 |
| 46 | 25-*CH3* | 0.89 (3H, d, 6.5 Hz) | 14.6 | 24a, 25 | 25 |
| 47 | 29-*CH3* | 1.54 (3H, s) | 12,0 | 28, 30 | N/A |
| 48 | 31-*CH3* | 0.85 (3H, d, 6.8 Hz) | 17.0 | 30, 31, 32 | 31 |
| 49 | 35-*CH3* | 0.79 (3H, d, 6.8 Hz) | 14.9 | 34, 36a,b | 35 |
| 50 | 16-O*CH3* | 3.04 (3H, s) | 55.3 | 16 | N/A |
| 51 | 27-O*CH3* | 3.12 (3H, s) | 57.3 | 27 | N/A |
| 52 | 39-O*CH3* | 3.34 (3H, s) | 56.7 | 39 | N/A |
| 53 | 32-O*CH3* | 3.09 (3H, s) | 54.7 | 32 | N/A |
| 56 | 10-O*H* | 6.44 (m) | N/A | N/A | N/A |
| 60 | 40-O*H* | 5.08 (d, 4.6 Hz) | N/A | N/A | 40 |
| 66 | 28-O*H* | 4.60 (d, 4.4 Hz) | N/A | N/A | 28 |

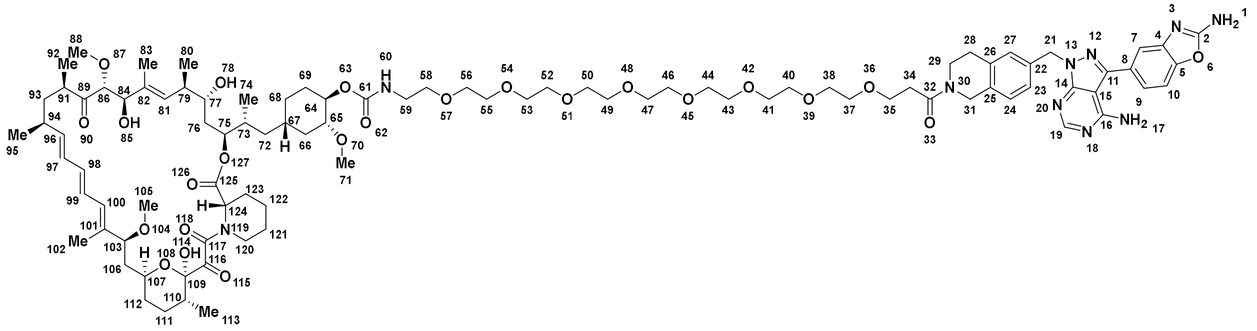

**Table S5.** NMR Analysis of RMC-5552 **38**

| Atom | Atom Type | δ [1]H | δ [13]C / type |
|---|---|---|---|
| 2 | *C* | N/A | 163.0 |
| 4 | *C* | N/A | 143.7 |
| 5 | *C* | N/A | 149.1 |
| 7 | *CH* | 7.58, br s | 116.1 |
| 8 | *C* | N/A | 128.5 |
| 9 | *CH* | 7.34, o | 121.6 |
| 10 | *CH* | 7.35, o | 109.6 |
| 11 | *C* | N/A | 145.1 |
| 14 | *C* | N/A | 154.0 |
| 15 | *C* | N/A | 98.4 |
| 16 | *C* | N/A | 157.7 |
| 19 | *CH* | 8.37, m | 155.5 |
| 21 | *CH2* | 5.56, s | 50.4 |
| 22 | *C* | N/A | 135.1 |
| 23 | *CH* | 7.23, d, 7.8; 7.21, d, 7.84 | 126.4 |
| 24 | *CH* | 7.07, d, 8.05; 7.02, d, 8.05 | 126.9 |
| 25 | *C* | N/A | 133.2 |
| 26 | *C* | N/A | 134.9 |
| 27 | *CH* | 7.15, br s; 7.17, br s | 128.0 |
| 28 | *CH2* | 2.83, o; 2.78, o | 29.4 |
| 29 | *CH2* | 3.65, o | 43.2 |
| 31 | *CH2* | 4.60, s; 4.66, s | 44.0 |
| 32 | *C* | N/A | 170.0 |
| 34 | *CH2* | 2.69, m | 33.8 |
| 35 | *CH2* | 3.78, o | 67.2 |
| 37/38/ 40/41/ | *CH2* | 3.60, o | 70.5 |

| | | | |
|---|---|---|---|
| 43/44/<br>46/47/<br>49/50/<br>52/53/<br>55/56 | | | |
| 58 | *CH2* | 3.56, o | 70.5 |
| 59 | *CH2* | 3.36, br o | 40.8 |
| 61 | *C* | N/A | 156.3 |
| 64 | *CH* | 4.56, m | 76.7 |
| 65 | *CH* | 3.09, m | 81.1 |
| 66 | *CH2* | 2.07, o | 35.8 |
| 67 | *CH* | 1.36, o | 32.9 |
| 68 | *CH2* | 1.62, o; 1.03, o | 31.5 |
| 69 | *CH2* | 2.04, o; 1.29, o | 30.3 |
| 71 | *CH3* | 3.35, o | 57.3 |
| 72 | *CH2* | 1.25, o; 1.14, o | 38.0 |
| 73 | *CH* | 1.83, o | 33.5 |
| 74 | *CH3* | 0.92, o | 16.1 |
| 75 | *CH* | 4.99, (m) | 78.8 |
| 76 | *CH2* | 1.63, o; 1.73, o | 35.2 |
| 77 | *CH* | 3.47, o | 73.5 |
| 79 | *CH* | 2.38, o | 38.9 |
| 80 | *CH3* | 0.96, o | 15.6 |
| 81 | *CH* | 5.31, o | 130.8 |
| 82 | *C* | N/A | 133.4 |
| 83 | *CH3* | 1.64, o | 16.1/ |
| 84 | *CH* | 4.14, m | 78.8 |
| 86 | *CH* | 3.64, o | 85.3 |
| 88 | *CH3* | 3.32, o | 57.3 |
| 89 | *C* | N/A | 216.4 |
| 91 | *CH* | 2.81, o | 40.9 |
| 92 | *CH3* | 0.99, o | 13.9 |
| 93 | *CH2* | 1.46, o; 1.19, o | 40.1 |
| 94 | *CH* | 2.30, o | 35.3 |
| 95 | *CH3* | 1.02, o | 21.8 |
| 96 | *CH* | 5.52, o | 140.0 |
| 97 | *CH* | 6.12, o | 130.3 |
| 98 | *CH* | 6.32, o | 133.8 |
| 99 | *CH* | 6.36, o | 126.4 |
| 100 | *CH* | 5.94, o | 129.5 |
| 101 | *C* | N/A | 135.4 |

| | | | |
|---|---|---|---|
| 102 | *CH3* | 1.66, o | 10.1 |
| 103 | *CH* | 3.64, o | 84.4 |
| 105 | *CH3* | 3.12, o | 55.8 |
| 106 | *CH2* | 1.85, o; 1.54, o | 38.9 |
| 107 | *CH* | 3.85, o | 67.2 |
| 109 | *C* | N/A | 98.5 |
| 110 | *CH* | 1.99, o | 33.5 |
| 111 | *CH2* | 1.58, o | 27.1 |
| 112 | *CH2* | 1.31, o | 31.2 |
| 113 | *CH3* | 0.93, o | 16.1 |
| 116 | *C* | N/A | 193.1 |
| 117 | *C* | N/A | 167.0 |
| 120 | *CH2* | 3.54, o | 44.2 |
| 121 | *CH2* | 1.71, o, 1.44, o | 25.2 |
| 122 | *CH2* | 2.31, o; 1.75, o | 26.9 |
| 123 | *CH2* | 1.77, o | 20.6 |
| 124 | *CH* | 5.29, o | 51.3 |
| 125 | *C* | N/A | 170.1 |

**Figure S1.** HPLC trace for RMC-5552 **38**

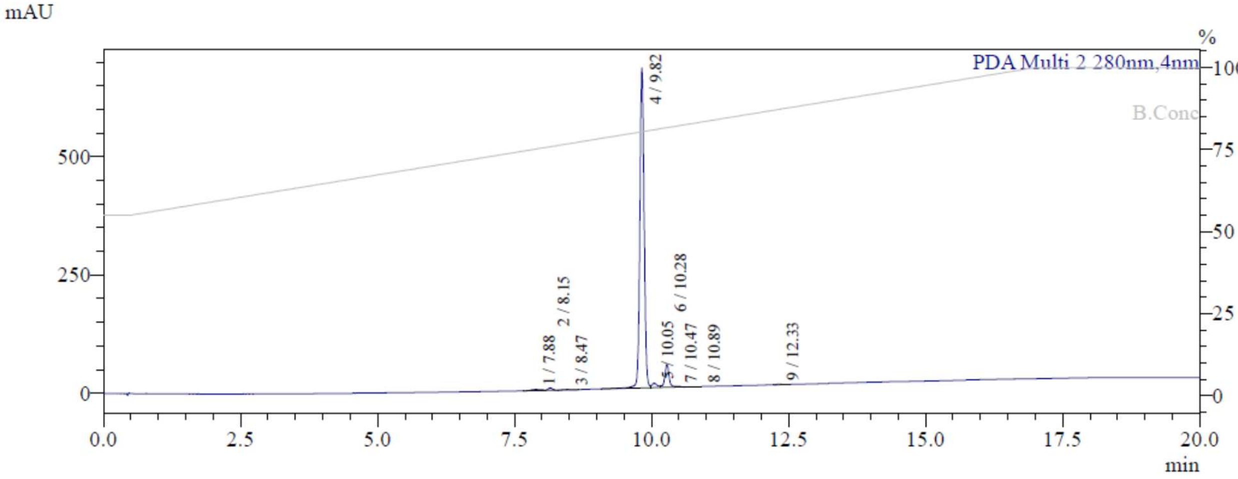

Peak Table

PDA Ch2 280nm

| Peak# | Ret. Time | Area | Area% |
|---|---|---|---|
| 1 | 7.881 | 27309 | 0.664 |
| 2 | 8.152 | 26736 | 0.650 |
| 3 | 8.473 | 5610 | 0.136 |
| 4 | 9.825 | 3711660 | 90.231 |
| 5 | 10.049 | 60418 | 1.469 |
| 6 | 10.281 | 259086 | 6.298 |
| 7 | 10.472 | 11058 | 0.269 |
| 8 | 10.890 | 4163 | 0.101 |
| 9 | 12.327 | 7452 | 0.181 |
| Total | | 4113493 | 100.000 |

RMC-5552 **38** (9.82 min) and oxapane isomer (10.281 min, 1.05 RRT) with overall purity of 96.5%

**Figure S2.** HPLC trace for RMC-6272 **40**

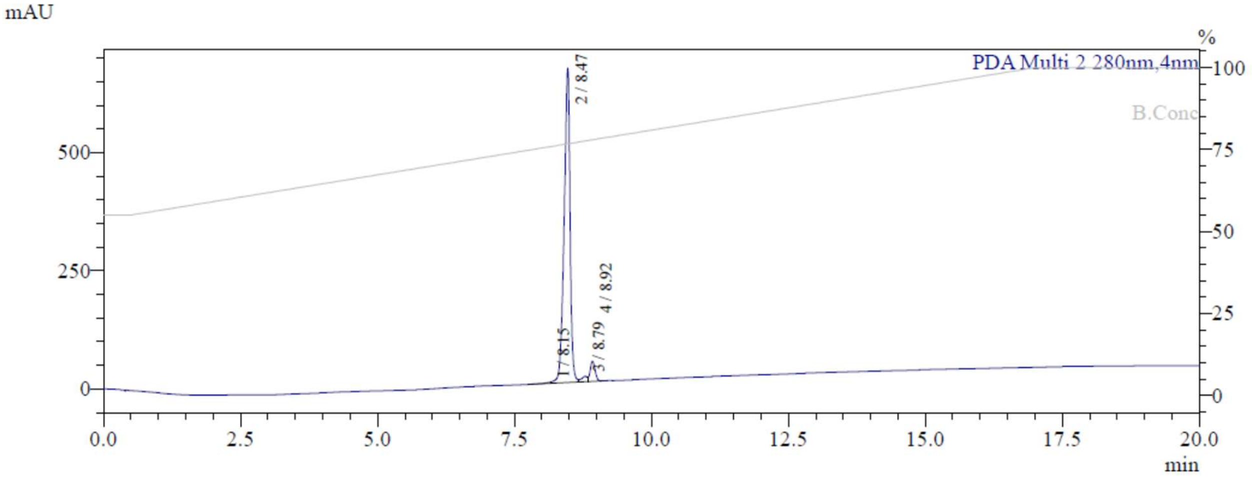


Peak Table

PDA Ch2 280nm

| Peak# | Ret. Time | Area | Area% |
|---|---|---|---|
| 1 | 8.146 | 16790 | 0.313 |
| 2 | 8.473 | 4997203 | 93.074 |
| 3 | 8.792 | 86761 | 1.616 |
| 4 | 8.923 | 268333 | 4.998 |
| Total | | 5369087 | 100.000 |

RMC-6272 **40** (8.473 min) and oxapane isomer (8.923 min, 1.05 RRT) with overall purity of 98.1%

**Table S6.** Data collection, processing, and refinement statistics for **11** structure

| | |
|---|---|
| Ligand | **11** |
| PDB Code | 8ER6 |
| X-ray source | PXII/X10SA (SLS[1]) |
| Wavelength [Å] | 0.9999 |
| Detector | PILATUS 6M |
| Temperature [K] | 100 |
| Space group | P 64 2 2 |
| Cell: a; b; c; [Å], α; β; γ; [◦] | 125.31; 125.31; 252.85, 90.0; 90.0; 120.0 |
| Resolution [Å] | 108.52 - 2.81 (3.06-2.81) |
| Unique reflections | 28386 (6418) |
| Multiplicity | 5.4 (5.5) |
| Completeness [%] | 96.5 (99.0) |
| Rsym [%] | 7.1 (44.8) |
| Rmeas [%] | 7.7 (48.9) |
| Mean(I)/sd | 15.55 (3.48) |
| Number of reflections (working /test) | 27717 / 669 |
| Rcryst [%] | 19.8 |
| Rfree[%] | 23.1 |
| Total number of atoms: | |
| Protein | 4922 |
| Water | 25 |
| Ligand | 195 |
| 1,2-Ethanediol | 4 |
| Deviation from ideal geometry: | |
| Bond lengths [Å] | 0.011 |
| Bond angles [°] | 1.58 |
| Bonded B's [$Å^2$] | 5.7 |
| Ramachandran plot: | |
| Most favored regions [%] | 93.2 |
| Additional allowed regions [%] | 6.2 |
| Generously allowed regions [%] | 0.6 |
| Disallowed regions [%] | 0.0 |

**Table S7.** Data collection, processing, and refinement statistics for **12** structure

| | |
|---|---|
| Ligand | **12** |
| PDB Code | 8ER7 |
| X-ray source | PXII/X10SA (SLS[1]) |
| Wavelength [Å] | 0.9999 |
| Detector | PILATUS 6M |
| Temperature [K] | 100 |

| | |
|---|---|
| Space group | P 64 2 2 |
| Cell: a; b; c; [Å], α; β; γ; [◦] | 125.08; 125.08; 253.41, 90.0; 90.0; 120.0 |
| Resolution [Å] | 108.32 - 2.81 (3.32-3.07) |
| Unique reflections | 21756 (4440) |
| Multiplicity | 4.0 (4.2) |
| Completeness [%] | 95.8 (96.1) |
| Rsym [%] | 7.2 (42.7) |
| Rmeas [%] | 8.2 (48.6) |
| Mean(I)/sd | 11.06 (2.88) |
| Number of reflections (working /test) | 21267 / 489 |
| Rcryst [%] | 24.4 |
| Rfree[%] | 29.7 |
| Total number of atoms: | |
| Protein | 4903 |
| Water | 4 |
| Ligand | 198 |
| Chloride | 2 |
| Deviation from ideal geometry: | |
| Bond lengths [Å] | 0.011 |
| Bond angles [°] | 1.35 |
| Bonded B's [$Å^2$] | 2.8 |
| Ramachandran plot: | |
| Most favored regions [%] | 89.3 |
| Additional allowed regions [%] | 10.7 |
| Generously allowed regions [%] | 0.0 |
| Disallowed regions [%] | 0.0 |

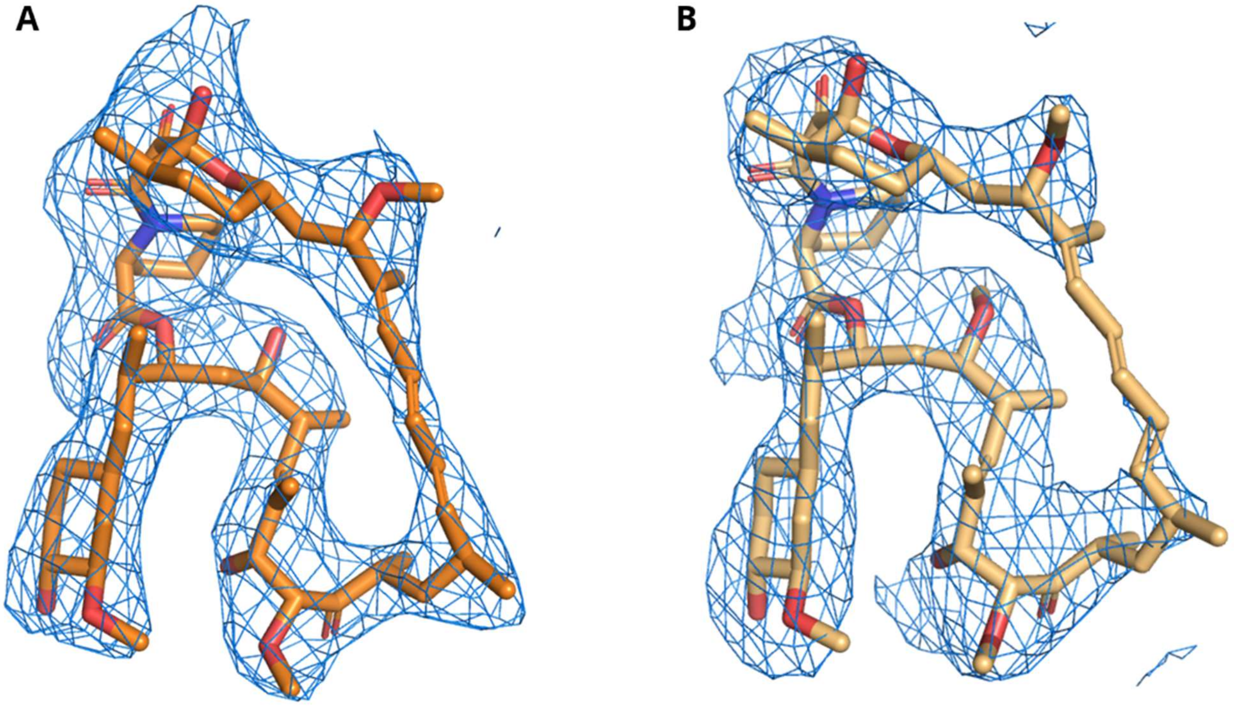


**Figure S3.** The unbiased omit maps (Fo – Fc) of **11** (A) and **12** (B). Maps are obtained upon refinement of the whole model sans ligands and are displayed at 2.0σ.

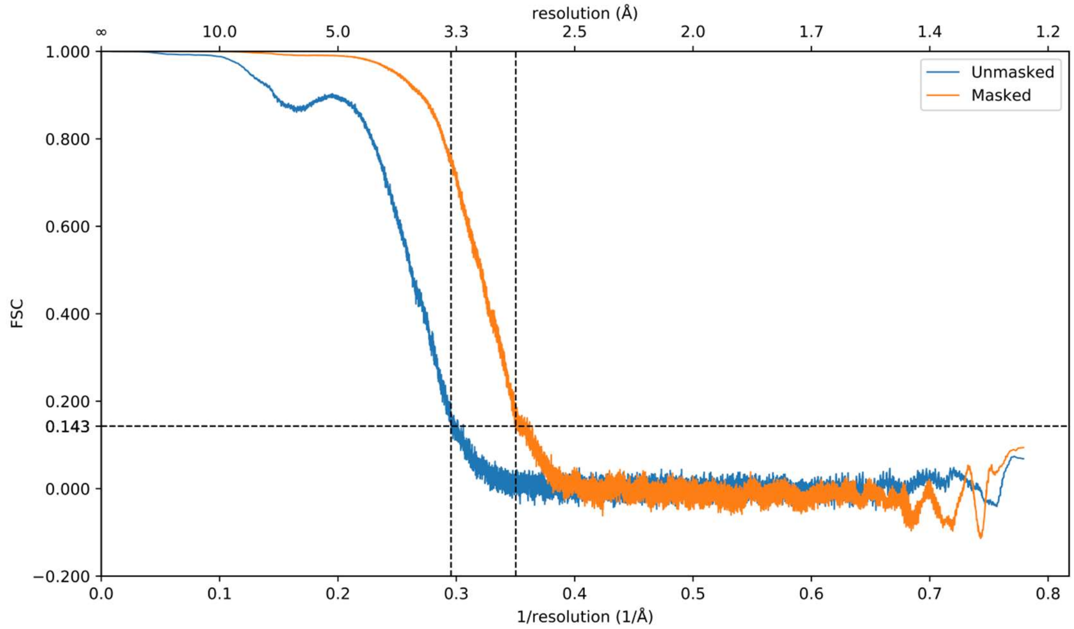


**Figure S4.** Fourier shell correlation of independent half maps of mTORC1-RMC-5552-FKBP12 cryo-EM dataset. FSC gold standard indicates a resolution of 2.9 Å for masked, and 3.4 Å unmasked (PDB 8ERA).

**Table S8.** Nanosyn Lipid Kinase Panel Data

| | **mTORC1 $IC_{50}$ (µM)** | **DNA-PK $IC_{50}$ (µM)** | **PI3K-ALPHA $IC_{50}$ (µM)** | **PI3K-BETA $IC_{50}$ (µM)** | **PI3K-DELTA $IC_{50}$ (µM)** | **PI3K-GAMMA $IC_{50}$ (µM)** |
|---|---|---|---|---|---|---|
| RMC-5552 **38** | 0.000774 | >1 | 0.0412 | >1 | 0.095 | >1 |
| RMC-6272 **40** | 0.000473 | >1 | >1 | >1 | >1 | >1 |

**Table S9.** ActivX KiNativ Kinase Panel Data for RMC-5552 **38** and RMC-6272 **40** in MCF-7 Cells

| **Kinase** | **Sequence** | **Labeling Site** | **RMC-5552 38** | **RMC-6272 40** |
|---|---|---|---|---|
| FRAP (mTOR) | IQSIAPSLQVITSKQRPR | ATP | 97.4 | 97.2 |
| ABL, ARG | YSLTVAVKTLKEDTMEVEEFLK | Lys1 | -1.3 | -25.3 |
| ABL, ARG | LMTGDTYTAHAGAKFPIK | Activation Loop | -3.4 | 16.3 |

| | | | | |
|---|---|---|---|---|
| ACK | KVPFAWCAPESLK | Activation Loop | -1.8 | 16.2 |
| ACK | TVSVAVKCLKPDVLSQPEAMDDFIR | Lys1 | -16.4 | -25.4 |
| AGK | ATVFLNPAACKGK | ATP | -0.8 | -31.9 |
| AKT1 | GTFGKVILVK | ATP Loop | -30.5 | -53.8 |
| AKT2, AKT3 | GTFGKVILVR | ATP Loop | -13.5 | -101.1 |
| AMPKa1 | IGHYILGDTLGVGTFGKVK | ATP Loop | 6.5 | -10.8 |
| AMPKa1, AMPKa2 | VAVKILNR | Lys1 | 6.8 | 2.1 |
| AMPKa1, AMPKa2 | DLKPENVLLDAHMNAK | Lys2 | -6.5 | -1.3 |
| ARAF | DLKSNNIFLHEGLTVK | Lys2 | -6.8 | -9.1 |
| ATR | FYIMMCKPK | ATP | -3.5 | -7.7 |
| AurA | DIKPENLLLGSAGELK | Lys2 | 26.5 | -24.5 |
| AurA, AurB, AurC | GKFGNVYLAR | ATP Loop | 34.8 | -3.6 |
| BARK1 | DLKPANILLDEHGHVR | Lys2 | 8.1 | 14.9 |
| BLK | VAIKTLK | Lys1 | 14.1 | -6.2 |
| BRAF | DLKSNNIFLHEDLTVK | Lys2 | -1.0 | -8.9 |
| CaMK1a | LVAIKCIAK | Lys1 | 12.2 | -4.8 |
| CaMK1d | LFAVKCIPK | Lys1 | 12.7 | 6.4 |
| CaMK1d | DLKPENLLYYSQDEESK | Lys2 | 1.6 | -4.0 |
| CaMK2a, CaMK2b, CaMK2d, CaMK2g | DLKPENLLLASK | Lys2 | 26.2 | -30.6 |
| CaMK2d | IPTGQEYAAKIINTKK | Lys1 | 16.5 | -1.7 |
| CaMK2g | TSTQEYAAKIINTK | Lys1 | -6.6 | 1.9 |
| CaMK4 | DLKPENLLYATPAPDAPLK | Lys2 | -2.4 | 9.5 |
| CaMKK1 | DIKPSNLLLGDDGHVK | Lys2 | 14.1 | 10.6 |
| CaMKK2 | DIKPSNLLVGEDGHIK | Lys2 | 17.8 | -8.2 |
| CASK | ETGQQFAVKIVDVAK | Lys1 | -3.9 | -31.4 |
| CCRK | DLKPANLLISASGQLK | Lys2 | -5.4 | -28.5 |
| CDC2 | DLKPQNLLIDDK | Lys2 | -0.7 | -6.5 |
| CDC2 | TTGQVVAMKK | Lys1 | -32.6 | -18.5 |
| CDK11, CDK8 | DLKPANILVMGEGPER | Lys2 | -39.0 | -19.1 |
| CDK2 | DLKPQNLLINTEGAIK | Lys2 | 13.1 | -40.9 |
| CDK2 | LTGEVVALKK | Lys1 | 1.0 | -29.1 |
| CDK4 | DLKPENILVTSGGTVK | Lys2 | 5.8 | -17.8 |
| CDK5 | DLKPQNLLINR | Lys2 | 9.0 | -29.6 |
| CDK5 | NRETHEIVALKR | Lys1 | 5.2 | -21.6 |
| CDK6 | DLKPQNILVTSSGQIK | Lys2 | -5.7 | -15.2 |
| CDK7 | DKNTNQIVAIKK | Lys1 | 14.8 | -2.4 |
| CDK7 | DLKPNNLLLDENGVLK | Lys2 | 10.3 | -32.1 |

| | | | | |
|---|---|---|---|---|
| CDK9 | IGQGTFGEVFKAR | ATP Loop | 1.4 | -44.8 |
| CDK9 | DMKAANVLITR | Lys2 | -6.5 | -14.6 |
| CHK1 | LSKGDGLEFK | Other | 25.4 | 19.6 |
| CHK1 | DIKPENLLLDER | Lys2 | -13.6 | -10.9 |
| CHK2 | DLKPENVLLSSQEEDCLIK | Lys2 | 34.9 | 5.4 |
| CHK2 | VAIKIISK | Lys1 | 14.8 | 9.4 |
| CK1a | DIKPDNFLMGIGR | Lys2 | -12.3 | -14.1 |
| CK1g1 | DVKPENFLIGR | Lys2 | 4.7 | 1.3 |
| CK1g1, CK1g2, CK1g3 | KIGCGNFGELR | ATP Loop | 8.9 | -7.8 |
| CK1g3 | DVKPENFLIGRPGNK | Lys2 | 13.2 | -6.6 |
| CK2a1 | GGPNIITLADIVKDPVSR | Protein Kinase Domain | 6.4 | 18.8 |
| CSK | VAVKCIK | Lys1 | 13.6 | 17.7 |
| CSK | VSDFGLTKEASSTQDTGKLPVK | Activation Loop | 1.9 | 5.5 |
| DGKA | IDPVPNTHPLLVFVNPKSGGK | ATP | 6.9 | 6.4 |
| DGKH | ATFSFCVSPLLVFVNSKSGDNQGVK | ATP | 9.7 | -15.3 |
| DLK | DLKSPNMLITYDDVVK | Lys2 | -43.9 | -20.3 |
| DNAPK | KGGSWIQEINVAEK | ATP | 25.9 | -26.4 |
| DNAPK | EHPFLVKGGEDLR | ATP | 8.9 | -0.4 |
| eEF2K | YIKYNSNSGFVR | ATP | -10.4 | -40.7 |
| EGFR | LLGAEEKEYHAEGGKVPIK | Activation Loop | -1.8 | -40.0 |
| EGFR | IPVAIKELR | Lys1 | -7.4 | -27.8 |
| EphA1 | LLDDFDGTYETQGGKIPIR | Activation Loop | 23.8 | 4.8 |
| EphA2 | VLEDDPEATYTTSGGKIPIR | Activation Loop | 26.0 | 25.1 |
| EphB2 | FLEDDTSDPTYTSALGGKIPIR | Activation Loop | 26.5 | 1.3 |
| EphB4 | FLEENSSDPTYTSSLGGKIPIR | Activation Loop | 12.9 | 20.9 |
| Erk1 | DLKPSNLLINTTCDLK | Lys2 | 2.8 | -41.1 |
| Erk2 | DLKPSNLLLNTTCDLK | Lys2 | 3.2 | -15.7 |
| Erk3 | DLKPANLFINTEDLVLK | Lys2 | 27.6 | 34.8 |
| Erk5 | DLKPSNLLVNENCELK | Lys2 | -31.3 | -11.4 |
| FAK | CIGEGQFGDVHQGIYMSPENPALAVAIKTCK | Lys1 | -7.4 | -2.1 |
| FAK | YMEDSTYYKASK | Activation Loop | -18.5 | -1.4 |
| FAM20B | ETEPACADGDIMEGSVTLWLPDVWPLQKHR | ATP | -46.1 | -7.6 |
| FER | TSVAVKTCKEDLPQELK | Lys1 | 9.4 | -7.2 |
| FER | QEDGGVYSSSGLKQIPIK | Activation Loop | -2.6 | 1.7 |

| | | | | |
|---|---|---|---|---|
| FRK | HEIKLPVK | Activation Loop | -2.8 | -34.9 |
| FYN, SRC, YES | QGAKFPIKWTAPEAALYGR | Activation Loop | -6.3 | -28.5 |
| GCK | DIKGANLLLTLQGDVK | Lys2 | -5.3 | -23.1 |
| GCN2_Domain2 domain2 | DLKPVNIFLDSDDHVK | Lys2 | 18.6 | 9.9 |
| GCN2_Domain2 domain2 | LDGCCYAVKR | Lys1 | -9.3 | -15.5 |
| GSK3A | DIKPQNLLVDPDTAVLK | Lys2 | -10.7 | -35.9 |
| GSK3B | DIKPQNLLLDPDTAVLK | Lys2 | 2.1 | -23.6 |
| HER2/ErbB2 | LLDIDETEYHADGGKVPIK | Activation Loop | 14.3 | -8.7 |
| HER2/ErbB2 | GIWIPDGENVKIPVAIKVLR | Lys1 | 2.2 | -22.5 |
| HER3/ErbB3 | GVWIPEGESIKIPVCIKVIEDK | Lys1 | -6.5 | -22.1 |
| HPK1 | DKVSGDLVALKMVK | Lys1 | -5.1 | 19.0 |
| HPK1 | DIKGANILINDAGEVR | Lys2 | -9.7 | 2.1 |
| HRI | IGDFGLACTDILQKNTDWTNR | Activation Loop | 0.3 | -12.7 |
| IKKa | DLKPENIVLQDVGGK | Lys2 | 10.2 | -41.7 |
| IKKb | DLKPENIVLQQGEQR | Lys2 | 20.9 | 17.0 |
| IKKe, TBK1 | DIKPGNIMR | Lys2 | -33.9 | -21.1 |
| ILK | WQGNDIVVKVLK | Lys1 | -11.9 | -40.5 |
| ILK | ISMADVKFSFQCPGR | Protein Kinase Domain | -14.6 | 6.2 |
| IRAK1 | AIQFLHQDSPSLIHGDIKSSNVLLDER | Lys2 | 24.6 | 7.3 |
| IRAK4 | GYVNNTTVAVKK | Lys1 | -7.0 | -35.0 |
| IRAK4 | DIKSANILLDEAFTAK | Lys2 | -19.3 | -35.4 |
| IRE1 | DLKPHNILISMPNAHGK | Lys2 | -42.8 | -32.1 |
| ITPK1 | NFSAGTSDRESIFFNSHNVSKPESSSVLTELDKIEGVFERPSDEVIR | ATP | 22.4 | -12.4 |
| ITPK1 | ESIFFNSHNVSKPESSSVLTELDKIEGVFERPSDEVIR | ATP | 6.5 | -9.6 |
| JAK1 domain1 | QLASALSYLEDKDLVHGNVCTKNLLLAR | Activation Loop | 0.8 | -1.7 |
| JAK1_Domain2 domain2 | IGDFGLTKAIETDKEYYTVK | Activation Loop | 31.3 | 19.5 |
| JAK1_Domain2 domain2 | YDPEGDNTGEQVAVKSLKPESGGNHIADLKK | Lys1 | 26.2 | 13.3 |
| JNK1, JNK2, JNK3 | DLKPSNIVVK | Lys2 | 13.8 | -9.5 |
| KSR1, KSR2 | SKNVFYDNGK | Activation Loop | -19.2 | -20.7 |
| LATS1 | ALYATKTLR | Lys1 | 12.7 | -6.7 |
| LATS1 | DIKPDNILIDR | Lys2 | -15.4 | -18.1 |
| LATS2 | DIKPDNILIDLDGHIK | Lys2 | 22.9 | -1.4 |
| LATS2 | VDTHALYAMKTLR | Lys1 | -20.5 | -0.8 |

| | | | | |
|---|---|---|---|---|
| LCK | EGAKFPIKWTAPEAINYGTFTIK | Activation Loop | -0.7 | 14.2 |
| LCK | VAVKSLK | Lys1 | -12.3 | 31.0 |
| LKB1 | DIKPGNLLLTTGGTLK | Lys2 | 9.7 | -26.4 |
| LOK | DLKAGNVLMTLEGDIR | Lys2 | -65.4 | -19.4 |
| MAP2K1 | IMHRDVKPSNILVNSR | Lys2 | -4.5 | 6.2 |
| MAP2K1, MAP2K2 | KLIHLEIKPAIR | Lys1 | 14.8 | -34.9 |
| MAP2K1, MAP2K2 | DVKPSNILVNSR | Lys2 | 7.6 | -22.1 |
| MAP2K2 | HQIMHRDVKPSNILVNSR | Lys2 | -27.5 | -9.7 |
| MAP2K3 | DVKPSNVLINK | Lys2 | 23.9 | -13.0 |
| MAP2K3 | HAQSGTIMAVKR | Lys1 | -4.0 | 5.5 |
| MAP2K4 | DIKPSNILLDR | Lys2 | 10.0 | -0.7 |
| MAP2K4 | MVHKPSGQIMAVKR | Lys1 | -36.3 | -32.8 |
| MAP2K5 | DVKPSNMLVNTR | Lys2 | -27.3 | -37.7 |
| MAP2K6 | DVKPSNVLINALGQVK | Lys2 | 17.1 | -26.0 |
| MAP2K6 | HVPSGQIMAVKR | Lys1 | -1.4 | 11.7 |
| MAP2K7 | DVKPSNILLDER | Lys2 | 20.9 | -14.8 |
| MAP3K1 | DVKGANLLIDSTGQR | Lys2 | 22.7 | -43.5 |
| MAP3K15, MAP3K5, MAP3K6 | IAIKEIPER | Lys1 | 24.5 | -4.5 |
| MAP3K2 | ELAVKQVQFDPDSPETSKEVNALECEIQLLK | Lys1 | 22.6 | 30.9 |
| MAP3K2, MAP3K3 | DIKGANILR | Lys2 | -12.6 | -18.2 |
| MAP3K3 | ELASKQVQFDPDSPETSKEVSALECEIQLLK | Lys1 | -0.6 | -29.7 |
| MAP3K4 | DIKGANIFLTSSGLIK | Lys2 | -20.8 | -16.3 |
| MAP3K4 | VYTCISVDTGELMAMKEIR | Lys1 | -22.2 | 8.5 |
| MAP3K5 | DIKGDNVLINTYSGVLK | Lys2 | 6.5 | -35.1 |
| MAP3K6 | DIKGDNVLINTFSGLLK | Lys2 | 33.6 | 15.3 |
| MAP4K3 | NVNTGELAAIKVIK | Lys1 | 12.2 | -19.2 |
| MAP4K3 | DIKGANILLTDNGHVK | Lys2 | -6.9 | 69.9 |
| MAP4K5 | DIKGANILLTDHGDVK | Lys2 | 17.9 | -1.6 |
| MAP4K5 | NVHTGELAAVKIIK | Lys1 | 16.0 | 5.5 |
| MAPKAPK2, MAPKAPK3 | DVKPENLLYTSK | Lys2 | -19.9 | -27.1 |
| MAPKAPK3 | QVLGLGVNGKVLECFHR | ATP Loop | -2.7 | -4.2 |
| MARK2 | EVAVKIIDKTQLNSSSLQK | Lys1 | 11.5 | 14.0 |
| MARK2, MARK3 | DLKAENLLLDADMNIK | Lys2 | -0.4 | 15.7 |
| MARK3 | EVAIKIIDKTQLNPTSLQK | Lys1 | 31.6 | 1.4 |
| MARK3, MARK4 | EVAIKIIDK | Lys1 | 7.2 | 13.2 |

| | | | | |
|---|---|---|---|---|
| MARK4 | DLKAENLLLDAEANIK | Lys2 | 20.6 | 22.2 |
| MARK4 | EVAIKIIDKTQLNPSSLQK | Lys1 | 7.0 | 24.2 |
| MAST1, MAST2 | DLKPDNLLITSMGHIK | Lys2 | 19.6 | 22.5 |
| MAST3 | DLKPDNLLITSLGHIK | Lys2 | 29.0 | 11.8 |
| MASTL | LYAVKVVK | Lys1 | 17.4 | -10.0 |
| MASTL | GAFGKVYLGQK | ATP Loop | 4.3 | -12.4 |
| MELK | DLKPENLLFDEYHK | Lys2 | 14.9 | 5.1 |
| MER, TYRO3 | KIYSGDYYR | Activation Loop | -0.7 | 7.1 |
| MET | TGAKLPVK | Activation Loop | 3.9 | 6.3 |
| MLK1 | DLKSSNILILQK | Lys2 | 12.8 | -34.0 |
| MLK3 | DLKSNNILLLQPIESDDMEHK | Lys2 | 2.0 | -12.3 |
| MLK4 | DLKSSNILLLEK | Lys2 | -22.4 | -13.4 |
| MLKL | VKSTAYLSPQELEDVFYQYDVK | Activation Loop | 27.6 | 15.5 |
| MLKL | APVAIKVFK | Lys1 | 18.2 | 14.9 |
| MPSK1 | DLKPTNILLGDEGQPVLMDLGSMNQACI HVEGSR | Lys2 | -77.6 | -18.6 |
| MRCKb | NHHVHLYPWSSLDGAEGSFDIKLPETK | Other | -8.5 | -26.5 |
| MSK1 domain1 | DIKLENILLDSNGHVVLTDFGLSK | Lys2 | 17.8 | 27.6 |
| MSK1 domain1, MSK2 domain1 | VLGTGAYGKVFLVR | ATP Loop | 33.6 | 16.0 |
| MSK2 domain1 | DLKLENVLLDSEGHIVLTDFGLSK | Lys2 | 12.6 | 3.3 |
| MSK2 domain1 | LYAMKVLR | Lys1 | -22.4 | -10.1 |
| MST1 | ETGQIVAIKQVPVESDLQEIIK | Lys1 | 10.7 | 18.2 |
| MST1, MST2 | DIKAGNILLNTEGHAK | Lys2 | 3.0 | -6.5 |
| MST2 | ESGQVVAIKQVPVESDLQEIIK | Lys1 | 15.8 | 14.3 |
| MST3 | DIKAANVLLSEHGEVK | Lys2 | 22.2 | 11.0 |
| MST3, MST4, YSK1 | LADFGVAGQLTDTQIKR | Activation Loop | 12.4 | 20.4 |
| MST4, YSK1 | DIKAANVLLSEQGDVK | Lys2 | 10.5 | -10.1 |
| NDR1 | DIKPDNLLLDSK | Lys2 | 9.8 | 3.1 |
| NDR1 | DTGHVYAMKILR | Lys1 | -8.8 | 17.6 |
| NDR1, NDR2 | LSDFGLCTGLKK | Activation Loop | 25.0 | -18.5 |
| NDR2 | DIKPDNLLLDAK | Lys2 | 15.7 | -4.6 |
| NDR2 | DTGHIYAMKILR | Lys1 | -6.2 | 10.8 |
| NEK1 | DIKSQNIFLTK | Lys2 | 2.6 | -29.7 |
| NEK2 | DLKPANVFLDGK | Lys2 | 10.9 | 12.3 |
| NEK3 | SKNIFLTQNGK | Activation Loop | 1.4 | -25.4 |
| NEK4 | DLKTQNVFLTR | Lys2 | -2.6 | -31.0 |
| NEK6, NEK7 | DIKPANVFITATGVVK | Lys2 | 11.1 | -12.6 |
| NEK7 | AACLLDGVPVALKK | Lys1 | -26.1 | -43.1 |

| | | | | |
|---|---|---|---|---|
| NEK8 | DLKTQNILLDK | Lys2 | 10.4 | -12.1 |
| NEK9 | LGDYGLAKK | Activation Loop | 18.0 | 10.2 |
| NuaK2 | LVAIKSIR | Lys1 | 18.8 | 16.0 |
| p38a | DLKPSNLAVNEDCELK | Lys2 | -4.3 | -6.8 |
| p38a | QELNKTIWEVPER | Other | -5.2 | -1.6 |
| p38b | QELNKTVWEVPQR | Other | -23.3 | -42.7 |
| p38d, p38g | DLKPGNLAVNEDCELK | Lys2 | -3.7 | -28.8 |
| p70S6K | DLKPENIMLNHQGHVK | Lys2 | -31.3 | -6.8 |
| p70S6K, p70S6Kb | GGYGKVFQVR | ATP Loop | 20.1 | -4.7 |
| p70S6Kb | IYAMKVLR | Lys1 | -29.0 | 1.4 |
| p70S6Kb | DLKPENIMLSSQGHIK | Lys2 | -36.2 | -26.7 |
| PAK2 | KNPQAVLDVLKFYDSNTVK | Other | -0.2 | -14.7 |
| PAK2 | IGQGASGTVFTATDVALGQEVAIKQINLQK | Lys1 | -21.7 | -31.5 |
| PAN3 | IQKSSNFGYITSCYK | ATP | -15.9 | -43.1 |
| PAN3 | VMDPTKILITGK | ATP | -29.6 | -17.9 |
| PCTAIRE1 | SKLTDNLVALKEIR | Lys1 | 13.1 | -0.2 |
| PCTAIRE1, PCTAIRE3 | DLKPQNLLINER | Lys2 | 13.9 | -31.2 |
| PCTAIRE2 | DLKPQNLLINEK | Lys2 | 17.2 | -4.7 |
| PDK1 | EYAIKILEK | Lys1 | 21.6 | 20.2 |
| PEK | DLKPSNIFFTMDDVVK | Lys2 | -42.9 | -29.1 |
| PFTAIRE2 | DLKPQNLLISHLGELK | Lys2 | -3.5 | 23.9 |
| PHKg2 | ATGHEFAVKIMEVTAER | Lys1 | -1.0 | 1.0 |
| PI4KA, PI4KAP2 | SGTPMQSAAKAPYLAK | ATP | -38.9 | -5.7 |
| PI4KB | VPHTQAVVLNSKDK | ATP | 12.9 | 6.0 |
| PI4KB | LLSVIVKCGDDLRQELLAFQVLK | ATP | -5.0 | -7.2 |
| PIK3C3 | TEDGGKYPVIFKHGDDLR | ATP | 2.9 | -18.6 |
| PIK3CB | VFGEDSVGVIFKNGDDLR | ATP | -15.7 | -37.6 |
| PIK3CB | VFGEDSVGVIFKNGDDLRQDMLTLQMLR | ATP | -60.1 | -31.0 |
| PIP4K2A | AKELPTLKDNDFINEGQK | ATP | 1.7 | 15.0 |
| PIP4K2B | AKDLPTFKDNDFLNEGQK | ATP | -2.7 | 11.0 |
| PIP4K2C | TLVIKEVSSEDIADMHSNLSNYHQYIVK | ATP | 0.4 | 5.4 |
| PIP4K2C | VKELPTLKDMDFLNK | ATP | -18.4 | 8.3 |
| PIP5K3 | GGKSGAAFYATEDDRFILK | ATP | 0.8 | 19.9 |
| PITSLRE | DLKTSNLLLSHAGILK | Lys2 | 17.8 | 13.7 |
| PKCa, PKCg | NLIPMDPNGLSDPYVKLK | Other | -20.9 | 31.7 |
| PKCd | KPTMYPEWK | Other | -39.7 | 2.2 |
| PKCi | DLKLDNVLLDSEGHIK | Lys2 | -20.0 | -12.7 |
| PKCi | IYAMKVVK | Lys1 | -24.1 | -7.2 |
| PKN1 | DLKLDNLLLDTEGYVK | Lys2 | 3.1 | 21.6 |

| | | | | |
|---|---|---|---|---|
| PKN2 | DLKLDNLLLDTEGFVK | Lys2 | 24.3 | 10.7 |
| PKR | TYVIKR | Lys1 | 15.2 | 9.6 |
| PKR | IGDFGLVTSLKNDGKR | Activation Loop | 12.1 | 17.4 |
| PKR | DLKPSNIFLVDTK | Lys2 | 11.0 | -32.5 |
| PLK1 | CFEISDADTKEVFAGKIVPK | Lys1 | 18.5 | 1.1 |
| PLK1 | DLKLGNLFLNEDLEVK | Lys2 | -1.7 | -27.9 |
| PRP4 | CNILHADIKPDNILVNESK | Lys2 | -1.1 | -39.8 |
| PRPK | FLSGLELVKQGAEAR | ATP Loop | -2.8 | 10.5 |
| RIPK3 | DLKPSNVLLDPELHVK | Lys2 | -20.3 | 15.8 |
| ROCK1 | KLQLELNQER | Other | -5.7 | -45.1 |
| ROCK1, ROCK2 | VYAMKLLSK | Lys1 | 20.5 | -42.0 |
| RSK1 domain1 | LTDFGLSKEAIDHEKK | Activation Loop | 21.0 | 15.5 |
| RSK1 domain1, RSK2 domain1, RSK3 domain1 | DLKPENILLDEEGHIK | Lys2 | 22.0 | 27.4 |
| RSK1_Domain2 domain2 | DLKPSNILYVDESGNPECLR | Lys2 | 30.0 | 15.5 |
| RSK2 domain1 | LTDFGLSKESIDHEKK | Activation Loop | 25.0 | 22.4 |
| RSK2 domain1 | QLYAMKVLK | Lys1 | -19.7 | -8.4 |
| RSK2_Domain2 domain2 | DLKPSNILYVDESGNPESIR | Lys2 | 21.2 | 19.2 |
| RSK3 domain1 | DLKPENILLDEEGHIKITDFGLSK | Lys2 | 29.3 | 9.7 |
| RSKL1 | VLGVIDKVLLVMDTR | ATP | -35.4 | -16.6 |
| SGK3 | FYAVKVLQK | Lys1 | 5.6 | -15.8 |
| SLK | AQNKETSVLAAAKVIDTK | Lys1 | 17.3 | 6.9 |
| SLK | DLKAGNILFTLDGDIK | Lys2 | -21.6 | -34.9 |
| SMG1 | SYPYLFKGLEDLHLDER | ATP | 21.6 | 11.7 |
| SMG1 | DTVTIHSVGGTITILPTKTKPK | ATP | 0.8 | -11.1 |
| SRPK1 | IIHTDIKPENILLSVNEQYIR | Lys2 | 12.0 | -15.5 |
| SRPK2 | IIHTDIKPENILMCVDDAYVR | Lys2 | -5.7 | 36.3 |
| STLK5 | SVKASHILISVDGK | Lys2 | -5.4 | -7.7 |
| STLK5 | YSVKVLPWLSPEVLQQNLQGYDAK | Activation Loop | -10.5 | -9.5 |
| STLK6 | HTPTGTLVTIKITNLENCNEER | Lys1 | -7.5 | -5.7 |
| SYK | ISDFGLSKALR | Activation Loop | 18.4 | -14.3 |
| SYK | TVAVKILK | Lys1 | 14.0 | -3.7 |
| SYK | AQTHGKWPVK | Activation Loop | 6.4 | 13.3 |
| TAK1 | DLKPPNLLLVAGGTVLK | Lys2 | 9.6 | 11.1 |
| TAO1, TAO3 | DIKAGNILLTEPGQVK | Lys2 | -1.7 | -29.7 |
| TAO2 | DVKAGNILLSEPGLVK | Lys2 | -14.8 | -31.8 |

| | | | | |
|---|---|---|---|---|
| TBK1 | TGDLFAIKVFNNISFLRPVDVQMR | Lys1 | -39.1 | -20.7 |
| TEC | YVLDDQYTSSSGAKFPVK | Activation Loop | -8.2 | -12.7 |
| TLK1 | YLNEIKPPIIHYDLKPGNILLVDGTACGEI K | Lys2 | 19.6 | 10.8 |
| TLK1 | YAAVKIHQLNK | Lys1 | 5.7 | -9.3 |
| TLK2 | YLNEIKPPIIHYDLKPGNILLVNGTACGEI K | Lys2 | 22.7 | 16.3 |
| TLK2 | YVAVKIHQLNK | Lys1 | 14.9 | -3.2 |
| TYK2_Domain2 domain2 | IGDFGLAKAVPEGHEYYR | Activation Loop | 15.6 | 1.9 |
| ULK1 | DLKPQNILLSNPAGR | Lys2 | -11.7 | -15.4 |
| ULK3 | NISHLDLKPQNILLSSLEKPHLK | Lys2 | 11.9 | -5.4 |
| ULK3 | EVVAIKCVAK | Lys1 | 0.5 | -22.8 |
| Wee1 | YIHSMSLVHMDIKPSNIFISR | Lys2 | -30.3 | 12.6 |
| Wnk1, Wnk2 | GSFKTVYK | ATP Loop | 13.6 | -3.7 |
| Wnk1, Wnk2, Wnk3 | DLKCDNIFITGPTGSVK | Lys2 | -22.1 | -40.3 |
| Wnk1, Wnk2, Wnk4 | IGDLGLATLKR | Activation Loop | 23.6 | -17.4 |
| YANK3 | DVKPDNILLDER | Lys2 | 14.6 | -17.1 |
| YES | VAIKTLKPGTMMPEAFLQEAQIMK | Lys1 | -19.7 | -6.6 |
| ZAK | WISQDKEVAVKK | Lys1 | 2.3 | 8.6 |
| ZAP70 | ISDFGLSKALGADDSYYTAR | Activation Loop | -3.2 | 9.7 |
| ZAP70 | SAGKWPLK | Activation Loop | -11.1 | -0.8 |
| ZC1/HGK, ZC2/TNIK, ZC3/MINK | DIKGQNVLLTENAEVK | Lys2 | 8.5 | -13.4 |

**Labeling Site Key**

| | |
|---|---|
| Lys1 | Conserved Lysine 1 |
| Lys2 | Conserved Lysine 2 |
| ATP Loop | ATP binding loop |
| Activation Loop | Activation loop |
| ATP | ATP site in non-canonical kinase (e.g. lipid kinase) |
| Protein Kinase Domain | Other lysine within kinase domain, possibly not in ATP binding site |
| Other | Labeling of residue outside of the protein kinase domain, possibly not in ATP binding site |

**Table S10.** Eurofins Safety Screen[44] Data for RMC-5552 **38**

| Target | % Inhibition of Control Values |
|---|---|
| $A_{2A}$(*h*) (agonist radioligand) | -4.5 |
| $\alpha_{1A}$(*h*) (antagonist radioligand) | 0.6 |
| $\alpha_{2A}$(*h*) (antagonist radioligand) | -2.1 |
| $\beta_1$(*h*) (agonist radioligand) | 3.0 |
| $\beta_2$(*h*) (antagonist radioligand) | -3.3 |
| BZD (central) (agonist radioligand) | 3.3 |
| $CB_1$(*h*) (agonist radioligand) | -26.1 |
| $CB_2$(*h*) (agonist radioligand) | 0.6 |
| $CCK_1$ (CCKA) (*h*) (agonist radioligand) | -51.3 |
| $D_1$(*h*) (antagonist radioligand) | -0.5 |
| $D_{2S}$(*h*) (agonist radioligand) | 12.2 |
| $ET_A$(*h*) (agonist radioligand) | 16.8 |
| NMDA (antagonist radioligand) | 13.7 |
| $H_1$(*h*) (antagonist radioligand) | -12.6 |
| $H_2$(*h*) (antagonist radioligand) | -1.1 |
| MAO-A (antagonist radioligand) | 5.5 |
| $M_1$(*h*) (antagonist radioligand) | -5.4 |
| $M_2$ (*h*) (antagonist radioligand) | -3.0 |
| $M_3$(*h*) (antagonist radioligand) | -14.3 |
| N neuronal α4β2 (*h*) (agonist radioligand) | -8.5 |
| δ (DOP) (*h*) (agonist radioligand) | 11.6 |
| kappa (*h*) (KOP) (agonist radioligand) | 0.6 |
| μ (MOP) (*h*) (agonist radioligand) | 10.3 |
| 5-$HT_{1A}$(*h*) (agonist radioligand) | -9.4 |
| 5-$HT_{1B}$ (*h*) (antagonist radioligand) | 13.1 |
| 5-$HT_{2A}$(*h*) (agonist radioligand) | -28.9 |
| 5-$HT_{2B}$(*h*) (agonist radioligand) | -14.5 |
| 5-$HT_3$(*h*) (antagonist radioligand) | -4.6 |
| GR (*h*) (agonist radioligand) | 21.8 |
| AR (*h*) (agonist radioligand) | -14.9 |
| $V_{1a}$(*h*) (agonist radioligand) | 6.5 |
| $Ca^{2+}$ channel (L, dihydropyridine site) (antagonist radioligand) | -45.0 |
| Potassium Channel hERG (human)- [3H] Dofetilide | -1.2 |
| $K_V$ channel (antagonist radioligand) | -8.0 |
| $Na^+$ channel (site 2) (antagonist radioligand) | 1.0 |
| norepinephrine transporter(*h*) (antagonist radioligand) | -15.7 |

| | |
|---|---|
| dopamine transporter(*h*) (antagonist radioligand) | -1.5 |
| 5-HT transporter (*h*) (antagonist radioligand) | -4.5 |
| COX1(*h*) | 22.1 |
| COX2(*h*) | 4.4 |
| PDE3A (*h*) | -47.2 |
| PDE4D2 (*h*) | -45.6 |
| Lck kinase (*h*) | 11.3 |
| acetylcholinesterase (*h*) | -15.4 |

Safety screen was performed with a RMC-5552 **38** concentration of 10 μM

**References**

[1] Reichling, L. J.; Lebakken, C. S.; Riddle, S. M.; Vedvik, K. L.; Robers, M. B.; Kopp, L. M.; Bruinsma, R.; Vogel, K. W. Pharmacological characterization of purified recombinant mTOR FRB-kinase domain using fluorescence-based assays. *J. Biomol. Screen* **2008**, *13*, 238–244.

[2] Patricelli, M. P.; Szardenings, A. K.; Liyanage, M.; Nomanbhoy, T. K.; Wu, M.; Weissig, H.; Aban, A.; Chun, D.; Tanner, S.; Kozarich, J. W. Functional interrogation of the kinome using nucleotide acyl phosphates. *Biochemistry* **2007**, *46*, 350–358.

[3] Yang, H.; Rudge, D. G.; Koos, J. D.; Vaidialingam, B.; Yang, H. J.; Pavletich, N. P. mTOR kinase structure, mechanism and regulation. *Nature* **2013**, *497*, 217–223.

[4] Liang, J.; Choi, J.; Clardy, J. Refined structure of the FKBP12–rapamycin–FRB ternary complex at 2.2 Å resolution. *Acta Crystallogr. D* **1999**, *55*, 736–744.

[5] Yang, H.; Jiang, X.; Li, B.; Yang, H. J.; Miller, M.; Yang, A.; Dhar, A.; Pavletich, N. P. Mechanisms of mTORC1 activation by RHEB and inhibition by PRAS40. *Nature* **2017**, *552*, 368–373.

[6] Mastronarde, D. N. Automated electron microscope tomography using robust prediction of specimen movements. *J. Struct. Bio.* **2005** *152*, 36–51.

[7] Zivanov, J.; Nakane, T.; Scheres, S. H. W.; Estimation of high-order aberrations and anisotropic magnification from Cryo-EM data sets in RELION-3.1. *IUCrJ*, **2020**, *7*, 253–267.

[8] Zheng, S. Q.; Palovcak, E.; Armache, J-P.; Verba, K. A.; Cheng, Y.; Agard, D. A.; Motioncor2: anisotropic correction of beam-induced motion for improved cryo-electron microscopy. *Nat. Methods* **2017**, *14*, 331–332.

[9] Zhang, K. GCTF: Real-Time CTF determination and correction. *J. Struct. Bio.* **2016**, *193*, 1–12.

[10] Pettersen, E. F.; Goddard, T. D.; Huang, C. C.; Meng, E. C.; Couch, G. S.; Croll, T. I.; Morris, J. H.; Ferrin, T. E. UCSF ChimeraX : structure visualization for researchers, educators, and developers. *Protein Science* **2021**, *30*, 70–82.

[11] Long, F.; Nicholls, R. A.; Emsley, P.; Gražulis, S.; Merkys, A.; Vaitkus, A.; Murshudov, G. N. AceDRG: a stereochemical description generator for ligands. *Acta Crystallogr. D* **2017**, *73*, 112–122.

[12] Liebschner, D.; Afonine, P. V.; Baker, M. L.; Bunkóczi, G.; Chen, V. B.; Croll, T. I.; Hintze, B.; Hung, L-W.; Jain, S.; McCoy, A. J.; Moriarty, N. W.; Oeffner, R. D.; Poon, B. K.; Prisant, M. G.; Read, R. J.; Richardson, J. S.; Richardson, D. C.; Sammito, M. D.; Sobolev, O. V.; Stockwell, D. H.; Terwilliger, T. C.; Urzhumtsev, A. G.; Videau, L. L.; Williams, C. J.; Adams,

P. D. Macromolecular structure determination using x-rays, neutrons and electrons: recent developments in Phenix. *Acta Crystallogr D* **2019**, *75*, 861–877.

[13] The PyMOL Molecular Graphics System, Version 2.4 Schrödinger, LLC.
